# CNV Analysis in Tourette Syndrome Implicates Large Genomic Rearrangements in *COL8A1* and *NRXN1*

**DOI:** 10.1371/journal.pone.0059061

**Published:** 2013-03-22

**Authors:** Abhishek Nag, Elena G. Bochukova, Barbara Kremeyer, Desmond D. Campbell, Heike Muller, Ana V. Valencia-Duarte, Julio Cardona, Isabel C. Rivas, Sandra C. Mesa, Mauricio Cuartas, Jharley Garcia, Gabriel Bedoya, William Cornejo, Luis D. Herrera, Roxana Romero, Eduardo Fournier, Victor I. Reus, Thomas L. Lowe, I. Sadaf Farooqi, the Tourette Syndrome Association International Consortium for Genetics, Carol A. Mathews, Lauren M. McGrath, Dongmei Yu, Ed Cook, Kai Wang, Jeremiah M. Scharf, David L. Pauls, Nelson B. Freimer, Vincent Plagnol, Andrés Ruiz-Linares

**Affiliations:** 1 UCL Genetics Institute, Department of Genetics, Evolution and Environment, University College London, London, United Kingdom; 2 University of Cambridge Metabolic Research Laboratories, Institute of Metabolic Science, Addenbrooke’s Hospital, Cambridge, United Kingdom; 3 Laboratorio de Genética Molecular, SIU, Universidad de Antioquia, Medellín, Colombia; 4 Escuela de Ciencias de la Salud, Universidad Pontificia Bolivariana, Medellín, Colombia; 5 Departamento de Pediatría, Facultad de Medicina, Universidad de Antioquia, Medellín, Colombia; 6 Hospital Nacional de Niños, San José, Costa Rica; 7 Department of Psychiatry, University of California San Francisco, San Francisco, California, United States of America; 8 Psychiatric and Neurodevelopmental Genetics Unit, Center for Human Genetics Research, Boston, Massachusetts, United States of America; 9 Department of Psychiatry, Massachusetts General Hospital, Boston, Massachusetts, United States of America; 10 University of Illinois, Chicago, Illinois, United States of America; 11 University of Southern California, Los Angeles, California, United States of America; 12 Department of Neurology, Massachusetts General Hospital, Boston, Massachusetts, United States of America; 13 Center for Neurobehavioral Genetics, University of California Los Angeles, Los Angeles, California, United States of America; Cincinnati Children’s Hospital Medical Center, United States of America

## Abstract

Tourette syndrome (TS) is a neuropsychiatric disorder with a strong genetic component. However, the genetic architecture of TS remains uncertain. Copy number variation (CNV) has been shown to contribute to the genetic make-up of several neurodevelopmental conditions, including schizophrenia and autism. Here we describe CNV calls using SNP chip genotype data from an initial sample of 210 TS cases and 285 controls ascertained in two Latin American populations. After extensive quality control, we found that cases (N = 179) have a significant excess (*P* = 0.006) of large CNV (>500 kb) calls compared to controls (N = 234). Amongst 24 large CNVs seen only in the cases, we observed four duplications of the *COL8A1* gene region. We also found two cases with ∼400kb deletions involving *NRXN1*, a gene previously implicated in neurodevelopmental disorders, including TS. Follow-up using multiplex ligation-dependent probe amplification (and including 53 more TS cases) validated the CNV calls and identified additional patients with rearrangements in *COL8A1* and *NRXN1,* but none in controls. Examination of available parents indicates that two out of three *NRXN1* deletions detected in the TS cases are *de-novo* mutations. Our results are consistent with the proposal that rare CNVs play a role in TS aetiology and suggest a possible role for rearrangements in the *COL8A1* and *NRXN1* gene regions.

## Introduction

TS is a childhood onset neuropsychiatric illness characterised by the occurrence of multiple, motor and vocal tics and is often associated with obsessive-compulsive disorder (OCD) and attention-deficit hyperactivity disorder (ADHD) [1]–[5]. Twin studies have estimated a sibling relative risk ratio for TS of about 6–8 [2], one of the highest amongst neuropsychiatric disorders. However, identification of genetic variants underlying TS has proven difficult [5]–[7]. Genome-wide linkage and candidate gene association studies have failed to provide robust evidence implicating specific loci, and a recent GWAS has not identified common variants associated with TS at genome-wide significance thresholds [8]. The observation of chromosomal abnormalities in TS families [9]–[11] has suggested the possibility that genomic rearrangements could play an important role in this disorder, but prior studies have provided conflicting evidence regarding the involvement of copy number variants (CNVs) in TS [12], [13]. To further evaluate the role of CNVs in TS, we performed a genomewide study of CNVs in a case/control sample from two well-studied, closely related Latin American population isolates.

### Ethics Statement

This research was approved by the BioEthics Committee of Universidad de Antioquia (Colombia) and the NHS National Research Ethics Service, Central London Committee REC 4 (UK). Written consent was obtained from all subjects. In the case of minors, written consent was obtained from a parent or legal guardian.

### Patients and Methods

We studied CNVs in a sample of 210 unrelated TS cases ascertained in two closely related Latin American population isolates and 285 unrelated population controls. The populations of Antioquia, Colombia, and of the Central Valley of Costa Rica (CVCR) have similar and partly shared demographic histories and are genetically closely related [14], [15]. They are therefore expected to show an enrichment for shared predisposing factors for complex genetic conditions, including TS [14]–[17]. Of the cases, 81 were recruited at the Neuropaediatrics Clinic of Hospital Universitario San Vicente de Paúl (Antioquia, Colombia) and 129 were recruited at Hospital Nacional de Niños (San José, Costa Rica). Diagnosis was based on DSM-IV criteria, focusing on narrowly defined moderate to severe TS. The mean age of cases was 13 years, with a mean age for the start of symptoms at 6.4 years. In addition to TS, 48% of the cases have a diagnosis of ADHD and 53% have OCD. An additional set of 53 TS cases used for MLPA-based follow-up (see below) was also recruited through the Neuropaediatrics Clinic of Hospital Universitario San Vicente de Paúl (Antioquia, Colombia), following the same diagnostic procedures. Population controls were obtained in Antioquia as part of on-going genetic diversity studies in the region [18]. For both, cases and controls, genealogical enquiries confirmed local ancestry in at least 6/8 great-grandparents. Because matched population controls from the CVCR were unavailable, and based on the close genetic relatedness of Antioquia and the CVCR, Antioquian controls were contrasted with Antioquian and Costa Rican cases accounting for stratification (see below). All samples were genotyped using Illumina Human660 arrays as part of the TSAICG genome-wide association study of TS [8].

We obtained CNV calls from the raw hybridization intensities using PennCNV [19]. We excluded from this analysis samples that were outliers based on either the variability of the raw intensity data (using the standard deviation of the logR ratio), or on the total number of CNVs called (see Methods S1 and Figure S2). This resulted in 413 samples being retained for further analysis (179 cases and 234 controls). To make the final CNV calls, we used the following criteria: (i) we merged neighbouring CNVs when the distance separating them was less than half of the total distance from the start of the first CNV to the end of the second CNV, (ii) we only called CNVs containing at least 10 SNPs, and (iii) we ignored CNVs located in centromeric and telomeric regions.

The CNV burden for each sample was determined by counting all CNVs and stratifying them by size into four categories: <10 kb, 10–100 kb, 100–500 kb and >500 kb. All calls for CNVs >500 kb (“large CNVs”) were confirmed individually by plotting the LogR ratio and B allele frequency for the SNPs in the region (Figure S4). The CNV burden was then contrasted between cases and controls using Fisher’s exact test.

Principal component analysis (PCA) of the genotype data was performed using EIGENSTRAT [20], as implemented in the EIGENSOFT package (http://genepath.med.harvard.edu/°reich/Software.htm).

## Results

Overall, in the final dataset we made an average of 3.5 CNV calls per subject with a median CNV length of 76.4 kb. Of these, 60% correspond to deletions and 40% to duplications (Figure S3).

We contrasted the total CNV burden between TS cases and controls, stratified by size into four categories: <10 kb, 10–100 kb, 100–500 kb and >500 kb (Table 1). We found a statistically significant increase in the frequency of CNVs >500 kb in cases (27 or 0.15 per individual) compared to controls (15 or 0.06 per individual; *p* = 0.006). In total, 25 cases (14%) versus 15 controls (6.4%) were found to carry large CNVs, representing an excess of ∼7.6% (95% C.I. = 1.6–13.6%, one-sided Fisher’s exact test *p* = 0.006). Of the 27 large CNVs found in cases, 24 occurred in regions free of CNVs in controls. Two of the TS cases had two large CNVs each, while no control carried more than one large CNV. Since no controls were available for the CVCR samples, we evaluated the effect of population stratification by testing the correlation of CNV burden with ancestry of the samples, evaluated using PCA. The presence of large CNVs was not correlated with ancestry (*p*>0.05 for PCs 1 to 4). We also verified that OR estimates for large CNVs are consistent whether the CVCR cases are included (95% ci: 1.27–4.96) or not (95% ci: 1.08–5.95), but as expected from a reduction in sample size, when the burden analysis is restricted to Antioquia the significance decreases (one-sided Fisher’s exact test *p* = 0.16). Because cases and controls were genotyped in two batches (one batch of CVCR cases and one batch of Antioquia cases and controls), we also tested for correlation of genotyping batch with the presence of large CNVs, but found no significant effect.

**Table 1:** CNV burden in TS cases and controls.

CNV size (kb)	Count in cases	Frequency per case	Count in controls	Frequency per control	p-value
<10	10	0.06	22	0.09	NS
10–100	382	2.13	498	2.13	NS
100–500	194	1.08	300	1.28	NS
>500	27	0.15	15	0.06	0.006
Total	613	3.42	835	3.56	NS

NS = Not significant.

We next explored the potential involvement in TS of CNVs at specific genome regions, stratifying by size. We first examined the 24 (out of 27) regions with CNVs >500 Kb that were detected only in the cases. Of these, 4 did not include exons of any annotated gene. The remaining 20 mapped to 15 different genomic regions. Two of these contain genes for uncharacterized proteins with no known functions (*LOC284749* and *FLJ46357*). The remaining 18 large CNVs were located in 13 gene regions (Table S1). Of these regions, 10 presented rearrangements in a single case and some of these regions could be of potential relevance for TS (such a region on 22q11 overlapping DiGeorge’s syndrome critical region (Figure S4–43) which has been implicated in rare unusual TS cases [21], [22] and has also been found to be associated with schizophrenia [23]–[25]). Three regions showed rearrangements in more than one TS case. A ∼600 Kb region on 3q12.1 (overlapping the *COL8A1* gene) was duplicated in four cases (Table 2). Two other regions on 2p22.3 and 5q21.1 (overlapping the *BIRC6/TTC27/LTBP1* and the *SLCO4C1/SLCO6A1* genes, respectively) were duplicated in two cases each (Table 2). We also examined genome regions with CNVs <500 kb but focusing solely on those encompassing exons of the same gene in at least two TS cases but not in controls. We identified four such regions, each carrying a CNV in two patients (Table 3). The largest rearrangements (two ∼400 kb deletions) encompass exons 1–3 of the *Neurexin1* (*NRXN1*) gene on 2p16.3 (Figures S4–6 and S4–7).

**Table 2:** Chromosomal regions harbouring large (>500 kb) CNVs overlapping annotated gene exons in at least two TS cases and not in controls.

Location	CNV Type^a^	Start position^b^	End position	Size	# of markers	Gene(s)	Figure
2p22.3	Dup	32,487,194	33,186,442	699,249	145	*BIRC6,TTC27,LTBP1*	S4–4
	Dup	32,487,194	33,174,461	687,268	134	*BIRC6,TTC27,LTBP1*	S4–5
3q12.1	Dup	100,269,291	100,876,782	607,492	105	*COL8A1*	S4–9
	Dup	100,269,291	100,886,715	617,425	113	*COL8A1*	S4–10
	Dup	100,269,291	100,886,715	617,425	108	*COL8A1*	S4–11
	Dup	100,249,016	100,886,715	637,700	105	*COL8A1*	S4–12
5q21.1	Dup	101,503,405	102,033,686	530,282	66	*SLCO4C1,SLCO6A1*	S4–21
	Dup	101,532,676	102,033,686	501,011	70	*SLCO4C1,SLCO6A1*	S4–22

a Dup = duplication;

b Based on build 36 of the human genome.

**Table 3:** Regions harbouring smaller CNVs (<500 kb) overlapping gene exons in at least two TS cases but not in controls.

Location	CNV type^a^	Start position^b^	End position	Size	# of markers	Gene(s)^b^
2p16.3	Del	50,817,046	51,203,727	386,682	103	*NRXN1* c
	Del	51,022,554	51,422,546	399,993	86	*NRXN1* d
10q23.33	Del	97,352,018	97,391,986	39,969	16	*ALDH18A1*
	Del	97,353,334	97,391,986	38,653	15	*ALDH18A1*
12q24.33	Dup	131,674,763	131,772,074	97,312	20	*P2RX2,POLE*
	Dup	131,665,952	131,772,074	106,123	24	*P2RX2,POLE*
21q22.12	Dup	36,412,525	36,502,751	90,227	23	*CBR3,DOPEY2*
	Dup	36,412,525	36,479,912	67,388	15	*CBR3,DOPEY2*

a Dup = duplication; Del = deletion;

b Based on build 36 of the human genome;

c Figure S4–6;

d Figure S4–7.

We followed up the *COL8A1* and *NRXN1* findings using multiplex ligation-dependent probe amplification (MLPA; Methods S1) targeting exons 1 and 2 of *COL8A1* and exons 1 to 4 of *NRXN1* (with two additional probes 3′ and 5′ of this gene) (Table S2). We carried out MLPA in the Antioquian samples included in the SNP-based analysis for which DNA was available (92 cases and 142 controls). We validated the five SNP-based CNV calls (four on *COL8A1* and one on *NRXN1*) made on these samples (Figure S5-1). MLPA identified an additional three *COL8A1* deletions and two *NRXN1* deletions not detected in the SNP-based CNV calls (Figures S5-2 and S5-3). No CNVs in *COL8A1* or *NRXN1* were detected by MLPA in the controls. We also applied the *COL8A1* and *NRXN1* MLPA assay to an additional set of 53 TS cases from Antioquia but did not detect further rearrangements in these individuals. Aggregating the results of the SNP-based CNV calls and MLPA (Table 4), in a total of 232 cases examined we found 7 with rearrangements in *COL8A1* (all from Antioquia) and 4 in *NRXN1* (3 from Antioquia and 1 from the CVCR). None of the 234 Antioquian controls showed rearrangements in these two gene regions in the SNP-based calls or MLPA. To further support the notion that the CNVs observed here are not simply population polymorphisms, we checked the Database of Genomic Variants (DGV; http://dgvbeta.tcag.ca/dgv/app/home), a curated catalogue of human structural variation, for CNVs in the *NRXN1* and *COL8A1* gene regions. While there is a considerable number of CNVs in both regions, all of the CNVs that lie within the respective gene itself are between a few hundred bp and ∼100 kb long, and therefore significantly shorter than the variants described here. More importantly, the majority of these variants do not affect any of the exons of the respective genes, the only exception being a 100 kb deletion affecting *NRXN1* exons 7-9 (DGV Variation_2383). This variant affects a different region from the variants observed here; in addition, it was found only in one out of 540 chromosomes and is therefore also not likely to represent a common population polymorphism. Overall, the size and position of the variants identified here, both in *NRXN1* and *COL8A1*, do not show any overlap with common population polymorphism.

**Table 4:** Number of TS cases and controls with CNVs affecting *COL8A1* and *NRXN1* detected using SNP-based calls, MLPA or both.

Gene	SNP-based calls		MLPA^b^					Aggregated totals		*p*-value^a^
	Cases N = 179	Controls N = 234	Cases N = 92 (of 179)			Controls N = 142(of 234)	Additional cases N = 53^c^	Cases N = 232	ControlsN = 234	
			SNP-based calls	Validated	Additional CNVs detected					
*COL8A1*	4	0	4	4	3	0	0	7	0	0.004
*NRXN1*	2	0	1	1	2	0	0	4	0	0.03

a One-tailed Fisher’s exact test.

b MLPA was applied to a subset of the samples examined in the initial SNP-based calls.

c This set of 53 follow-up samples was not included in the initial SNP-based calls but was examined only with MLPA.

To evaluate the possibility that the *COL8A1* and *NRXN1* rearrangements detected in TS cases could represent *de-novo* mutations, we applied the MLPA assay to the parents of TS cases with rearrangements in these two gene regions. We considered only the patients for which DNA from both parents was available and confirmed relatedness in each trio. This included two cases with *COL8A1* duplications and three cases with *NRXN1* deletions (all from Antioquia). The same duplication was found in a parent in each of the two cases with *COL8A1* duplications examined, indicating that this variant was inherited. This and the observation of similar boundaries for the *COL8A1* duplications in the SNP-based CNV calls (Table 2) suggest that this variant is segregating in the Antioquian population. Deletion of *NRXN1* 5′ exons was found in the father of one of the cases with a *NRXN1* deletion (GT64.1) but not in the parents of the two other cases with this deletion, indicating a *de novo* mutation in these two trios. The father of case GT64.1 has a diagnosis of OCD, a condition that shows significant co-morbidity and may share common predisposing factors with TS (interestingly, the paternal grand-father is reported to have suffered from OCD; however, his CNV type is unknown). One of the two *de novo NRXN1* deletions identified occurred in a proband that had no family history of TS (case GT5.1, Figure S5-2a). The second case with a *de novo NRXN1* deletion (GT34.1, Figure S5-2b) had a history of TS/OCD on the paternal side of his family.

## Discussion

Our results provide statistically significant evidence of a high burden of large CNVs (>500kb) in TS, thereby supporting the proposal for an involvement of rare CNVs in various neurodevelopmental disorders, including TS, and their possible aetiological overlap [12], [13], [26]–[28]. We also find suggestive evidence for the involvement of rearrangements specifically affecting the *NRXN1* and *COL8A1* genes. In the aggregated data (Table 4) we find a nominally significant association of *COL8A1* and *NRXN1* rearrangements with TS (p-values of 0.004 and 0.03 respectively). Due to the limited sample size, these p-values would not reach significance accounting for multiple testing. Data from the Database of Genomic Variants further supported the notion that the variants observed here are not part of the spectrum of common population polymorphisms. When considering the trio data, the lack of a straightforward co-segregation between the structural variants observed in our study and the TS phenotype implies the involvement of further predisposing loci in the aetiology of TS; however, this is not unexpected for such a phenotypically and genetically complex condition and does not conflict with a role for *NRXN1* or *COL8A1* in TS predisposition. Overall, our results strongly warrant further investigation of these two genes in TS.

The importance of *NRXN1* in mediating cell-cell interactions in the central nervous system, as well as its confirmed involvement in other neurodevelopmental disorders, make this gene an excellent candidate gene for TS [12], [29], [30]. Our results are consistent with those of a previous study reporting deletions affecting *NRXN1* exons 1–3 in TS, the same exons found to be deleted in our study [12]. The fact that two of the three *NRXN1* rearrangements, for which inheritance status could be confirmed, were found to be *de novo* events, is in line with recent findings stressing a role for *de novo* mutations in neurodevelopmental disease. The potential involvement of *COL8A1* in TS is intriguing. A growing body of evidence suggests that collagen subunits are involved in neural development, influencing processes such as axonal guidance, synaptogenesis and Schwann cell differentiation [31], [32]. *COL8A1* has also been found to be up-regulated during repair processes in the mouse brain [32]. Interestingly, the top signal in the recent GWAS of TS [8] also implicated a collagen gene (*COL27A1)*.

In conclusion, our results are consistent with the view that TS is genetically a highly heterogeneous disorder, in which rare variants, including *de-novo* mutations, could underlie a substantial fraction of cases. Recently, Cooper et al (2011) conducted a large-scale study to investigate the role of CNVs in ∼15,000 children with intellectual disability and estimated that ∼14.2% are due to CNVs >400 kb. Similarly, the 7.6% excess of large CNVs in TS patients observed here could be taken as a rough estimate of the proportion of cases that might be caused by CNVs. The analysis of larger TS study samples should enable a more definite assessment of the role of large rearrangements at specific gene regions in this disorder. More extensive surveys of parent-TS offspring trios are also required to estimate the proportion of cases that could be due to highly penetrant *de-novo* mutations. Finally, sequencing studies should allow a full assessment of the role of rare variants in the aetiology of TS.

## Supporting Information

Figure S1 No significant correlation was observed between PCs 1–4 and presence of large CNVs. Left panel: PCA1 versus PCA2. Right panel: PCA3 versus PCA4.
(PDF)

Figure S2 Samples with NumCNV>30 or LRR_SD>0.24 were excluded from subsequent analyses.
(PDF)

Figure S3 The 413 DNA samples that passed QC yielded an average of 14.47 CNV calls per subject. On applying call-level filtering criteria to these calls, an average of 3.50 CNV calls per subject (spanning 10 to 522 SNPs) were obtained. Deletions (865/1448) were more frequently observed compared to duplications (583/1448). Deletions were observed more frequently in the small CNV category while duplications were observed more frequently in the large CNV category (Figure S3).
(PDF)

Figure S4 (1–44): Sample ID, population origin and case/control status are shown as figure heading. LogR ratio and B allele frequency are shown in the top and bottom panels, respectively. CNV boundaries are indicated by red dotted lines. Human RefSeq genes are shown below each panel (vertical lines indicating exons). Genomic position (in Mb) based on the hg18 human genome sequence.
(PDF)

Figure S5 CNV calls using Multiplex Ligation-dependent Probe Amplification (MLPA). Figure S5-1: Validation of the SNP-based CNV calls in *COL8A1* and *NRXN1* by MLPA. Top panel: heterozygous duplication in *COL8A1* (exons 1 and 2). Representative MLPA data and MLPA target probes for *COL8A1* are shown. Bottom panel: Detection of a heterozygous deletion in *NRNX1* (exons 1, 2, 3). MLPA target probes for *NRXN1* are shown, the unlabelled target regions are probes located either on chromosome 2 but outside the deleted region or on other chromosomes (Table S2). Patient MLPA traces are in red, overlaid upon the normal control MLPA traces in black. Arrows point to the deleted/duplicated probes. Figure S5-2: Detection of *de novo* deletions in *NRNX1* (exons 2 and 3) in TS cases. A, trio 5. B, trio 34. Patient MLPA traces are in red overlaid upon the normal control MLPA traces in black. The parents’ traces are in blue, overlaid upon normal controls in black. Arrows point to the MLPA probes in *NRXN1.* Figure S5-3: Two additional TS cases (GT5.1 and GT34.1) with deletions involving either exon 1, 2 or 3 of *NRXN1* detected by MLPA. Representative MLPA data are shown. Patient traces are in red, overlaid upon the control traces in black. Arrows point to the MLPA probes in *NRXN1*. Figure S5-4: Three additional TS cases (GT7.1, GT29.1 and GT114.1) with deletion of exon 2 of *COL8A1* detected by MLPA. Representative MLPA data are shown. Patient traces are in red, overlaid upon the control traces in black. Arrows point to the MLPA probes in *COL8A1*.
(PDF)

Figure S6 (1 to 5): Sample ID, population origin and case/control status are shown as figure heading. LogR ratio and B allele frequency are shown in the top and bottom panels, respectively. CNV boundaries are indicated by red dotted lines. The structure of *NRXN1* (Figures S6-1 and S6-2) or *COL8A1* (Figures S6-3 to S6-5) is shown below each panel with exons shown as vertical lines. Genomic position (in Mb) provided make use of the hg18 human genome sequence as reference.
(PDF)

Table S1 Chromosomal regions harbouring large (>500 kb) CNVs overlapping annotated gene exons in TS cases but not in controls. ^a^Dup = duplication; ^b^ According to build 36 of the human genome.
(DOC)

Table S2 Target probes used in the MLPA assay.
(DOC)

Methods S1 CNV Quality Control and CNV validation by Multiplex ligation-dependent probe amplification (MLPA).
(DOCX)
