# Supplementary figures and images for: CNV Analysis in Tourette Syndrome Implicates Large Genomic Rearrangements in COL8A1 and NRXN1
Source: PLoS One. 2013 Mar 22;8(3):e59061. doi: 10.1371/journal.pone.0059061 (PMC3606459; doi:10.1371/journal.pone.0059061)

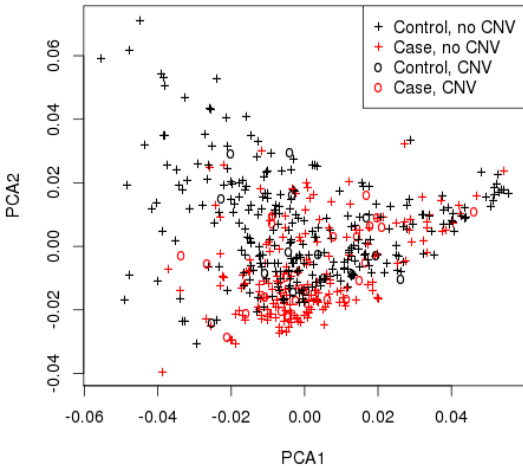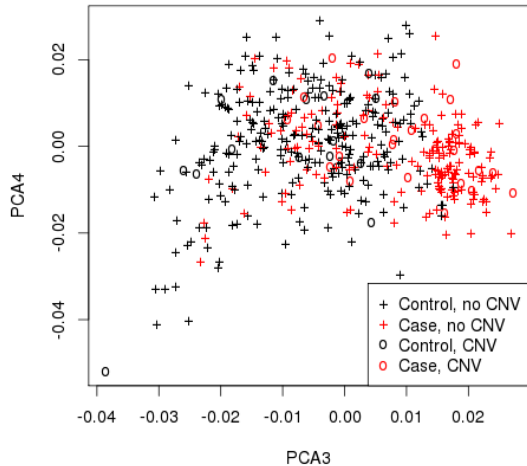

Supplement: Figure S1 — No significant correlation was observed between PCs 1–4 and presence of large CNVs. Left panel: PCA1 versus PCA2. Right panel: PCA3 versus PCA4. (PDF) [file pone.0059061.s001.pdf]

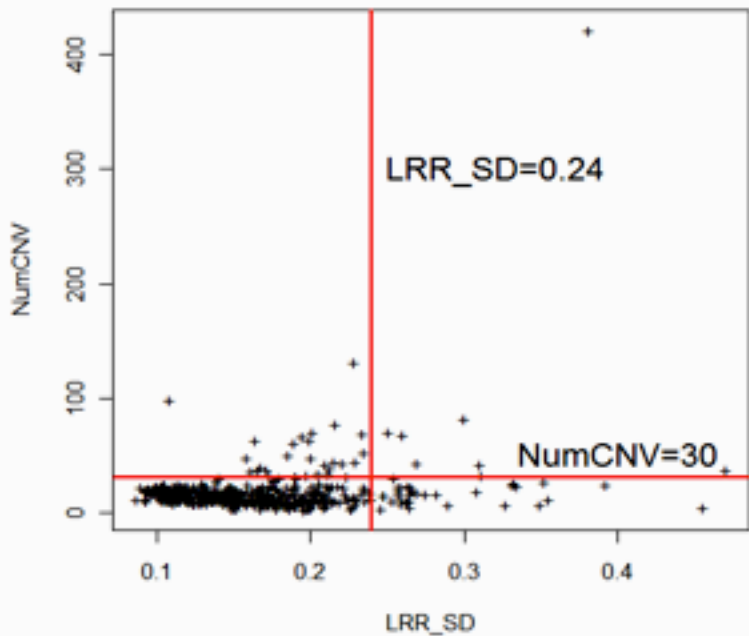

Supplement: Figure S2 — Samples with NumCNV>30 or LRR_SD>0.24 were excluded from subsequent analyses. (PDF) [file pone.0059061.s002.pdf]

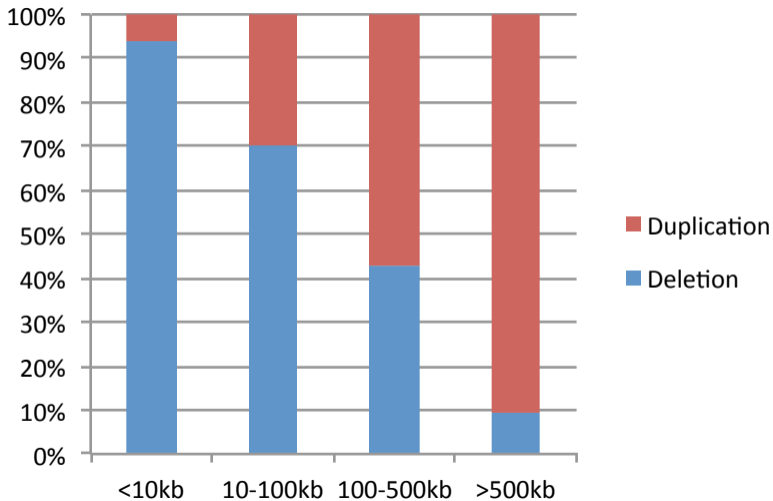

Supplement: Figure S3 — The 413 DNA samples that passed QC yielded an average of 14.47 CNV calls per subject. On applying call-level filtering criteria to these calls, an average of 3.50 CNV calls per subject (spanning 10 to 522 SNPs) were obtained. Deletions (865/1448) were more frequently observed compared to duplications (583/1448). Deletions were observed more frequently in the small CNV category while duplications were observed more frequently in the large CNV category (Figure S3). (PDF) [file pone.0059061.s003.pdf]
