# Supplementary material for: CNV Analysis in Tourette Syndrome Implicates Large Genomic Rearrangements in COL8A1 and NRXN1
Source: PLoS One. 2013 Mar 22;8(3):e59061. doi: 10.1371/journal.pone.0059061 (PMC3606459; doi:10.1371/journal.pone.0059061)

Figure S4−1, sample: 4393489295_R02C02, Internal ID:NA, COSTA RICA, case, chr1:18022097−18576259

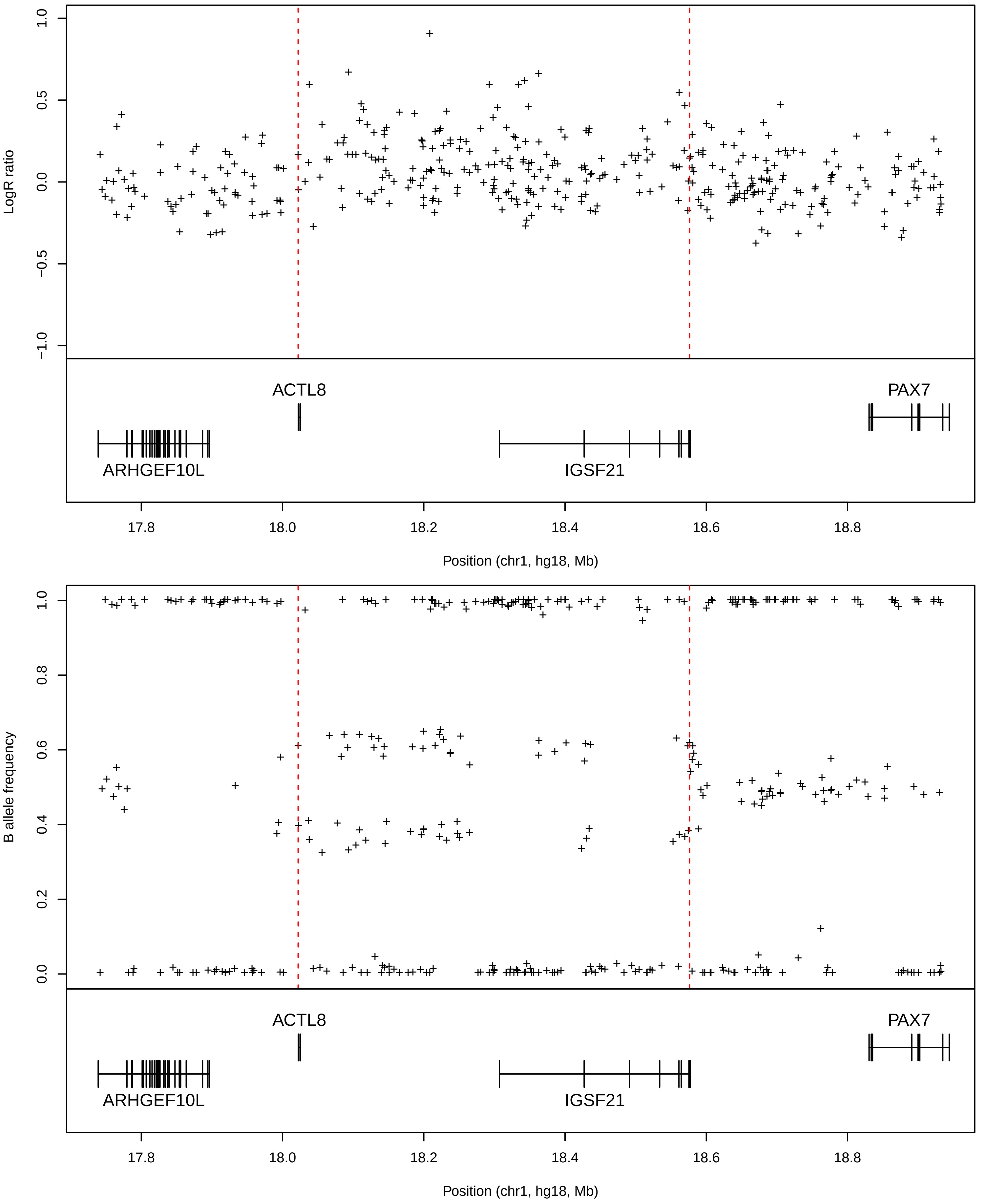

Figure S4−2, sample: 4408206477_R02C01, Internal ID:NA, COSTA RICA, case, chr1:80933925−81444601

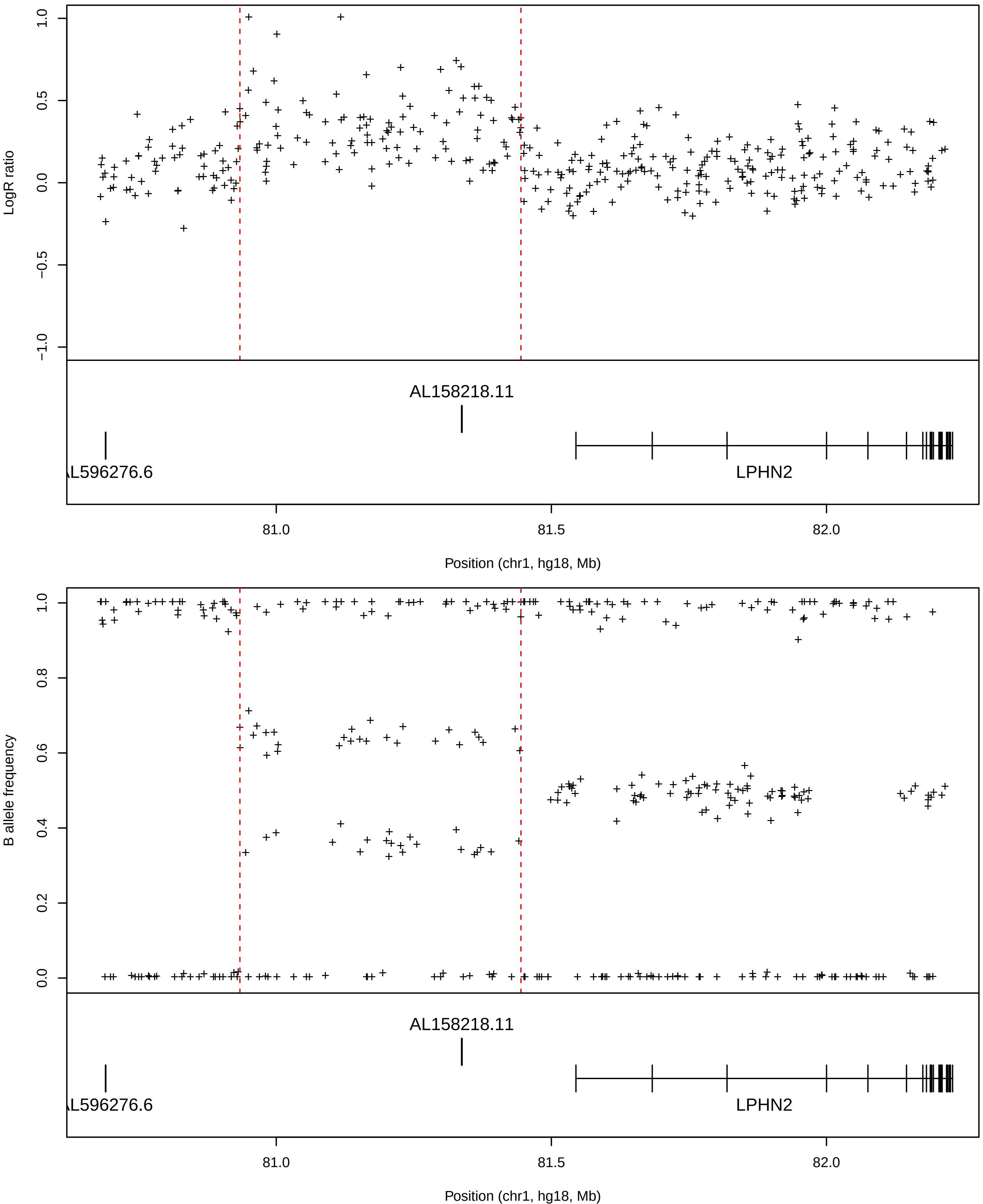

Figure S4−3, sample: 4343211370_R01C02, Internal ID:NA, COSTA RICA, case, chr2:333588−1004837

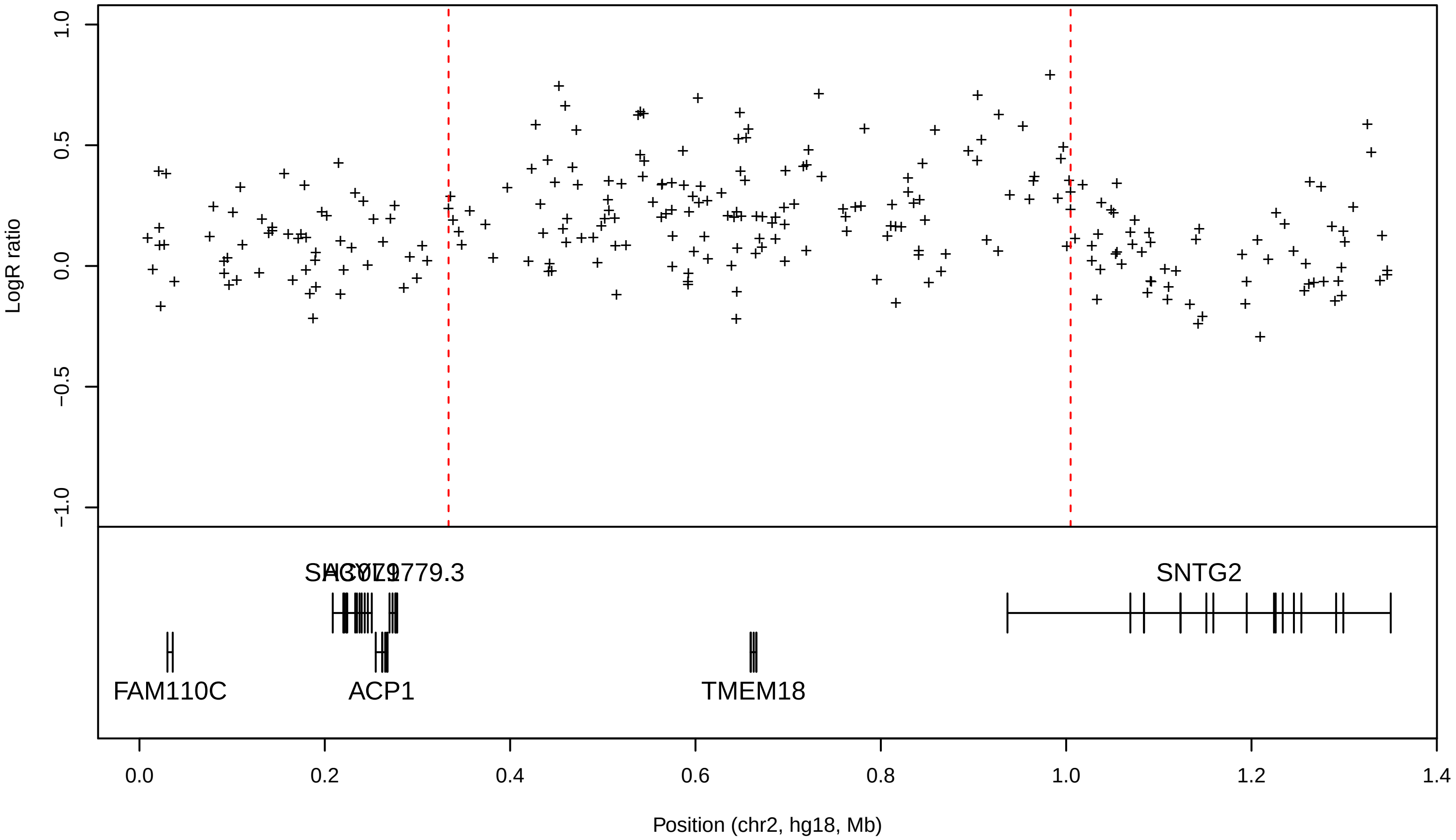

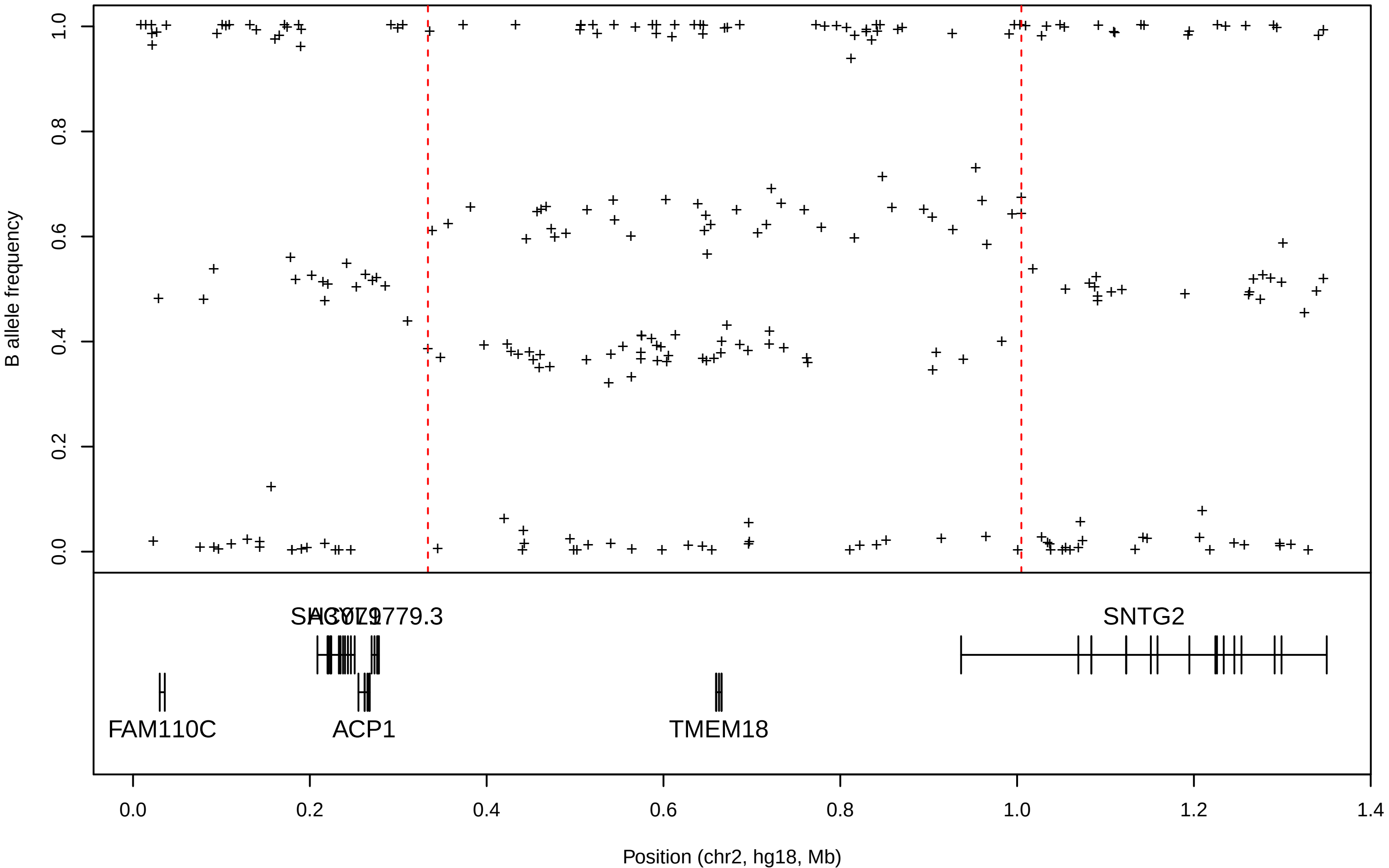

Figure S4−4, sample: 4378641751_R02C01, Internal ID:NA, COSTA RICA, case, chr2:32487194−33174461

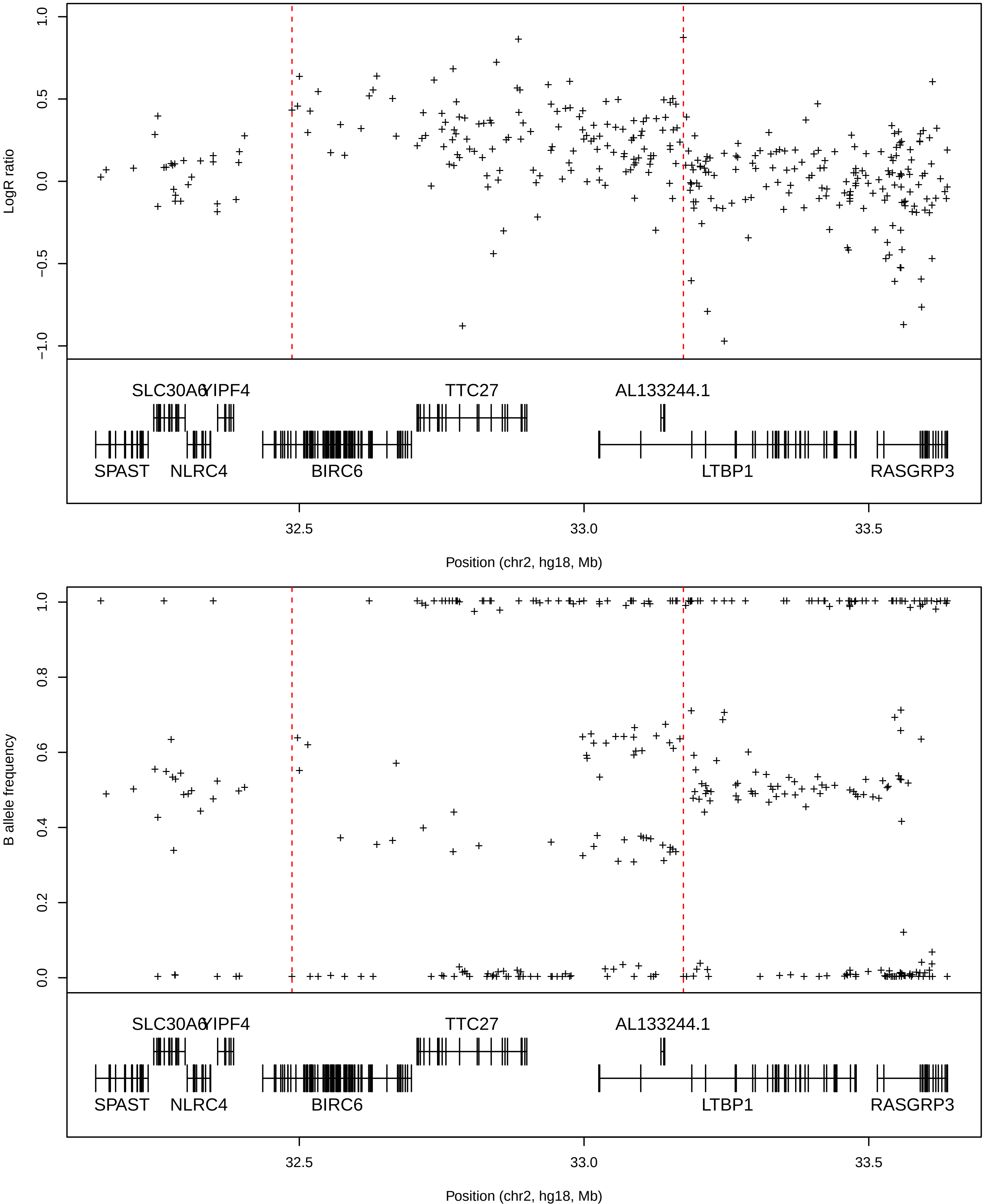

Figure S4−5, sample: 4381234508_R01C02, Internal ID:38.1, COLOMBIA, case, chr2:32487194−33186442

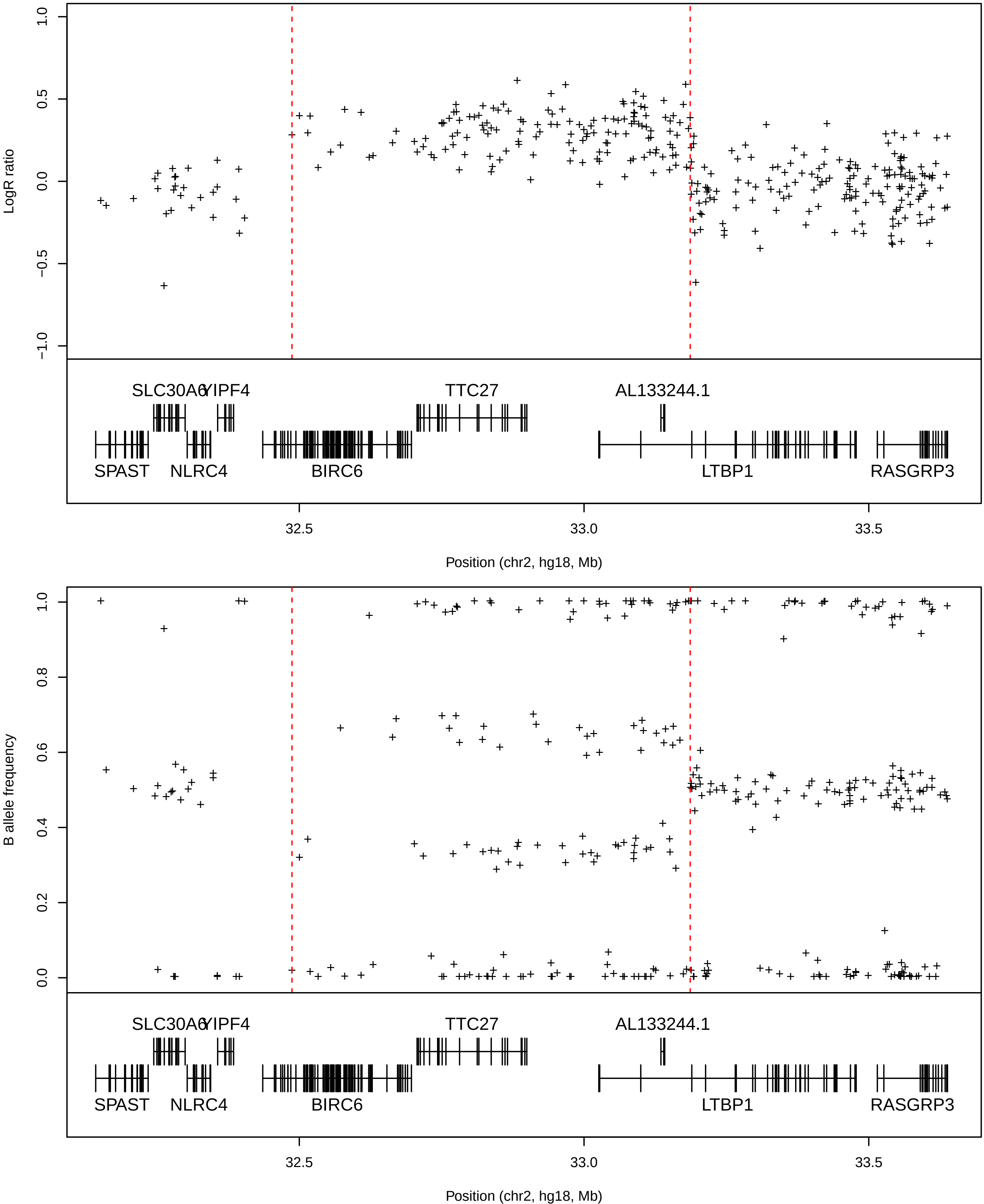

Figure S4−6, sample: 4408206324_R01C02, Internal ID:NA, COSTA RICA, case, chr2:50817046−51203727

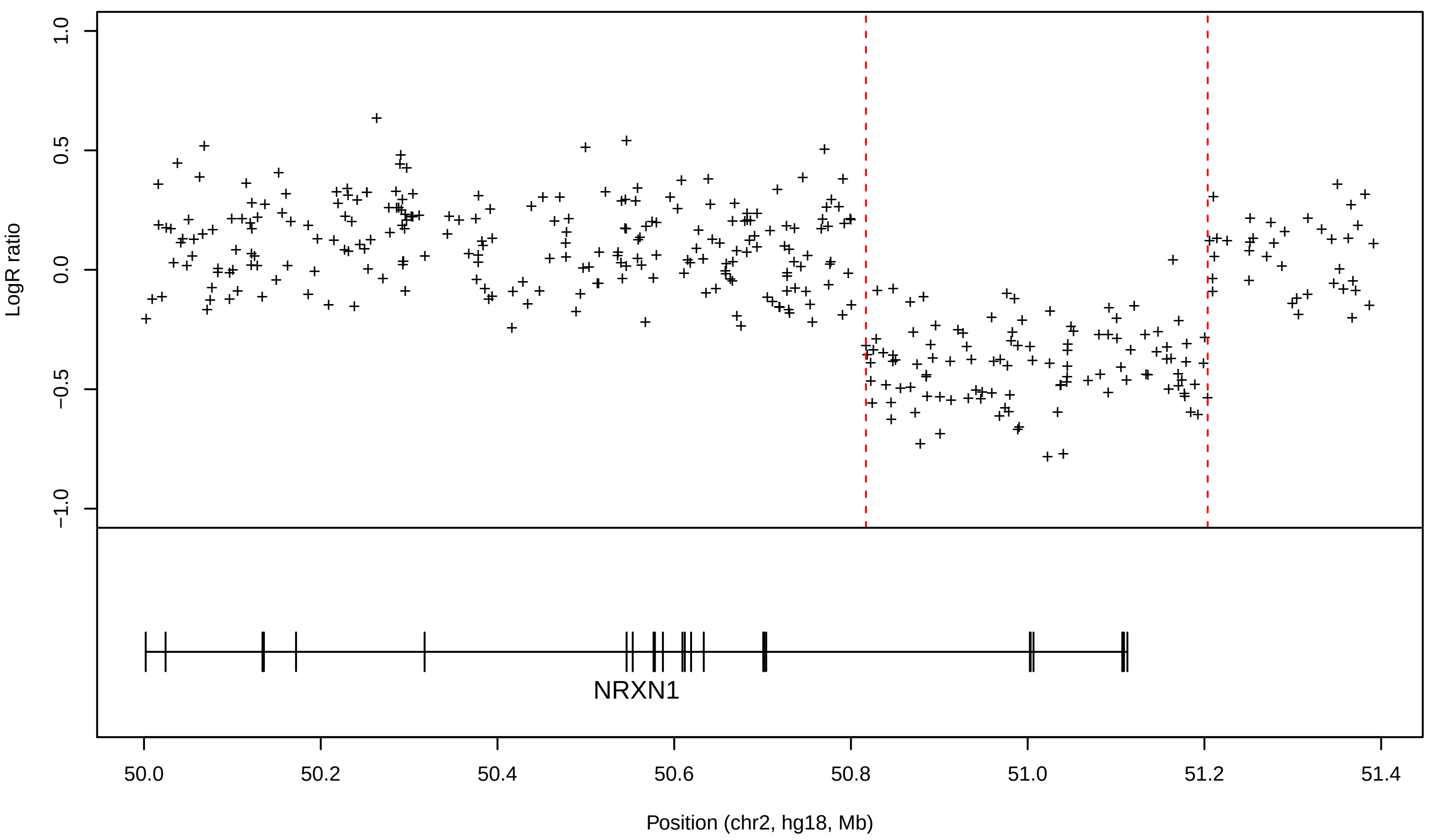

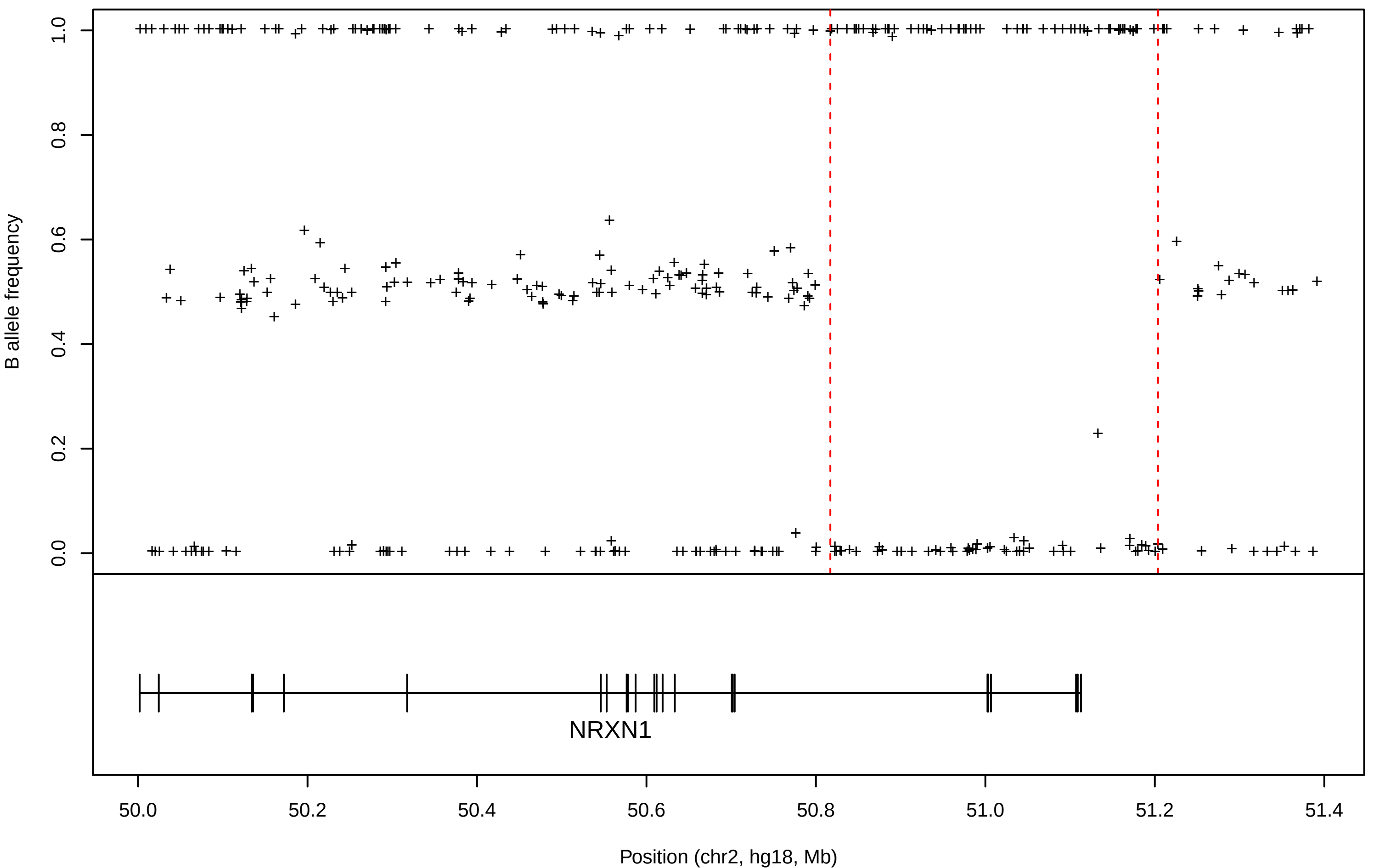

Figure S4−7, sample: 4506295058_R01C01, Internal ID:64.1, COLOMBIA, case, chr2:51022554−51422546

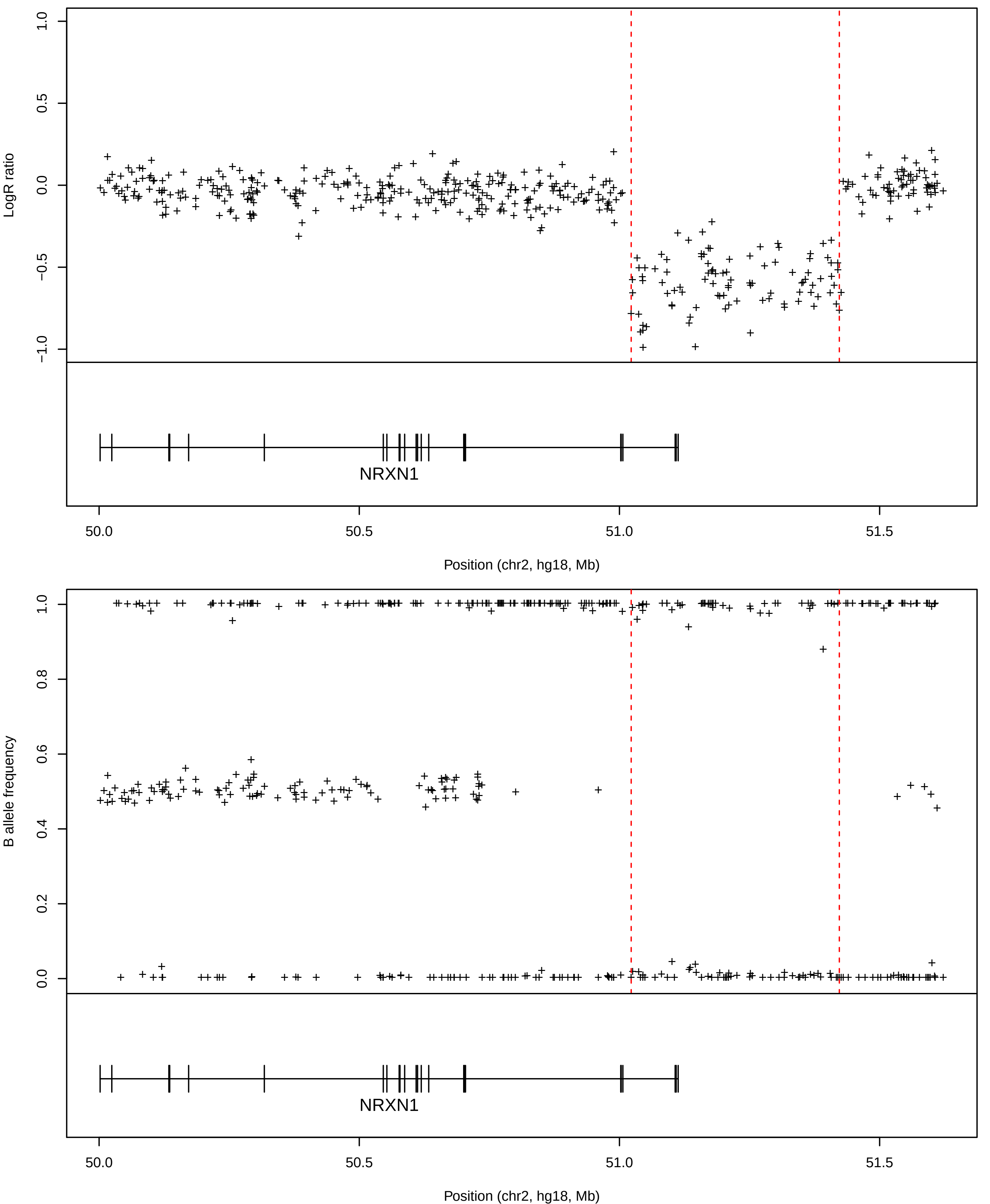

Figure S4−8, sample: 4378959456_R01C02, Internal ID:NA, COSTA RICA, case, chr3:852235−1403635

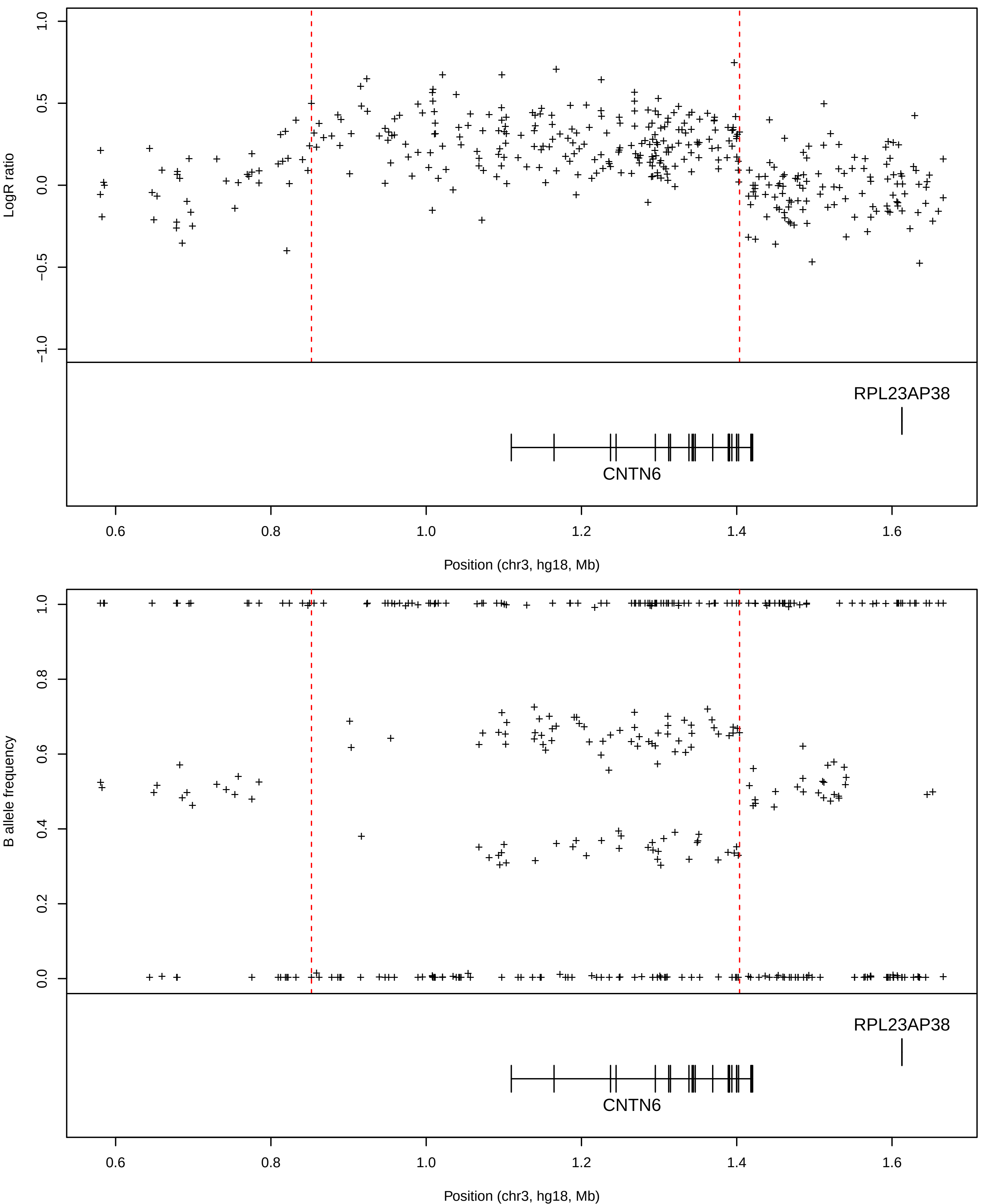

Figure S4−9, sample: 4506287122_R01C02, Internal ID:10.1, COLOMBIA, case, chr3:100249016−100886715

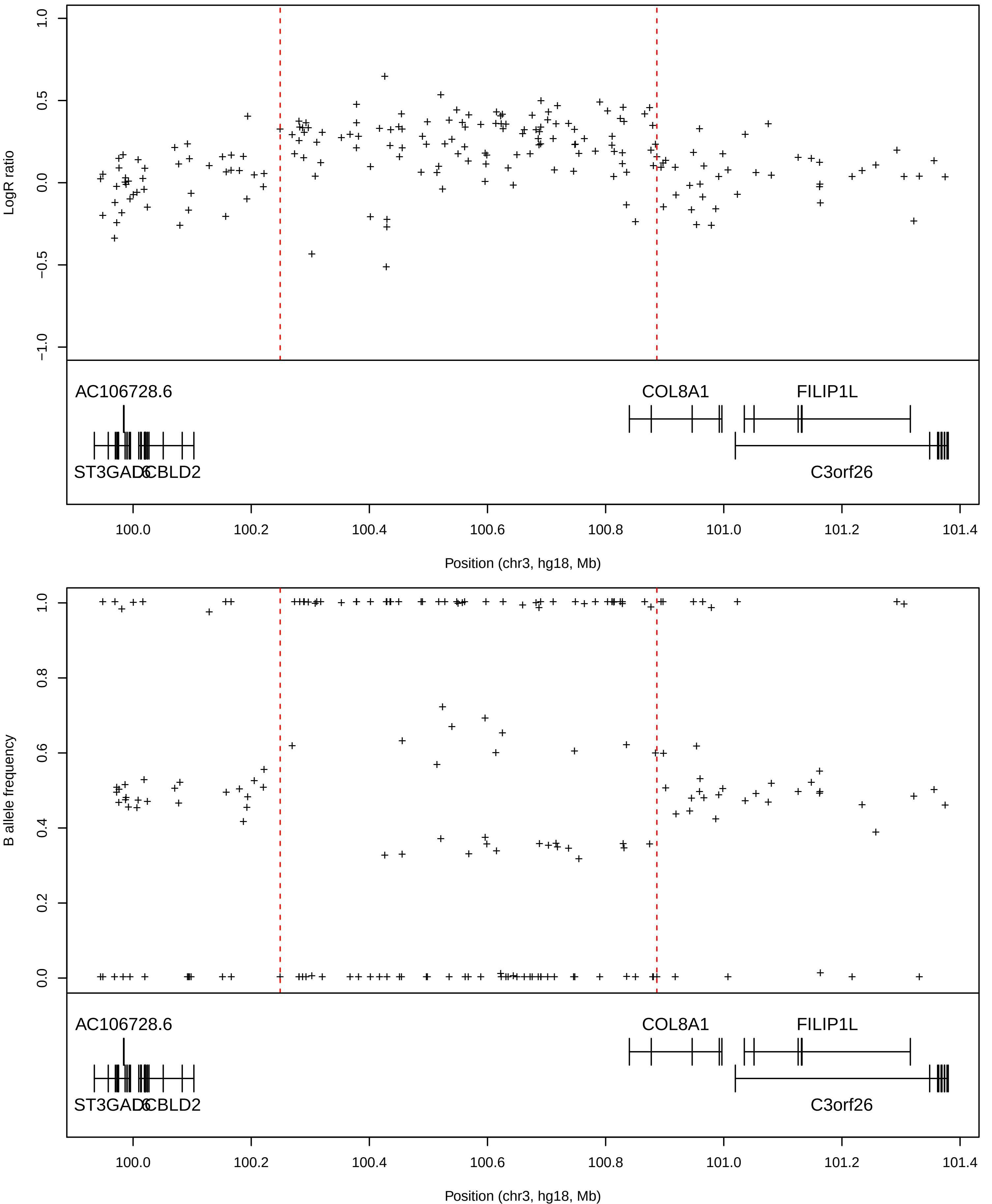

Figure S4−10, sample: 4381234052_R02C01, Internal ID:41.1, COLOMBIA, case, chr3:100269291−100876782

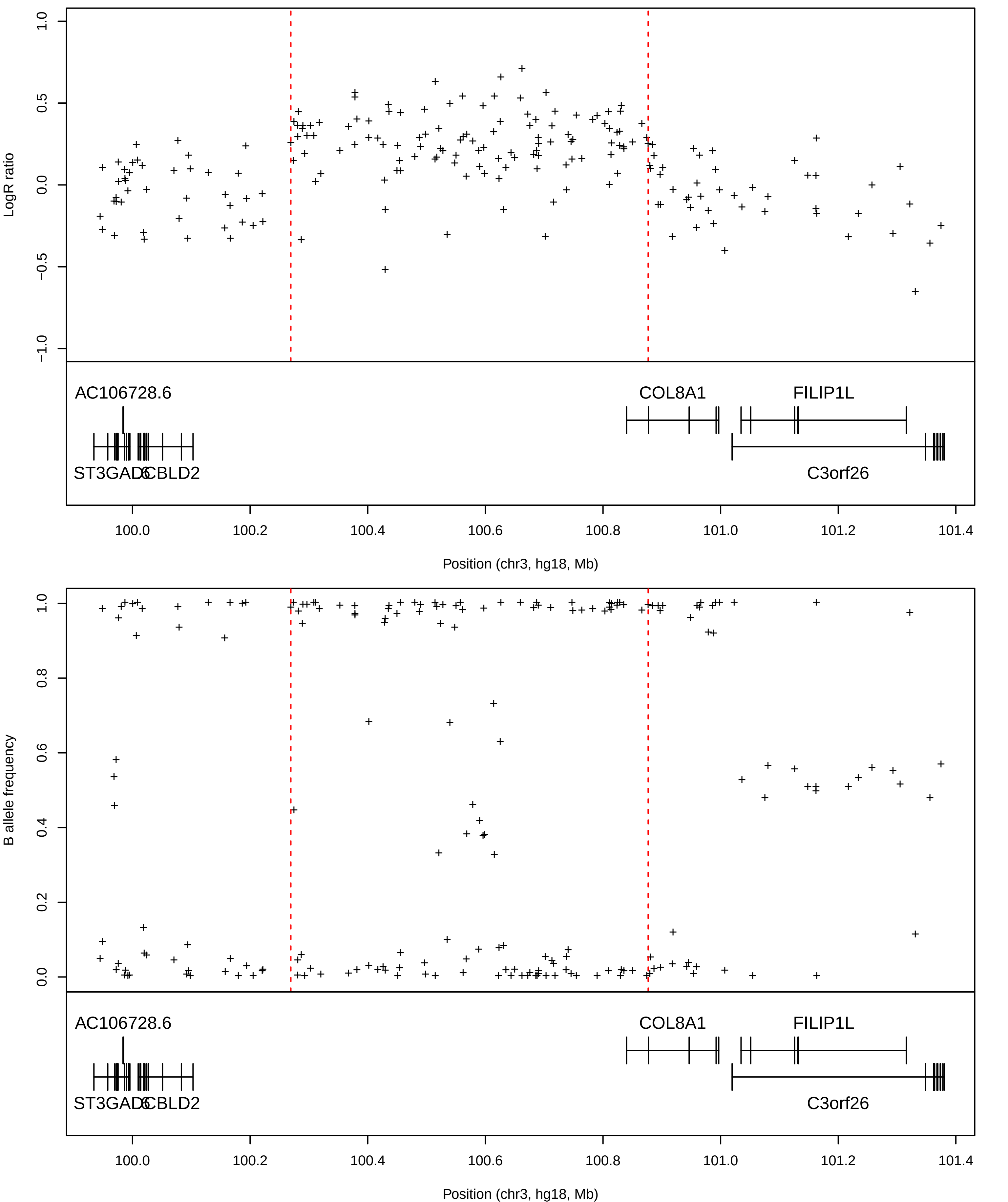

Figure S4−11, sample: 4506279006_R02C02, Internal ID:51.1, COLOMBIA, case, chr3:100269291−100886715

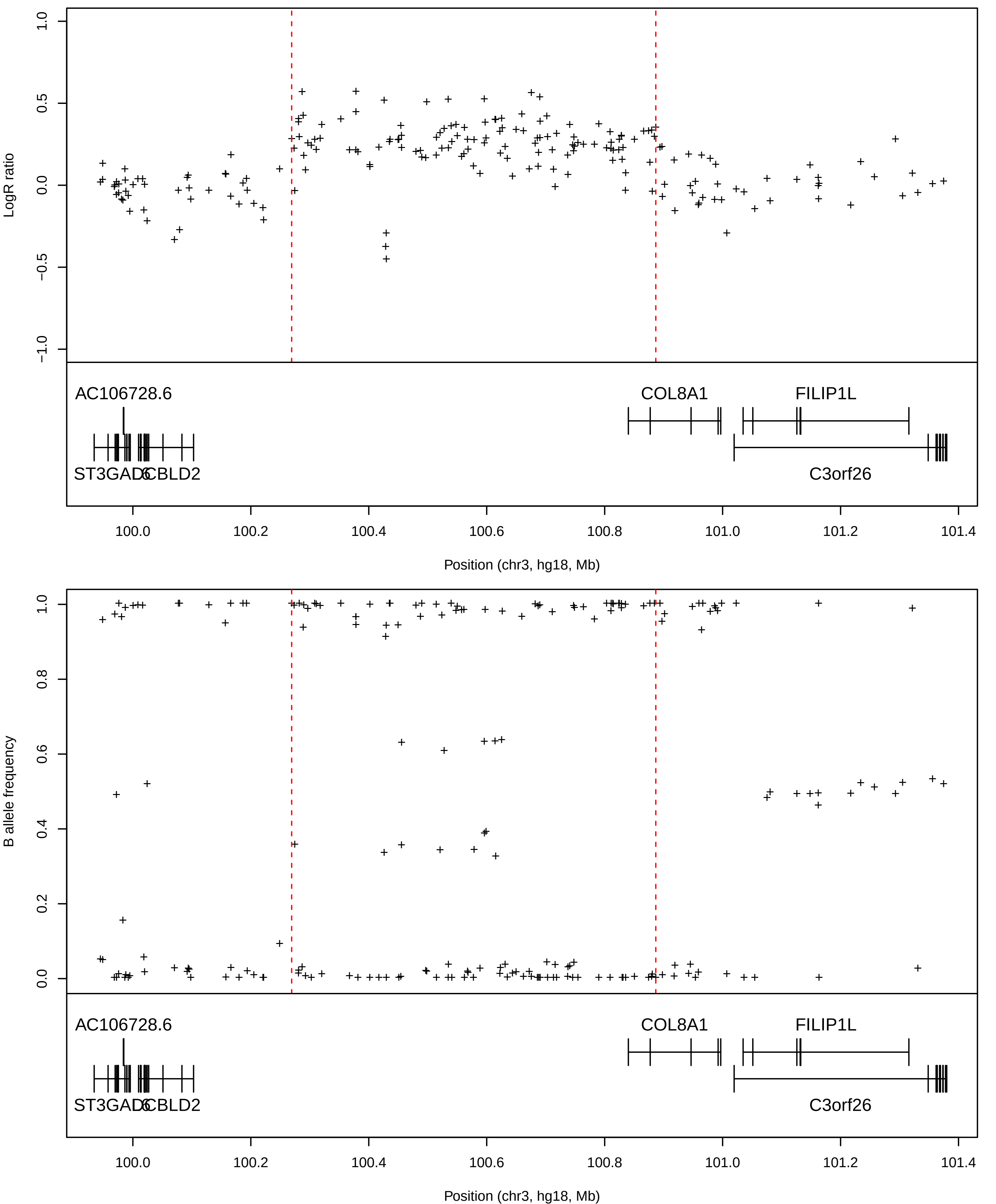

Figure S4−12, sample: 4506279152_R02C02, Internal ID:112.1, COLOMBIA, case, chr3:100269291−100886715

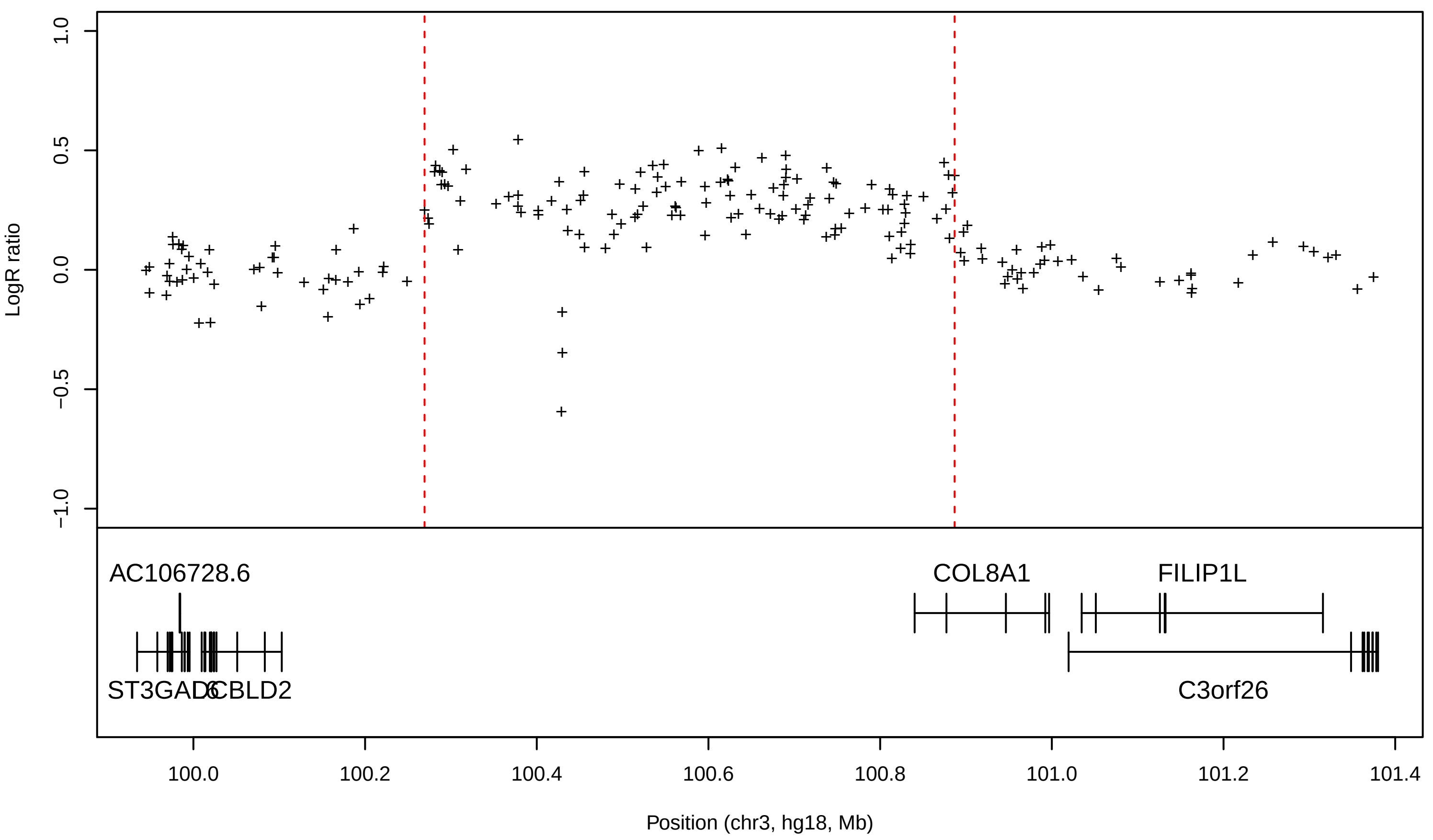

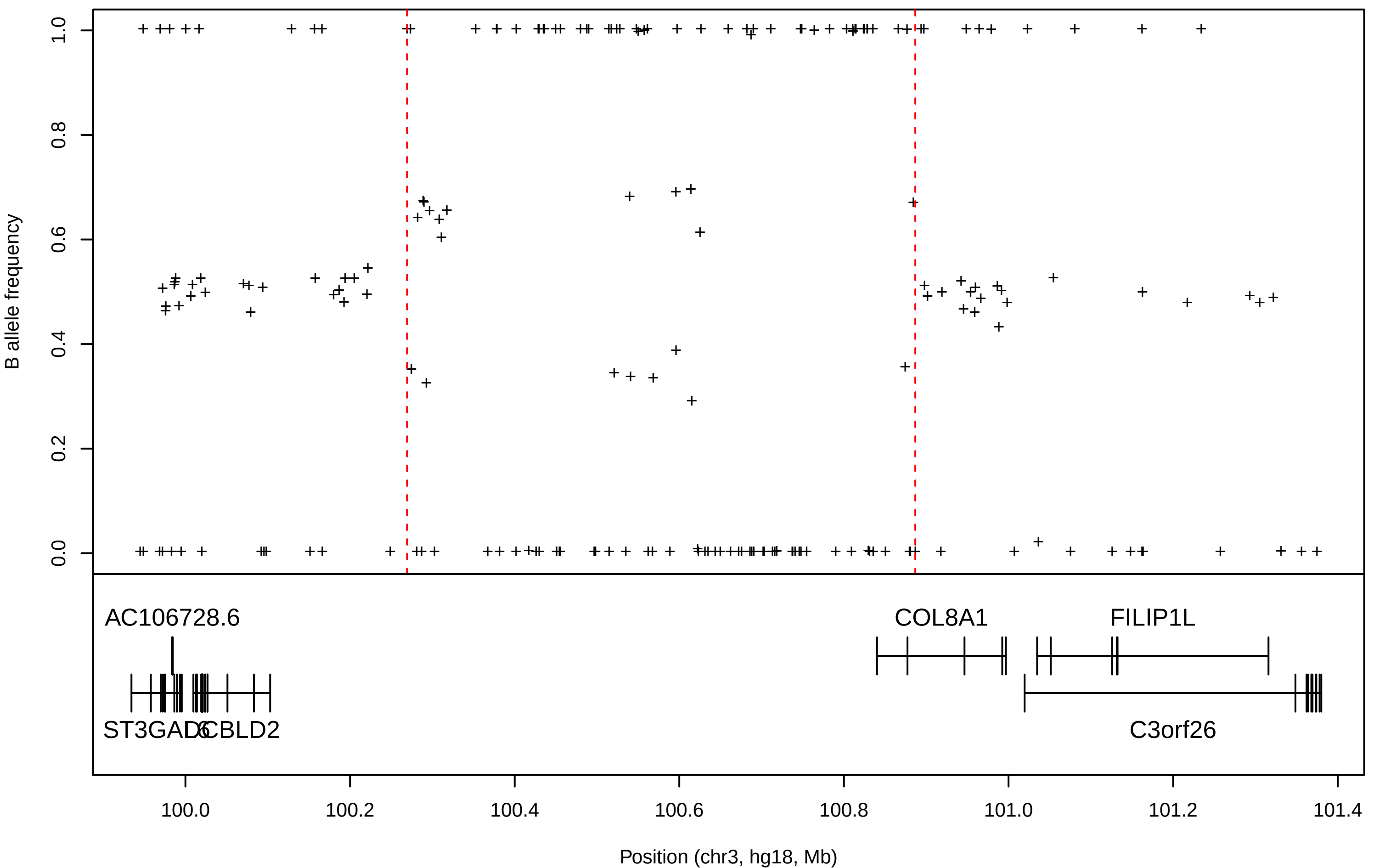

Figure S4−13, sample: 4506279048_R01C01, Internal ID:4.1, COLOMBIA, case, chr3:100280863−100803397

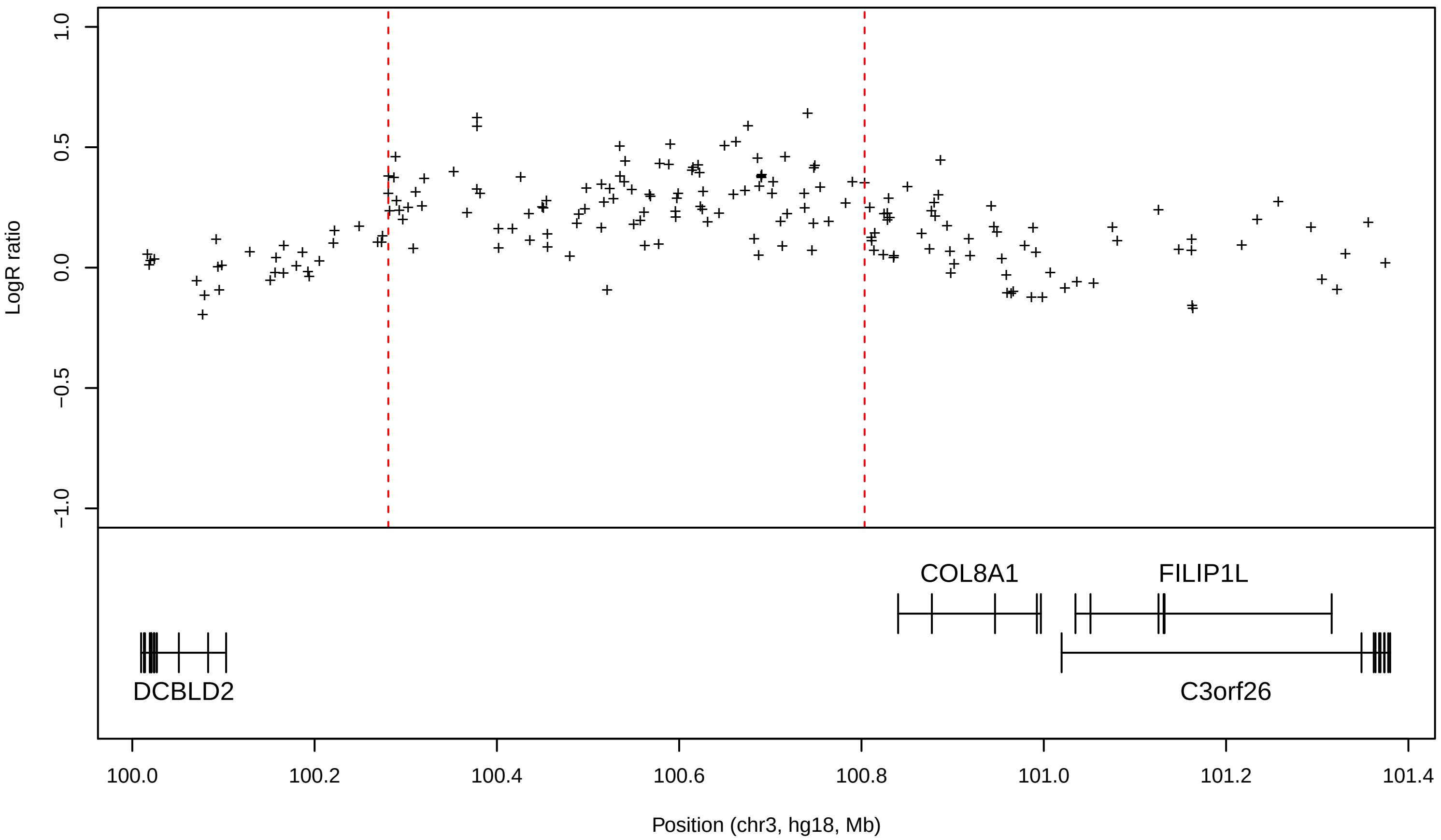

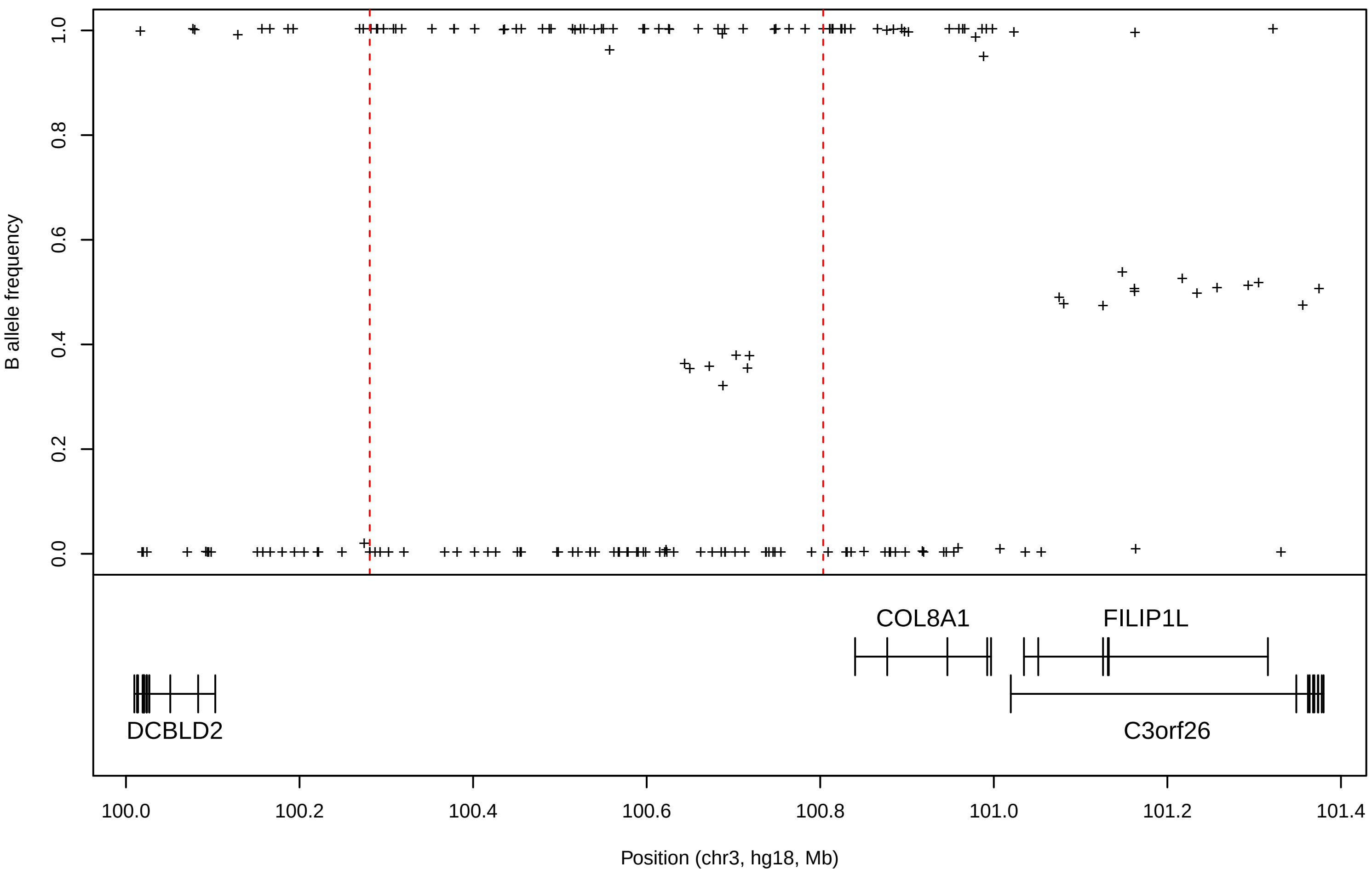

Figure S4−14, sample: 4494431075_R01C02, Internal ID:NA, COSTA RICA, case, chr4:16443738−17281982

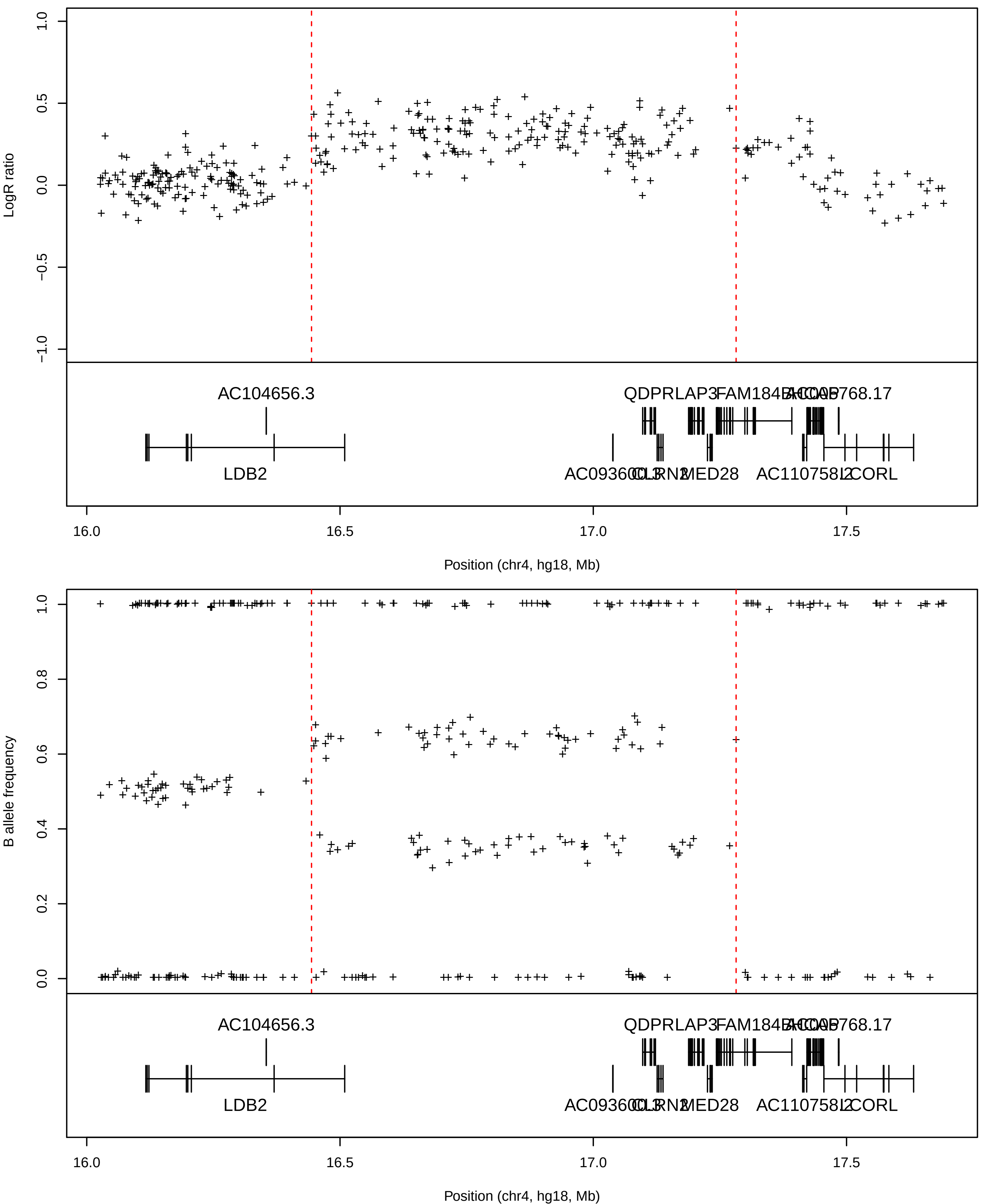

Figure S4−15, sample: 4515503168_R01C01, Internal ID:C209, COLOMBIA, control, chr4:60141415−61036349

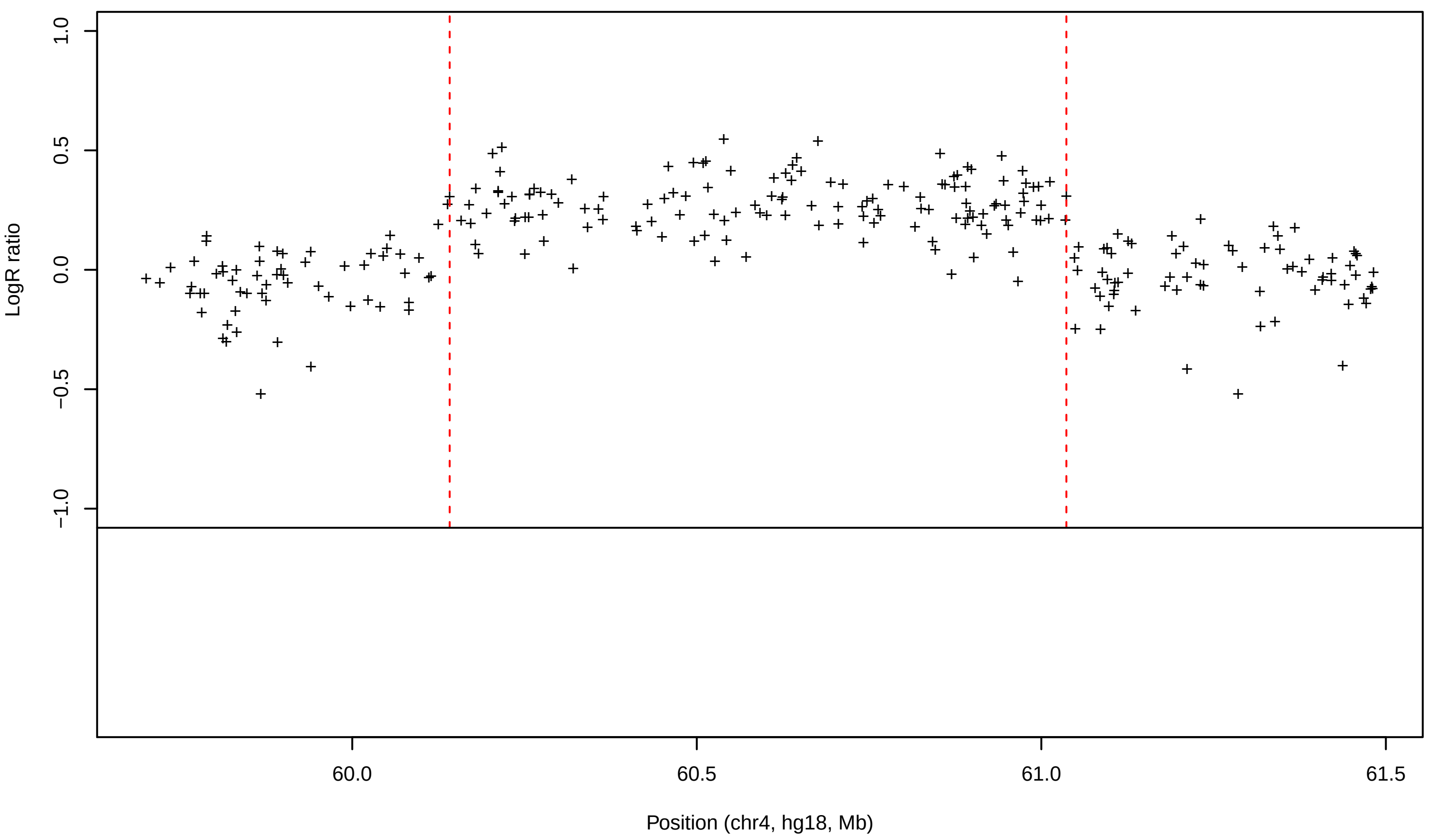

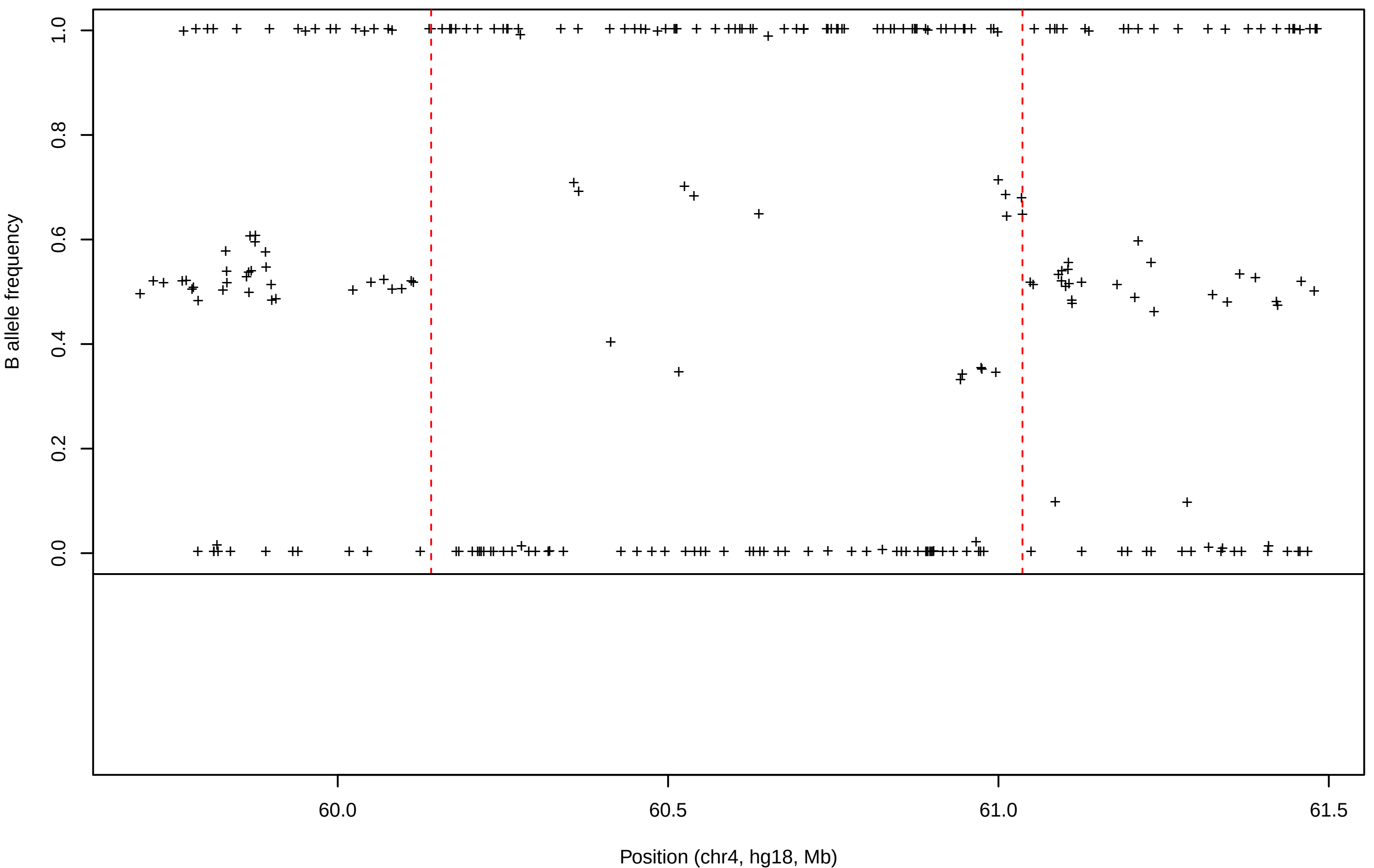

Figure S4−16, sample: 4506287107_R02C01, Internal ID:C433, COLOMBIA, control, chr4:92715486−93380874

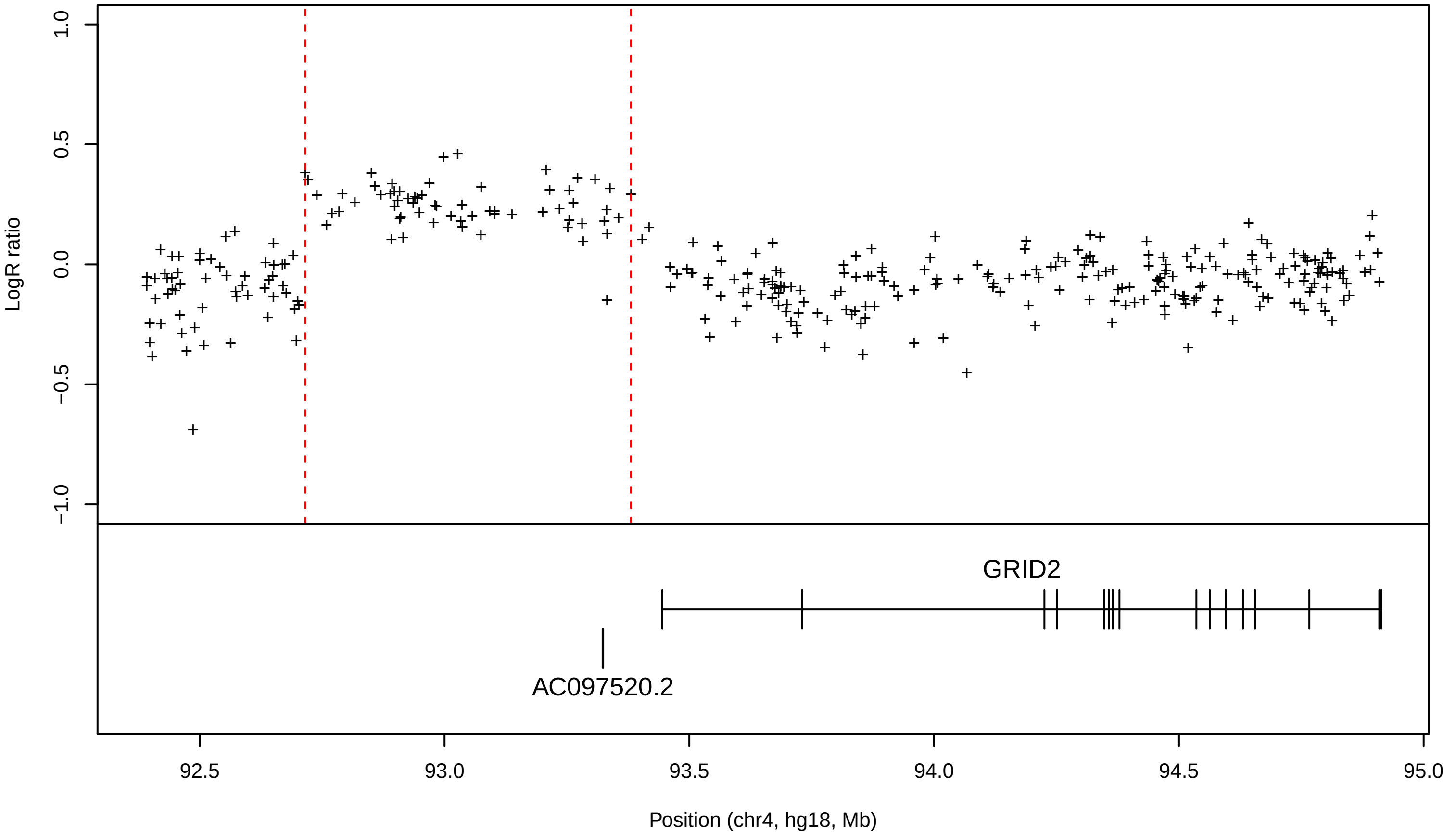

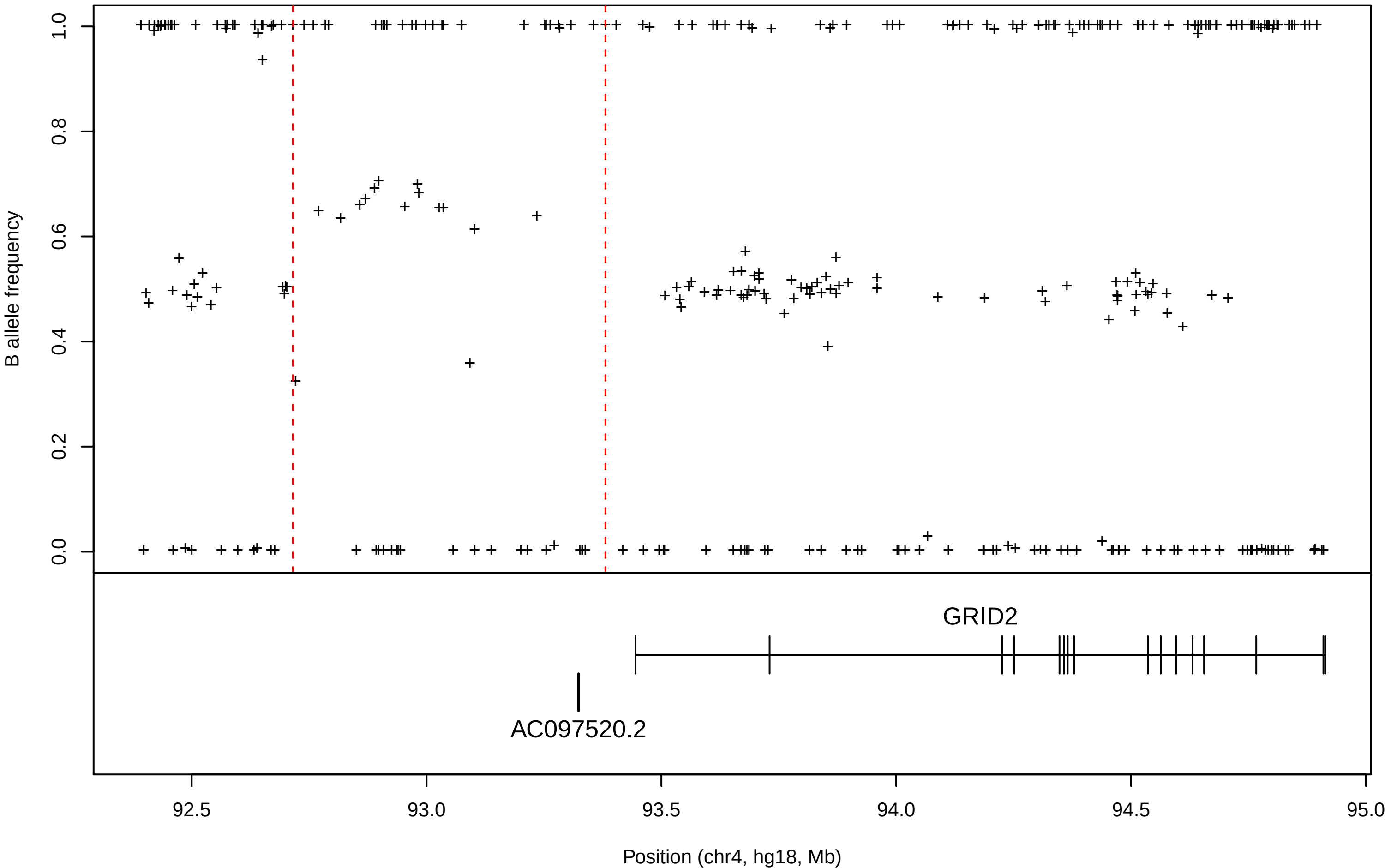

Figure S4−17, sample: 4366261658_R01C02, Internal ID:CP−054−3, COLOMBIA, control, chr4:92816858−93409221

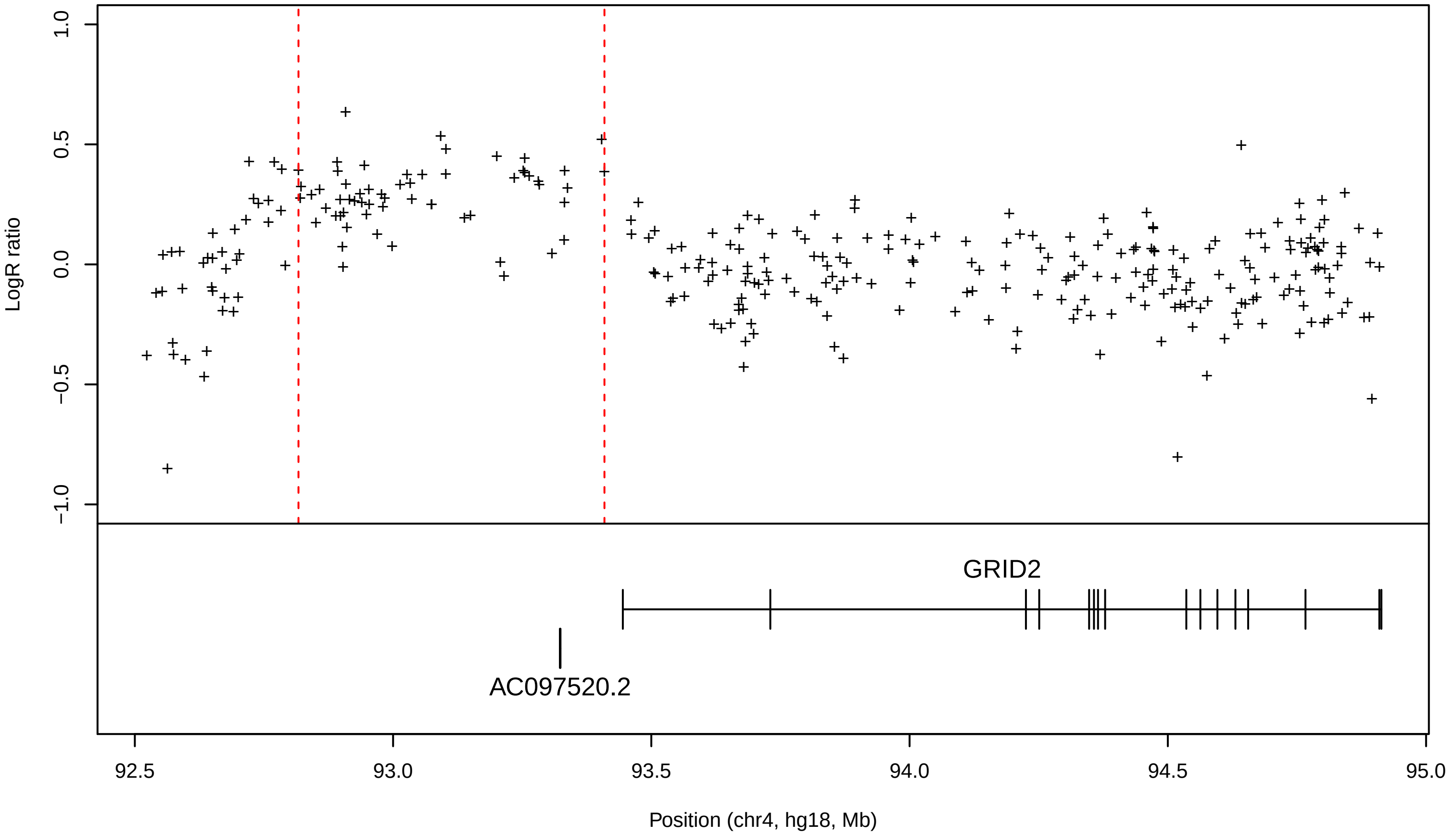

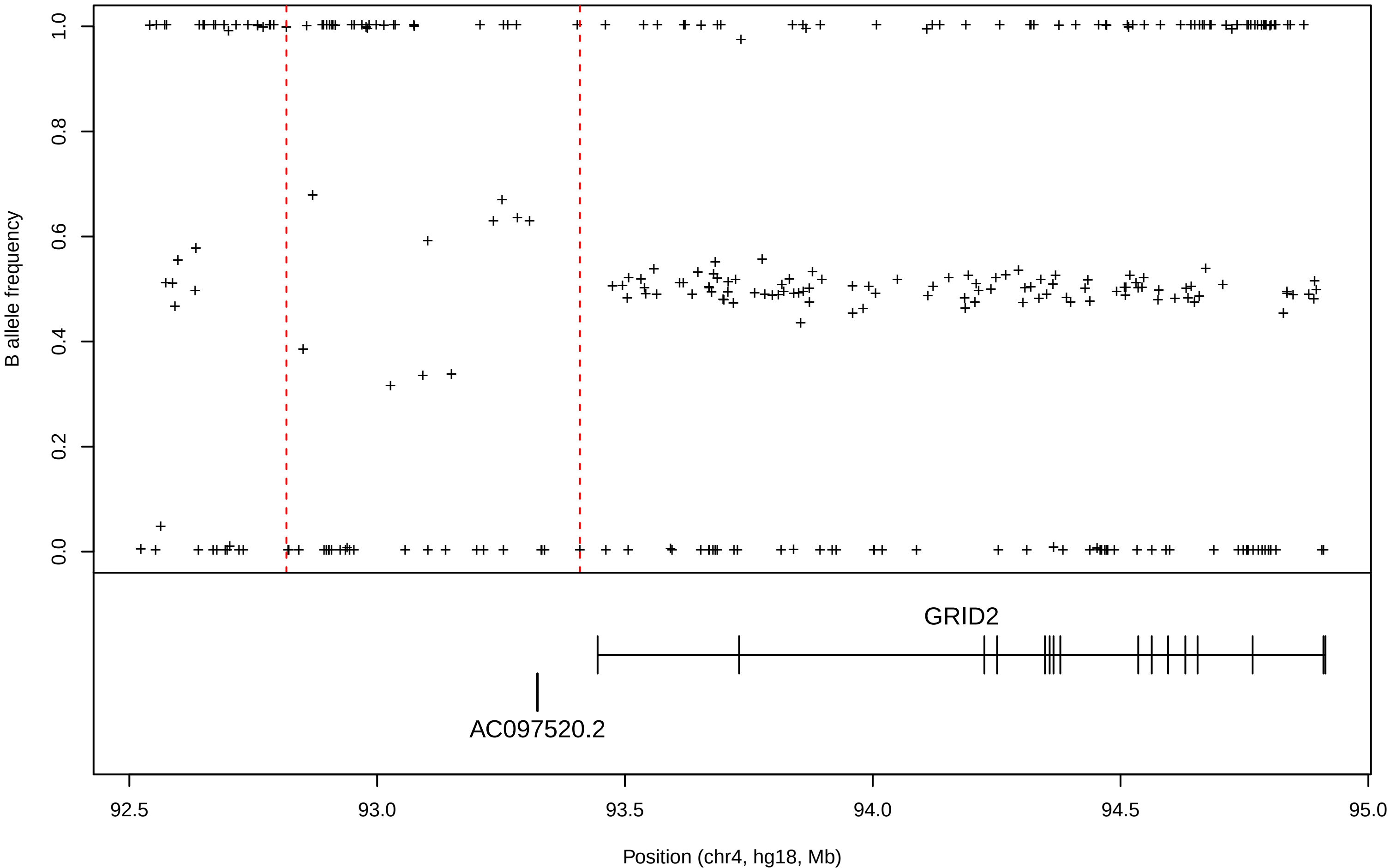

Figure S4−18, sample: 4408206718_R01C02, Internal ID:NA, COSTA RICA, case, chr4:108061142−108572634

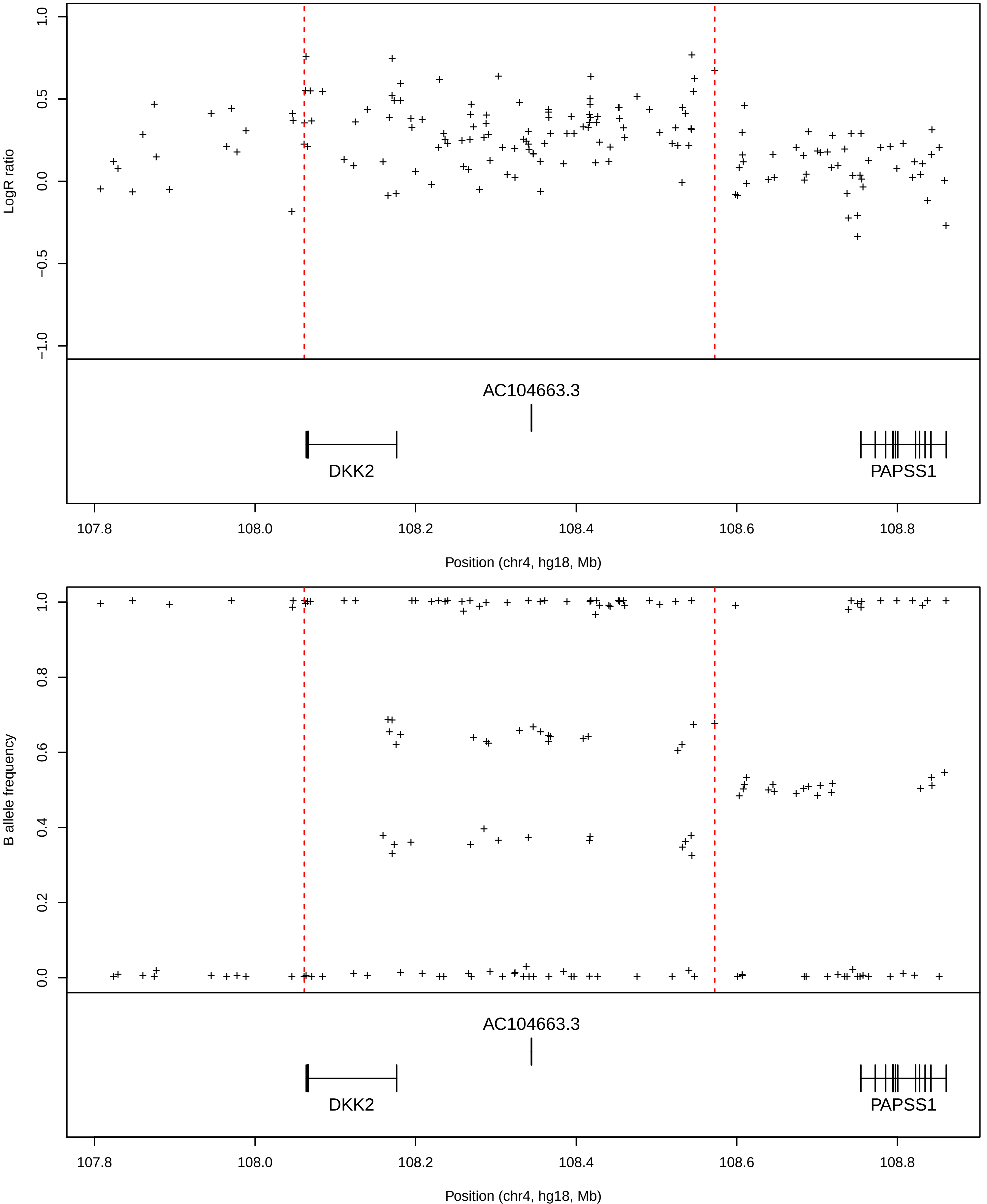

Figure S4−19, sample: 4506252049_R02C01, Internal ID:C295, COLOMBIA, control, chr5:2189570−2739712

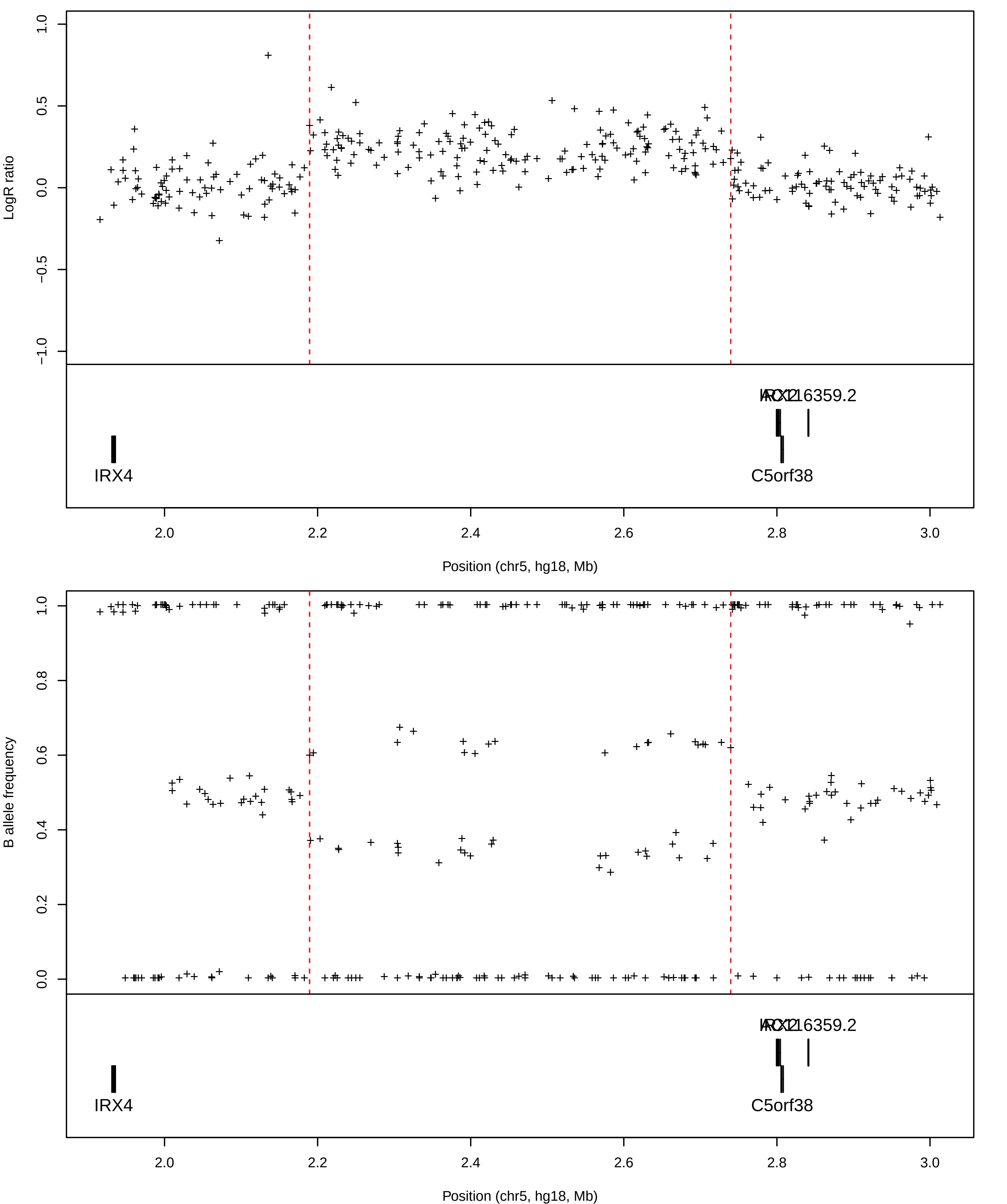

Figure S4−20, sample: 4343211465_R02C02, Internal ID:124.1, COLOMBIA, case, chr5:45400632−46196544

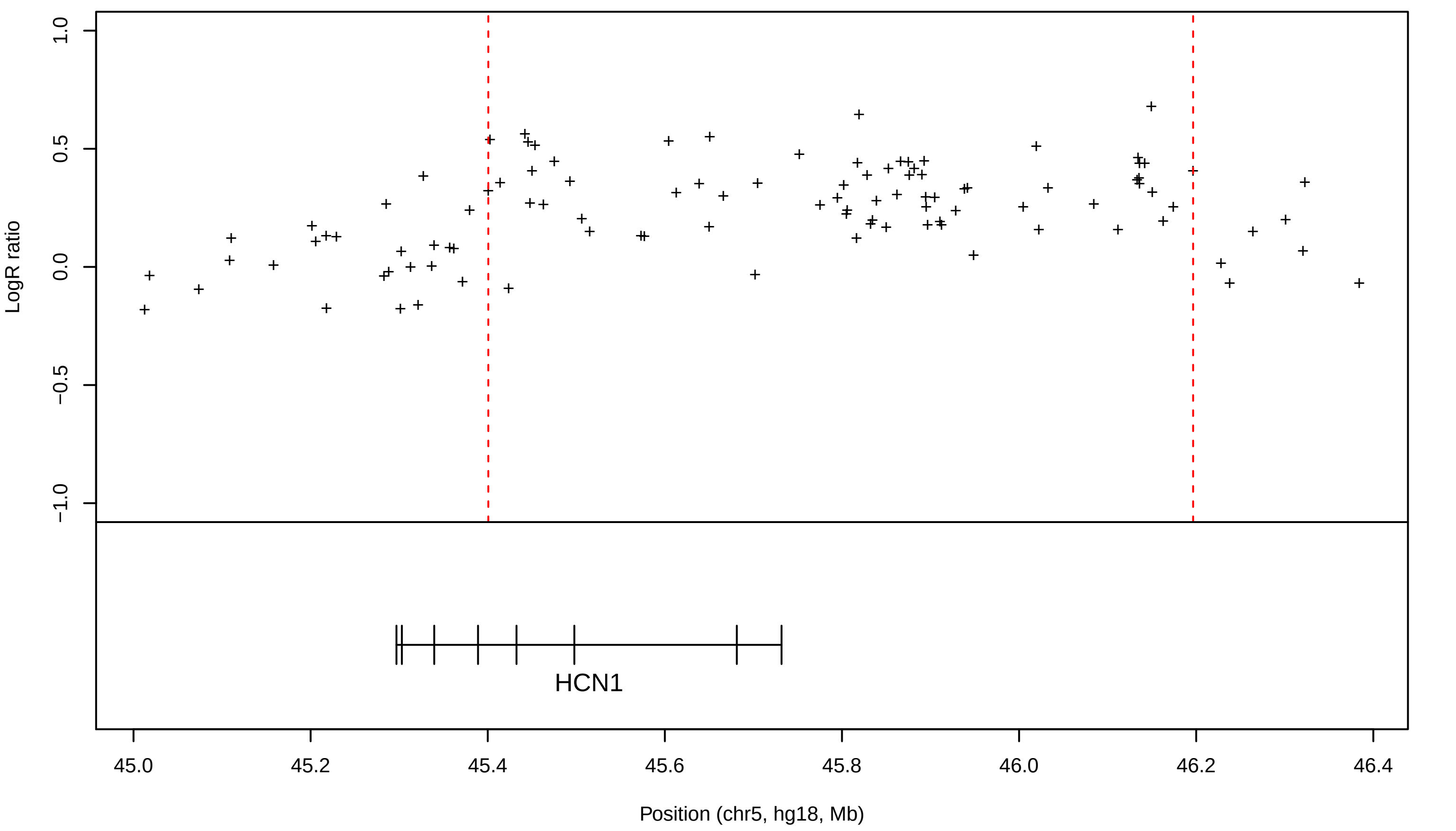

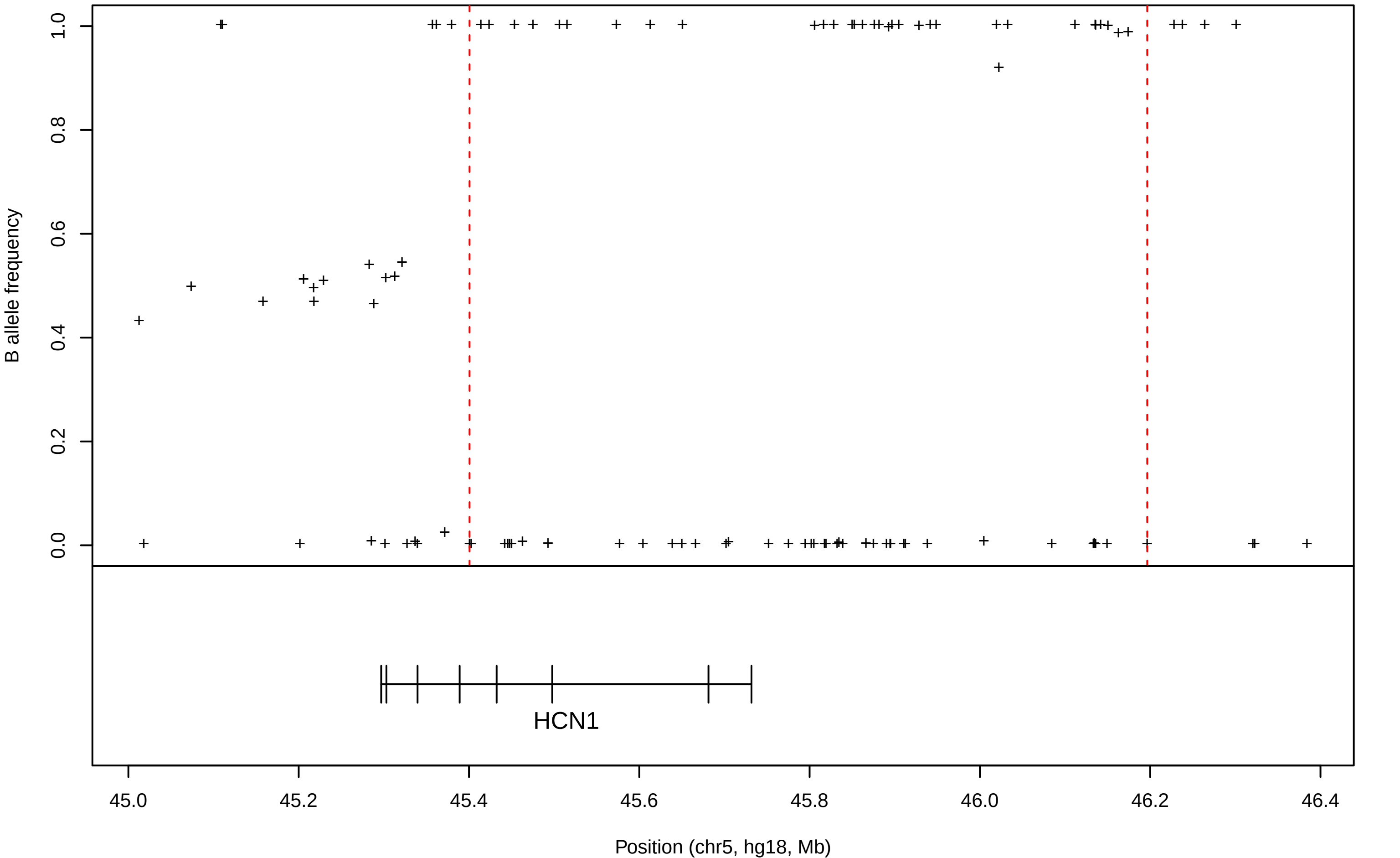

Figure S4−21, sample: 4393489522_R02C02, Internal ID:NA, COSTA RICA, case, chr5:101503405−102033686

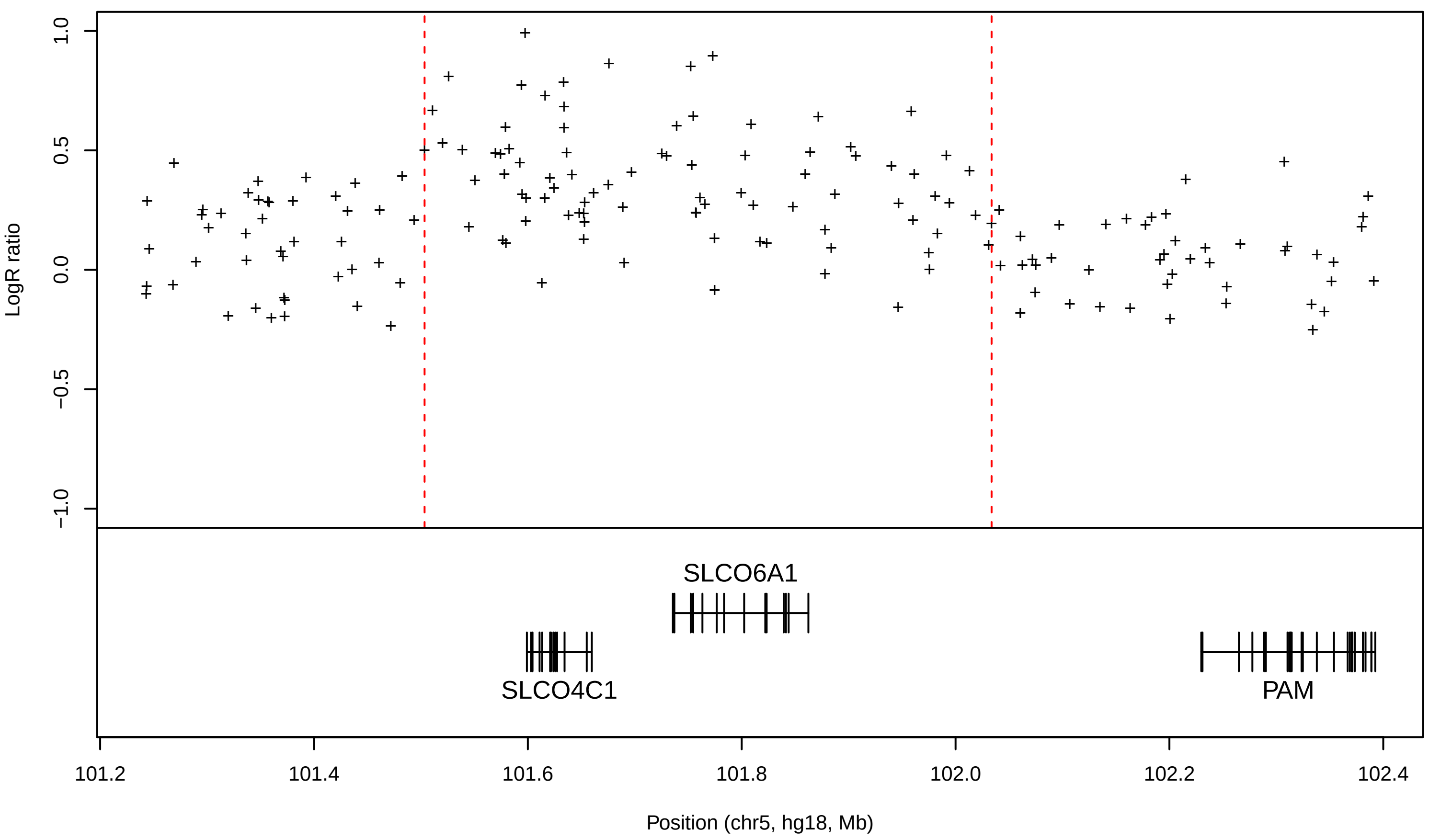

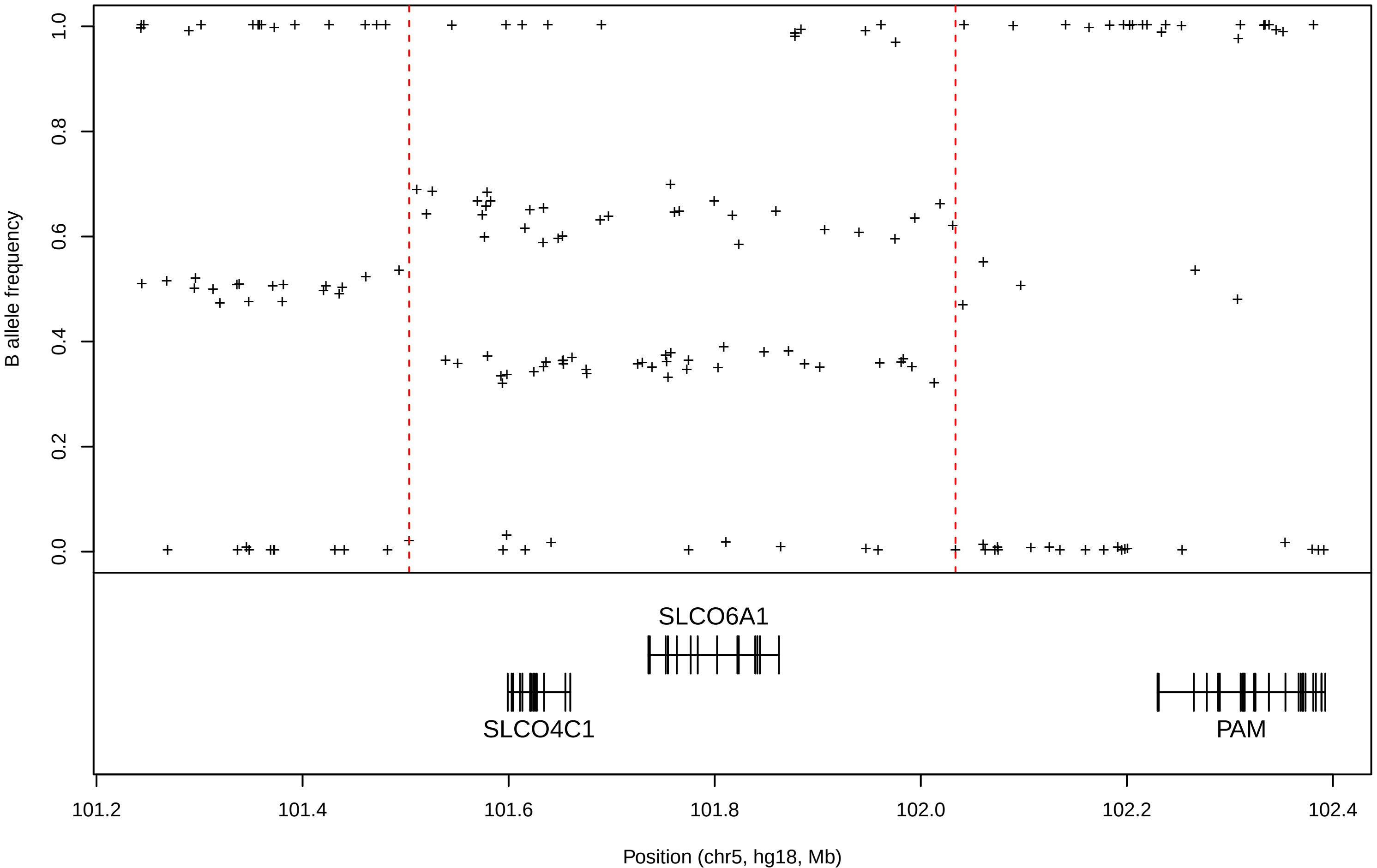

Figure S4−22, sample: 4408206717_R02C01, Internal ID:NA, COSTA RICA, case, chr5:101532676−102033686

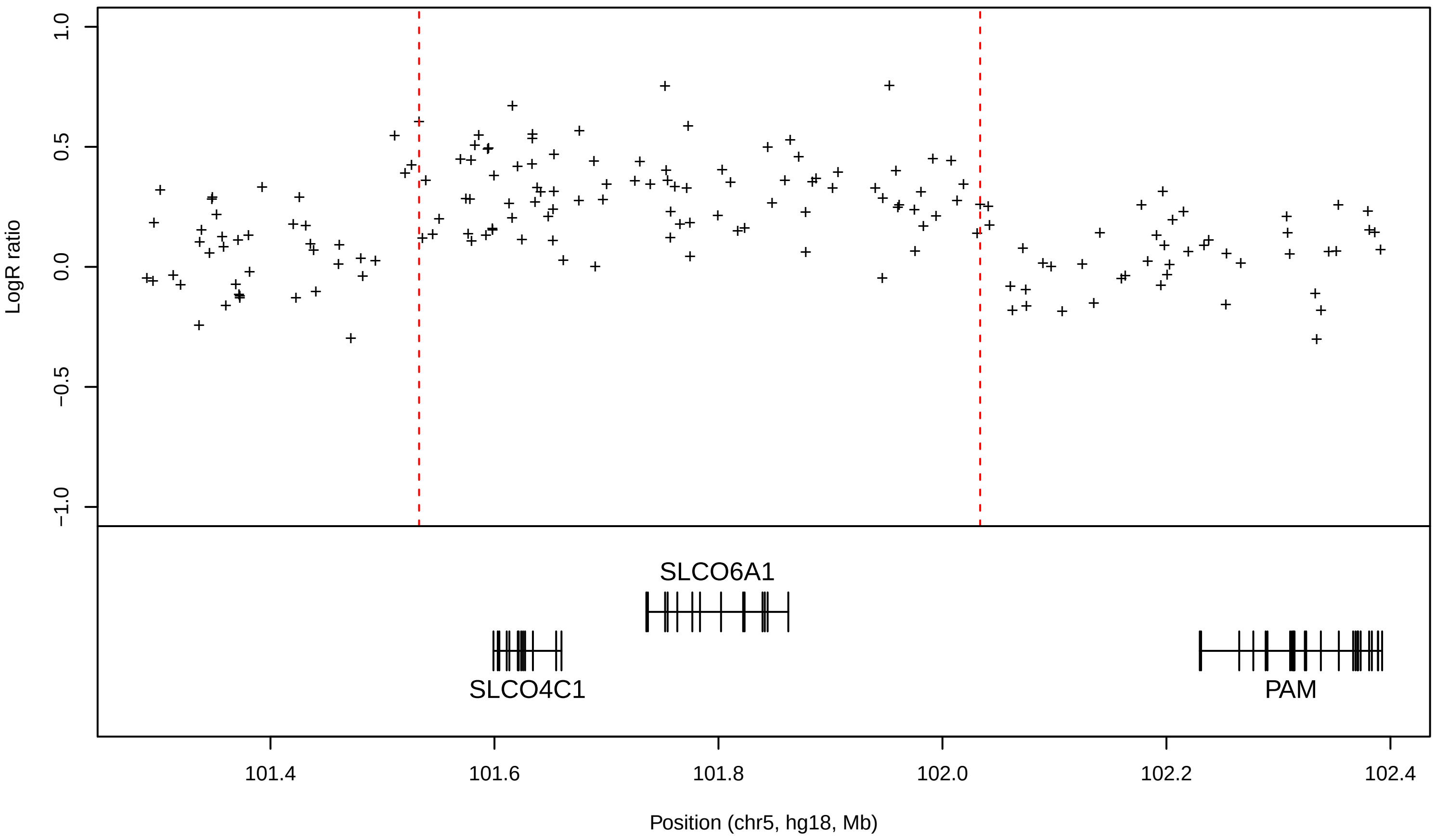

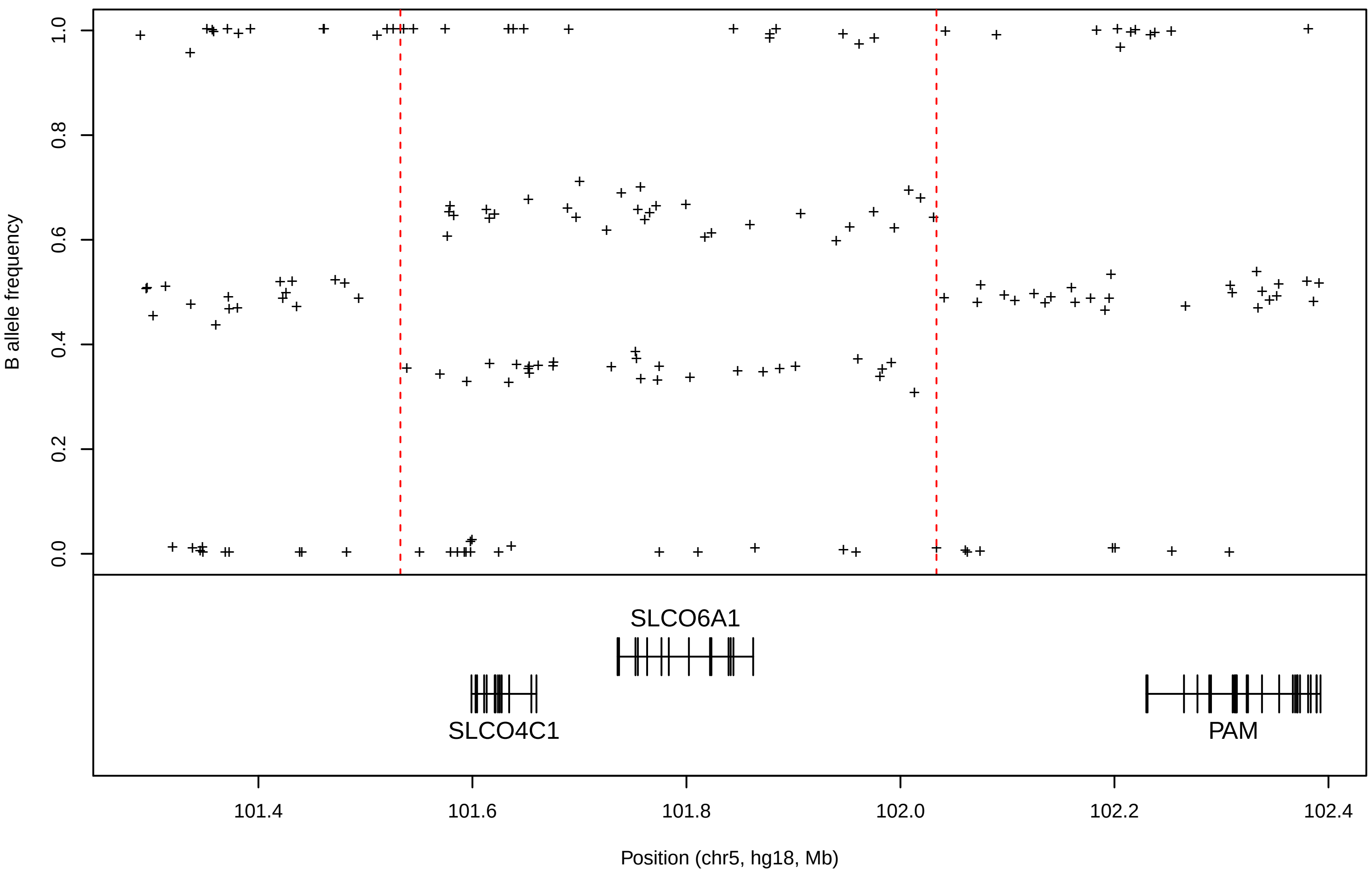

Figure S4−23, sample: 4343211438_R01C01, Internal ID:C290, COLOMBIA, control, chr5:178346422−179620378

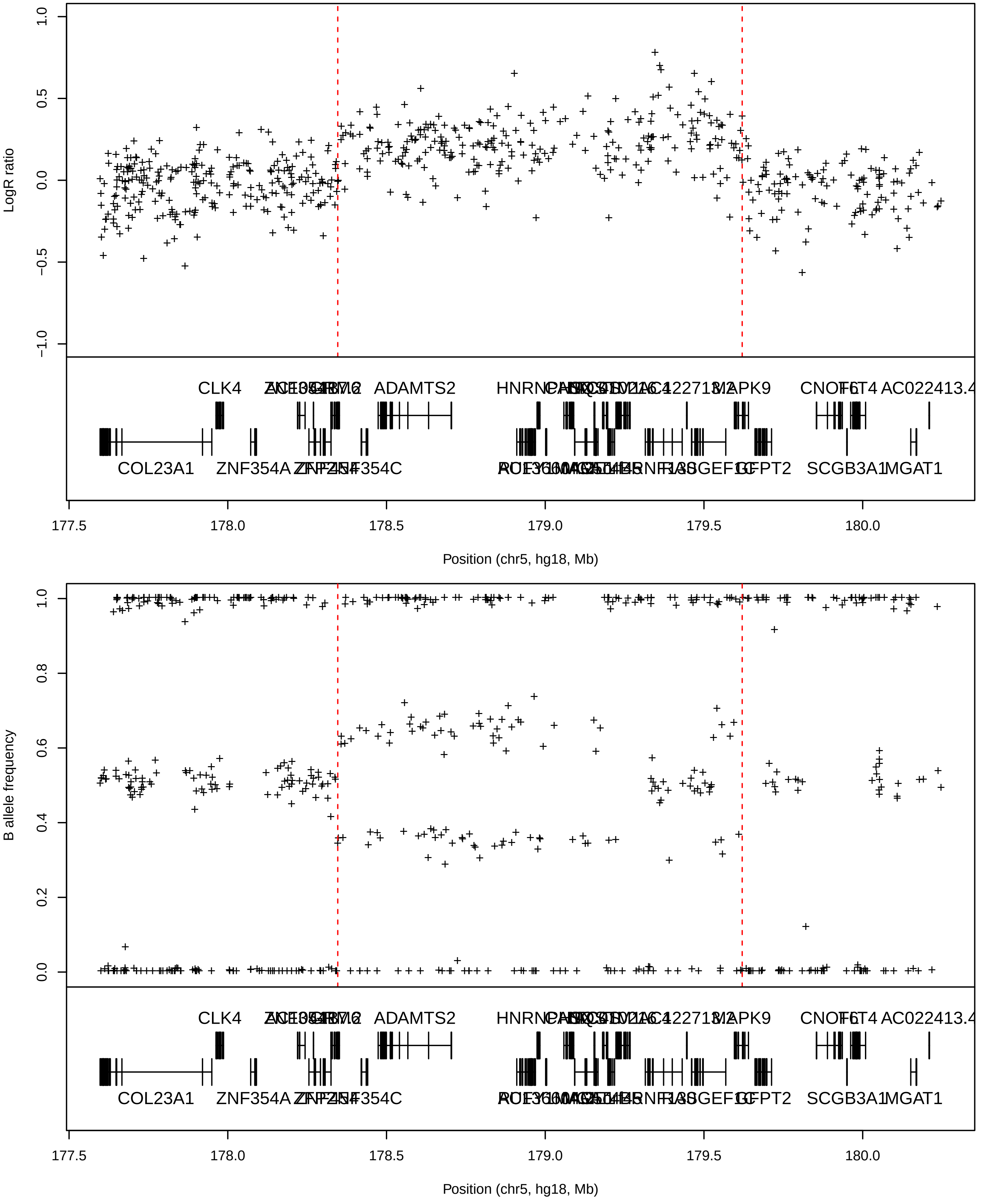

Figure S4−24, sample: 4506279055_R02C01, Internal ID:CP−089−2, COLOMBIA, control, chr7:16805611−17713153

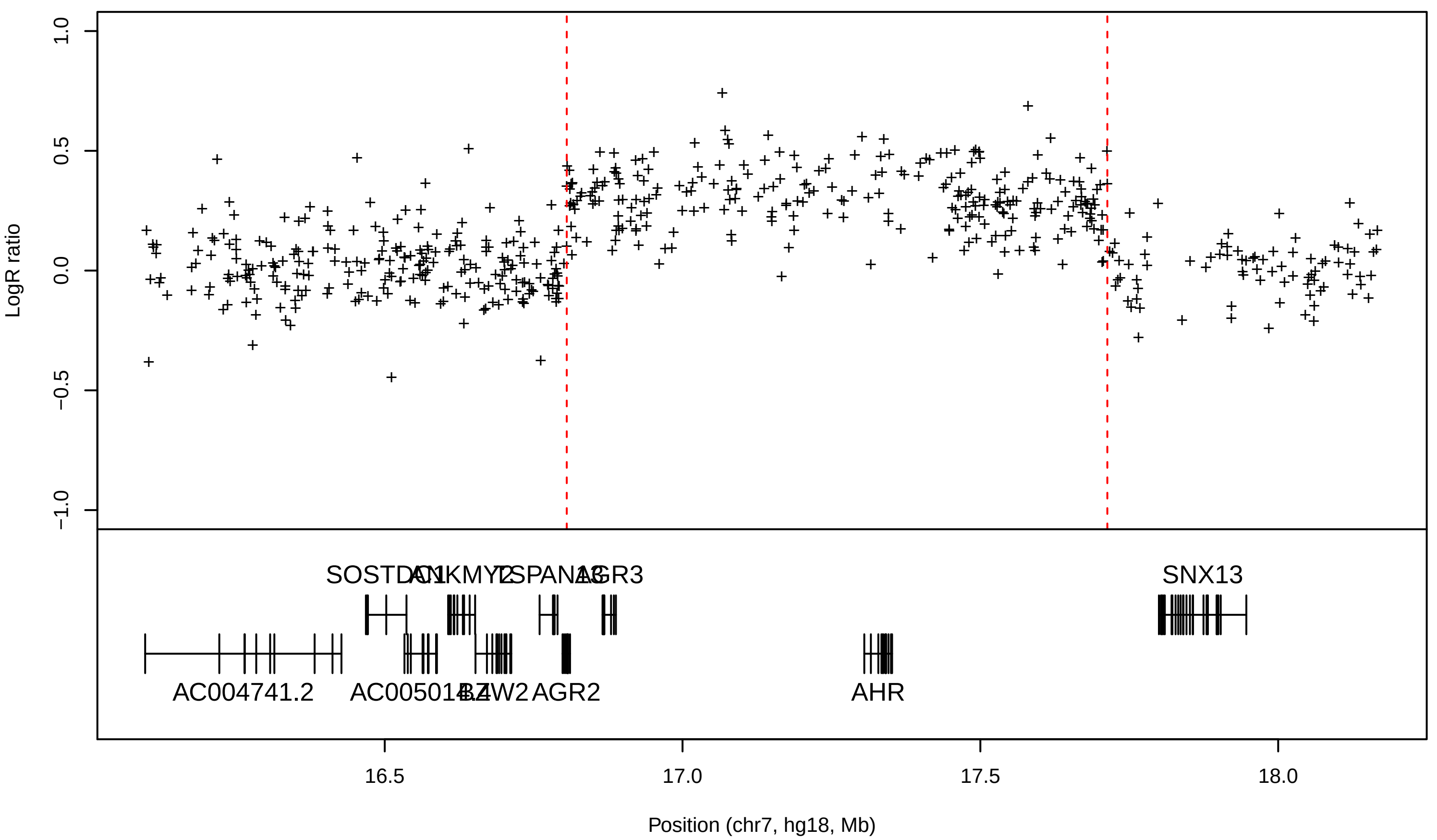

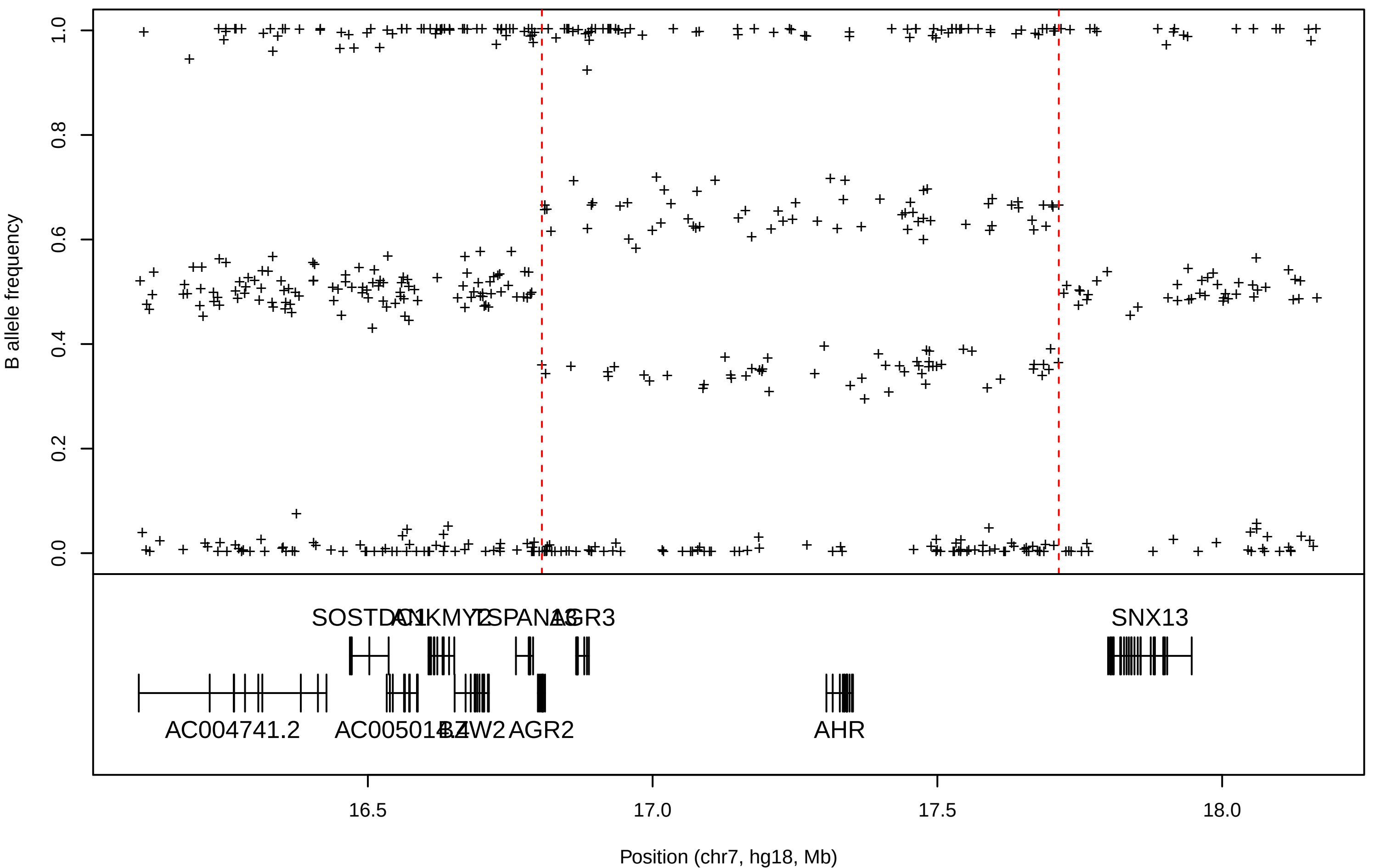

Figure S4−25, sample: 4506295002_R01C01, Internal ID:60.1, COLOMBIA, case, chr7:61681059−62336389

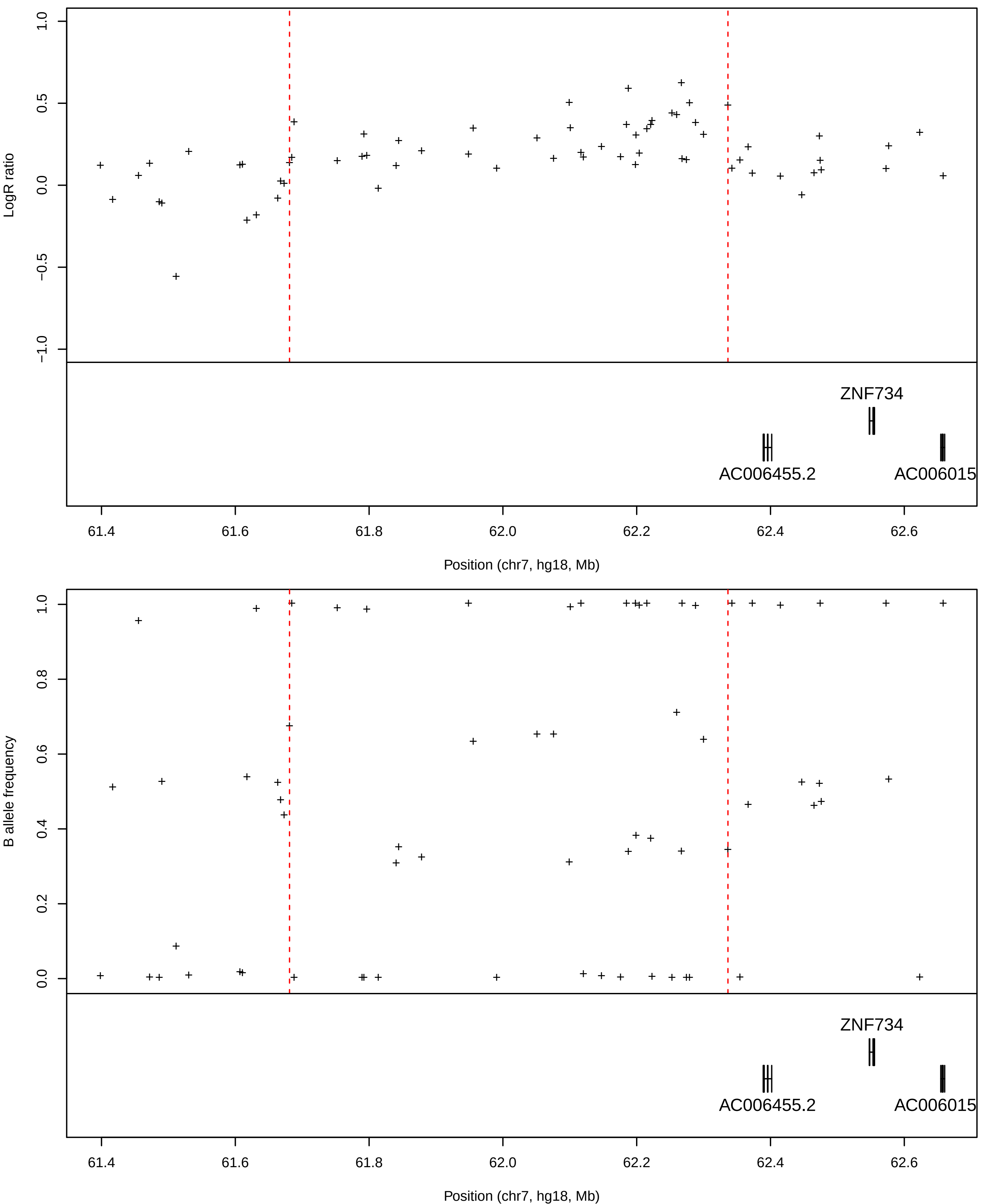

Figure S4−26, sample: 4393438068_R01C01, Internal ID:NA, COSTA RICA, case, chr8:2290333−3130717

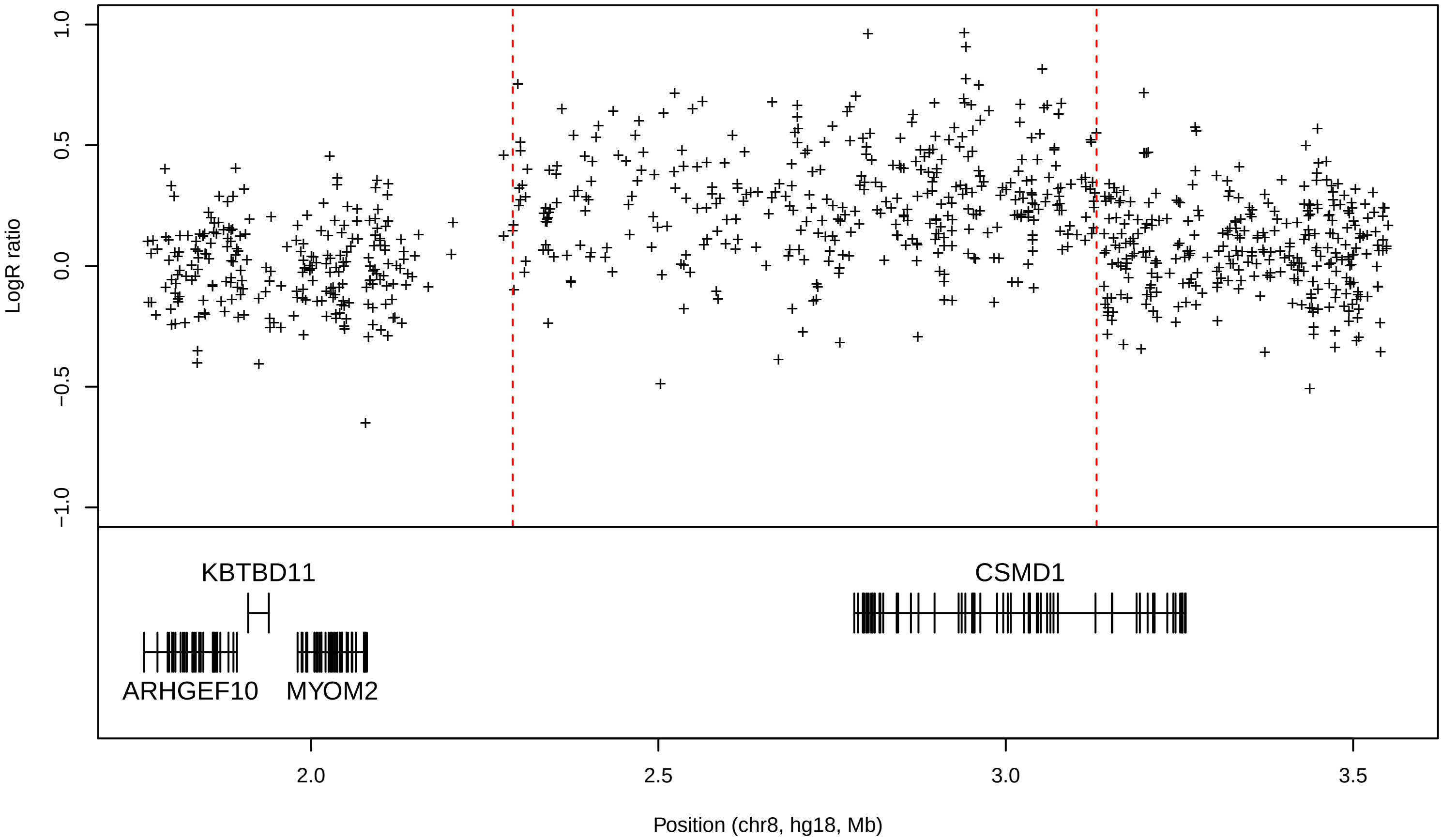

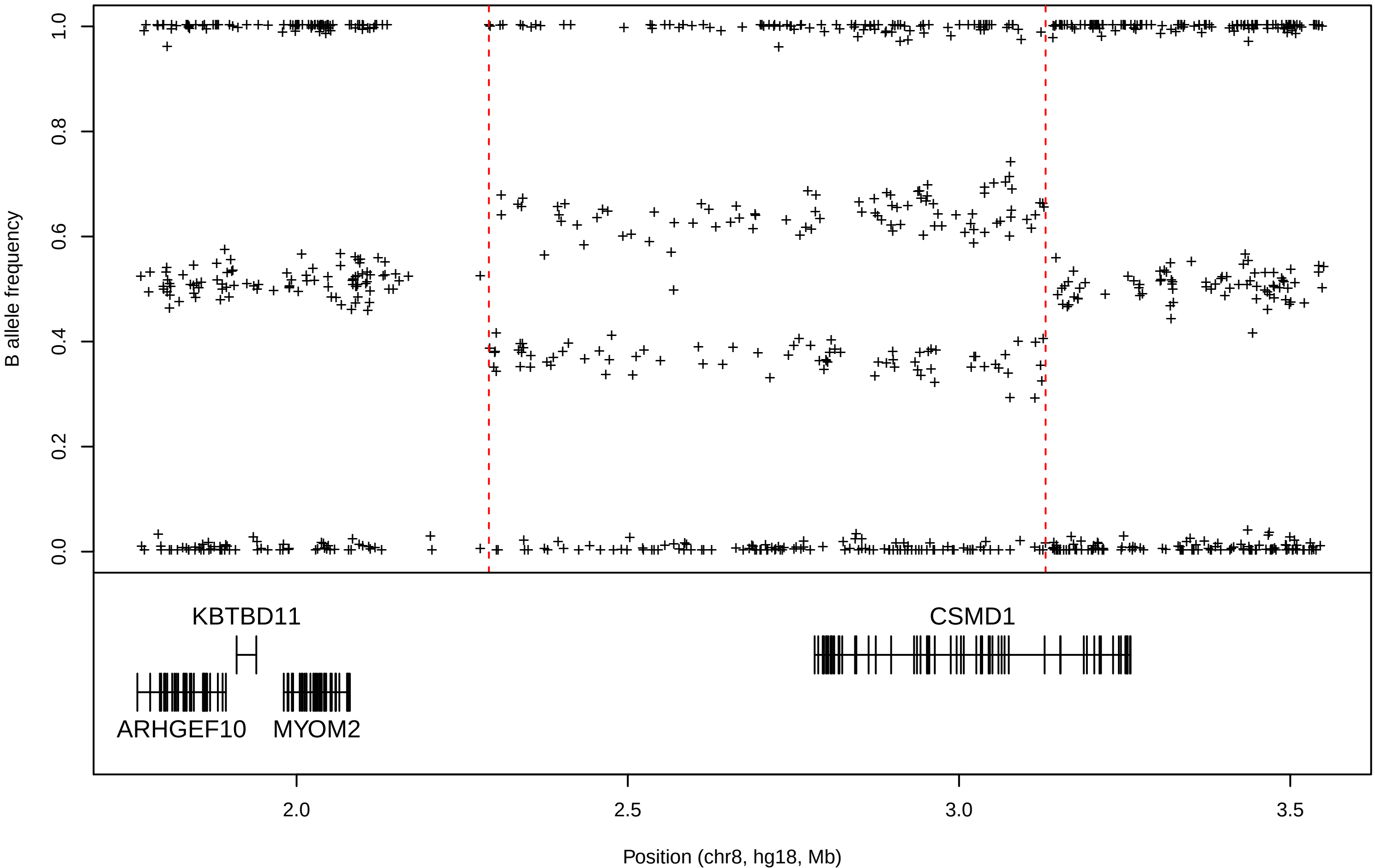

Figure S4−27, sample: 4506295006_R02C02, Internal ID:C14, COLOMBIA, control, chr8:2334306−2883118

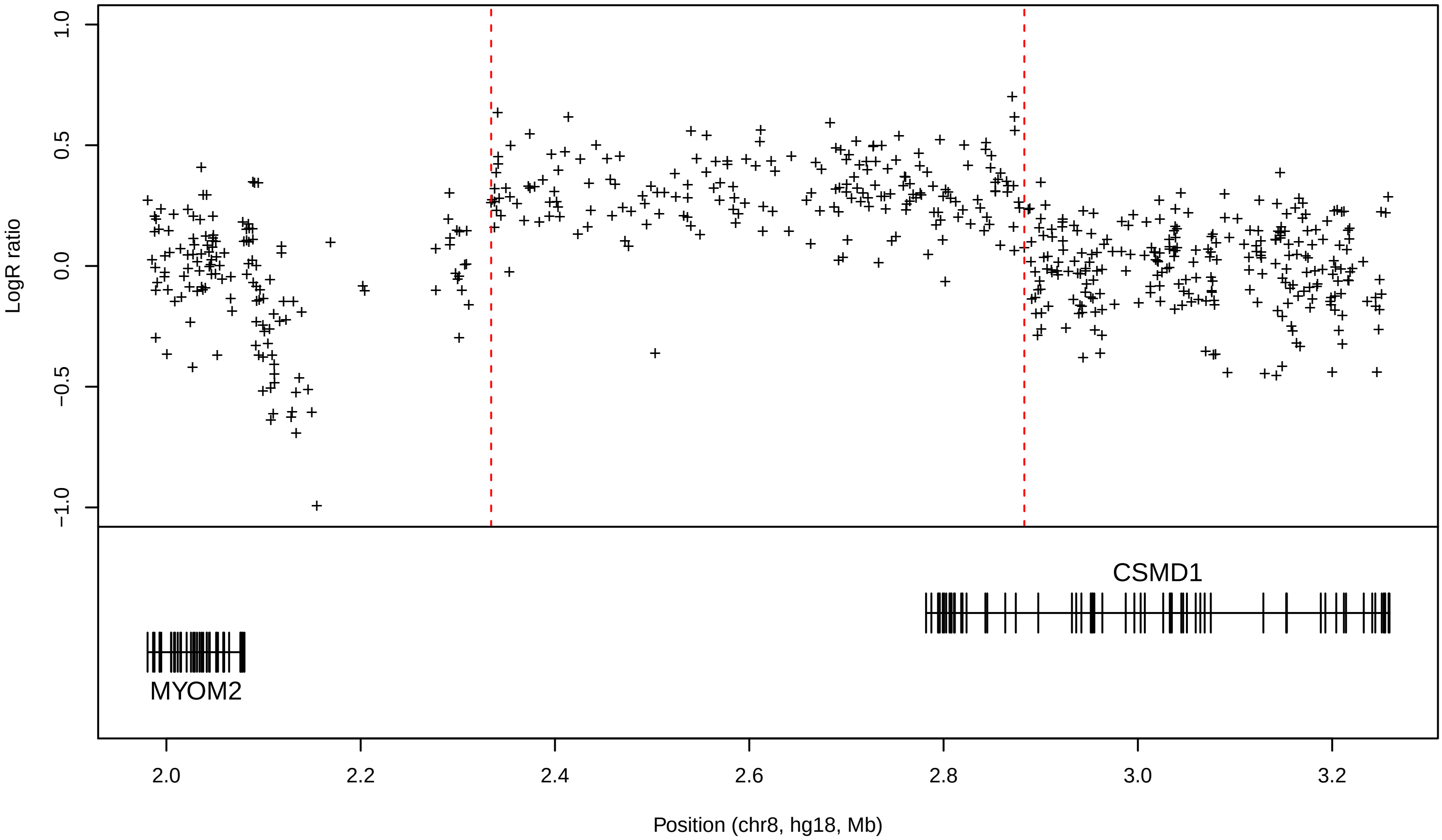

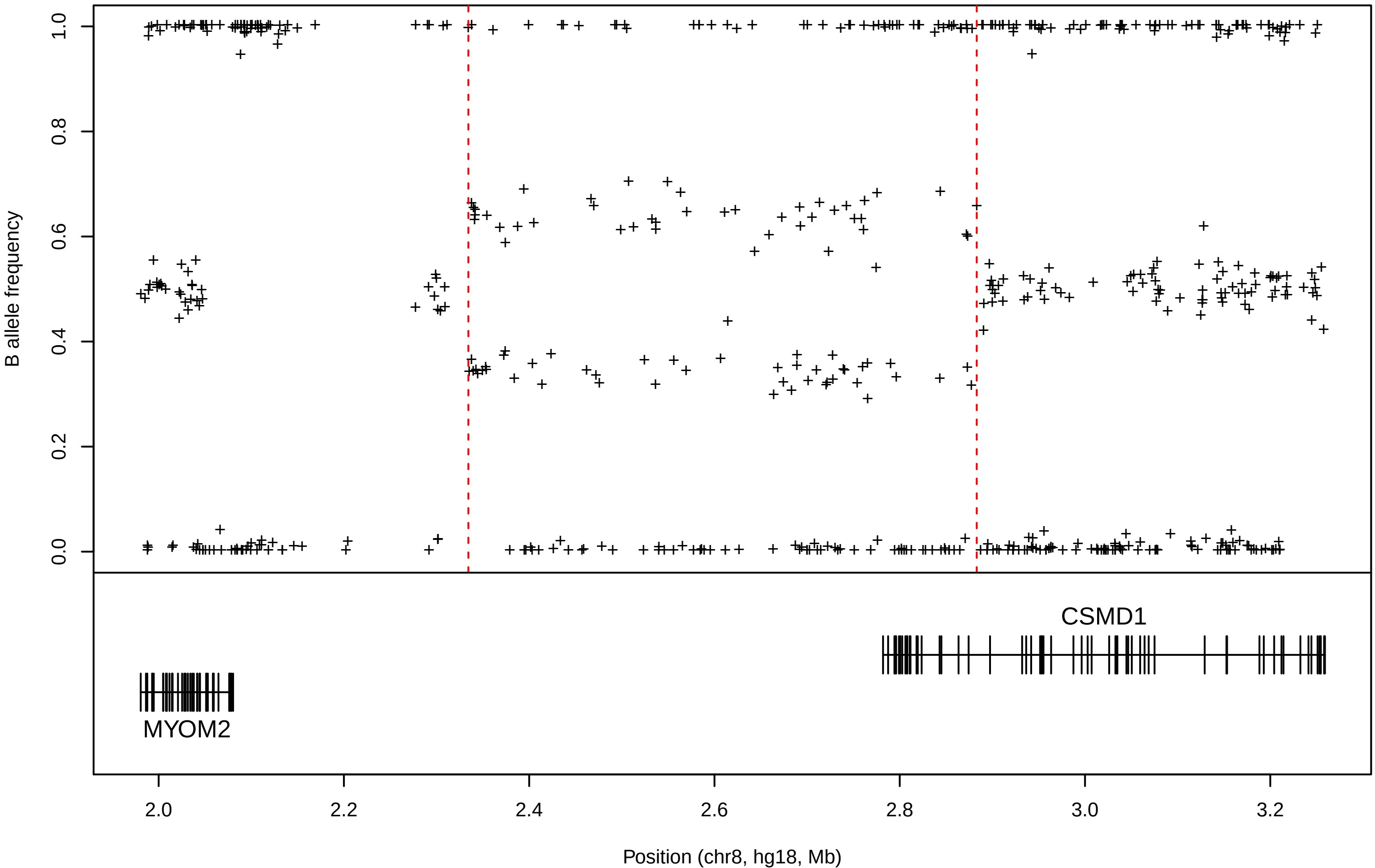

Figure S4−28, sample: 4343211598_R02C02, Internal ID:5.1, COLOMBIA, case, chr8:2453716−3126658

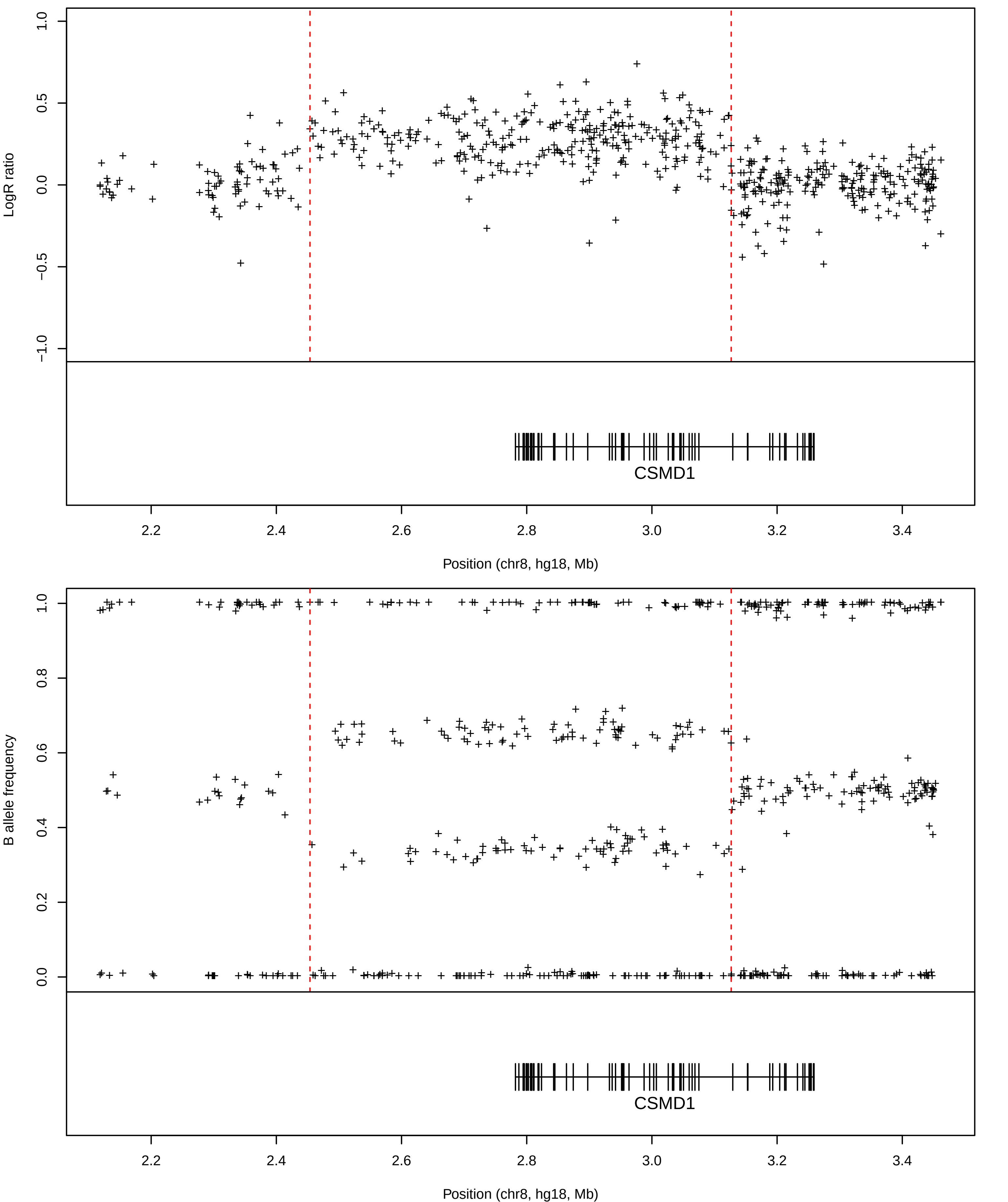

Figure S4−29, sample: 4494431105_R01C02, Internal ID:CP−146−2, COLOMBIA, control, chr8:107876892−108458326

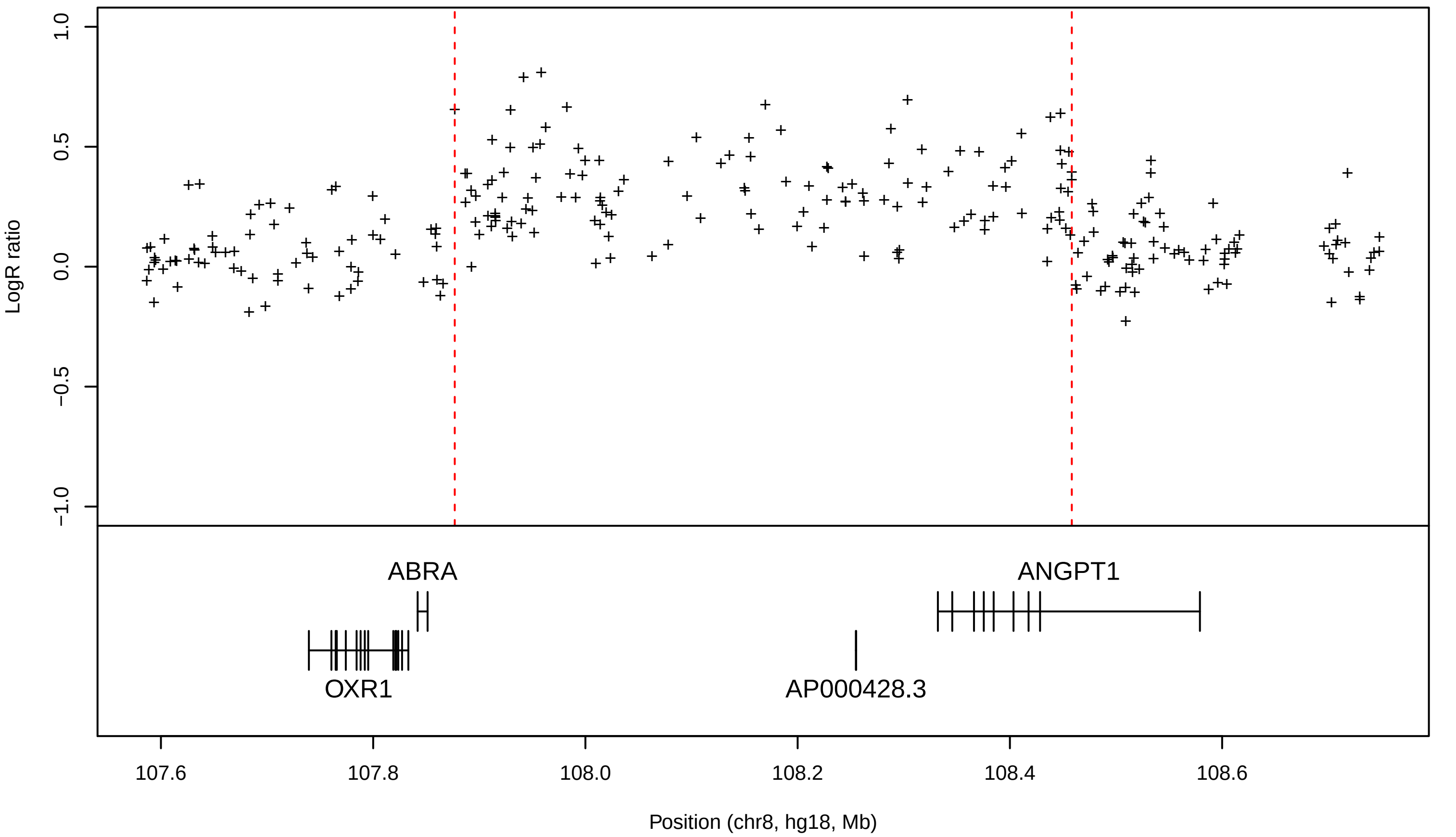

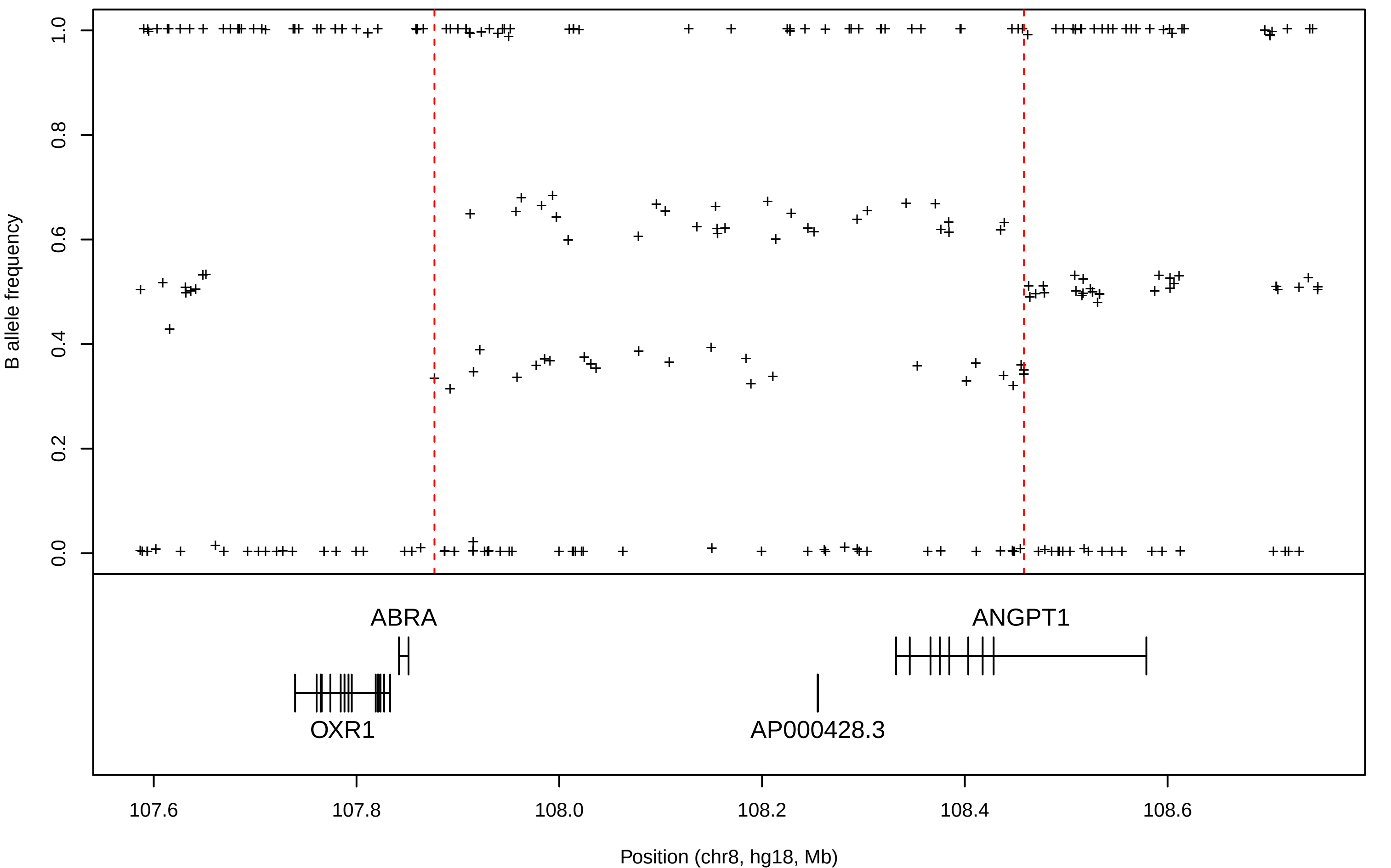

Figure S4−30, sample: 4381234507_R02C02, Internal ID:C303, COLOMBIA, control, chr8:107886832−108458326

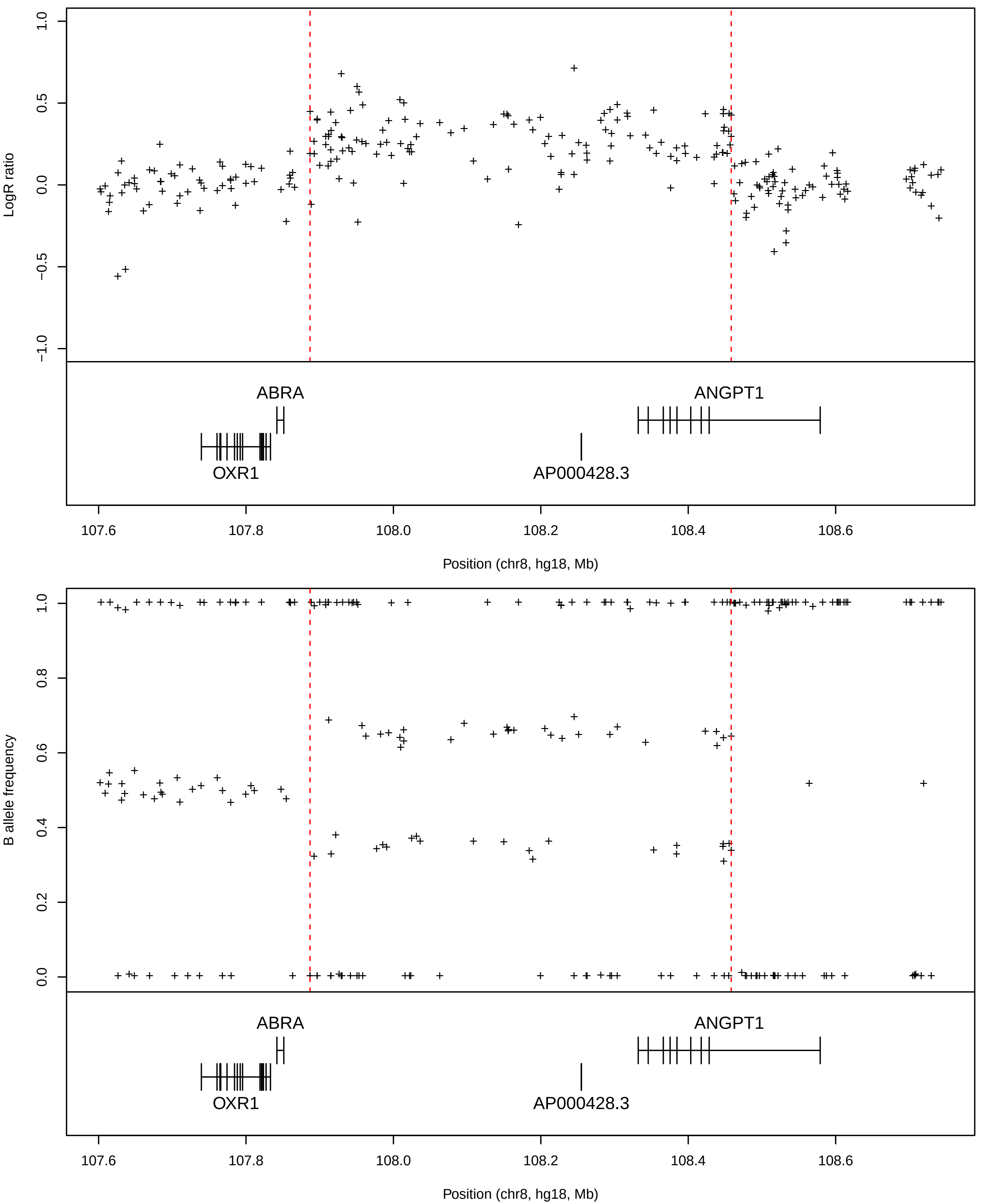

Figure S4−31, sample: 4381234044_R02C01, Internal ID:C488, COLOMBIA, control, chr8:107892324−108458326

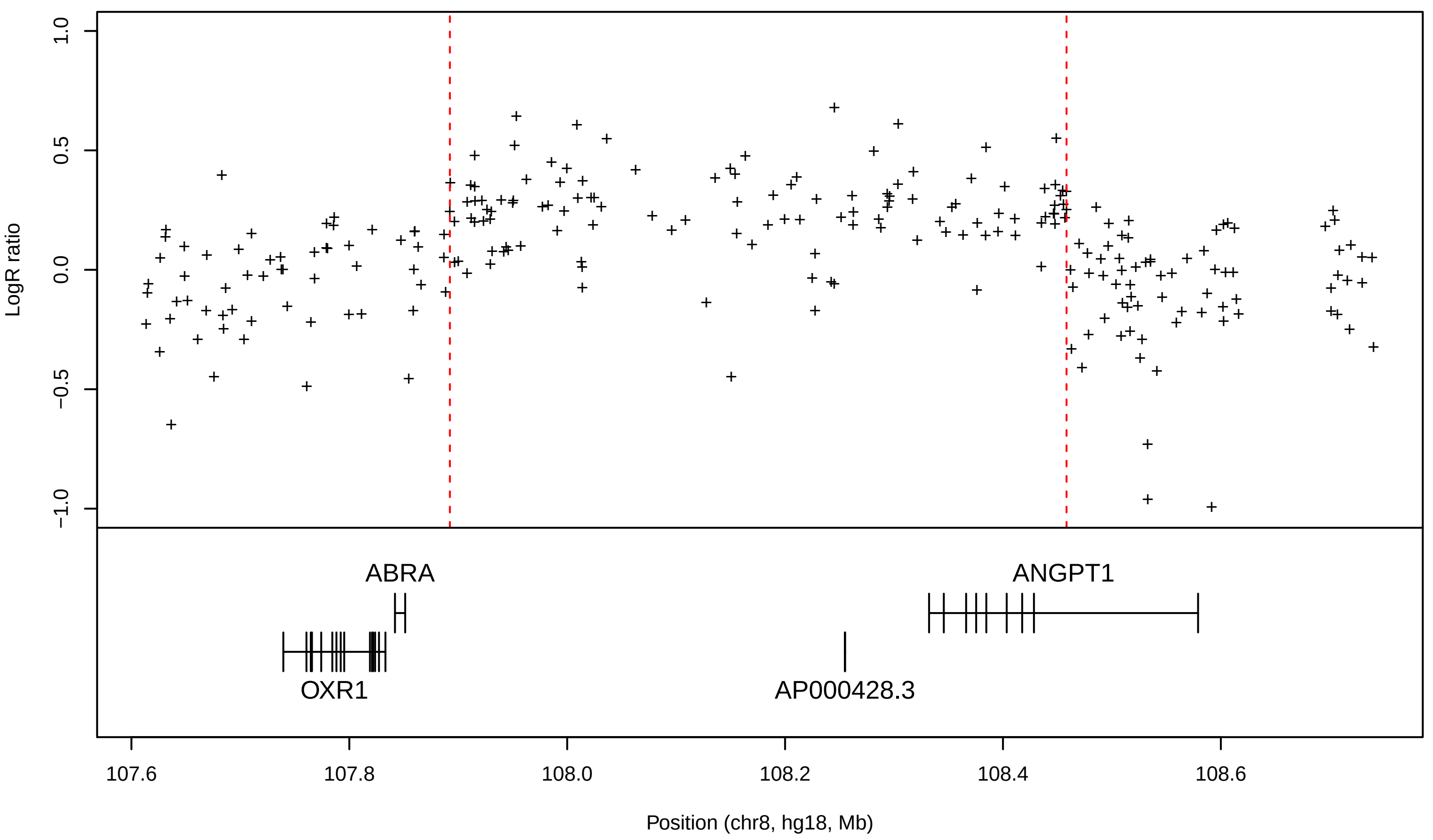

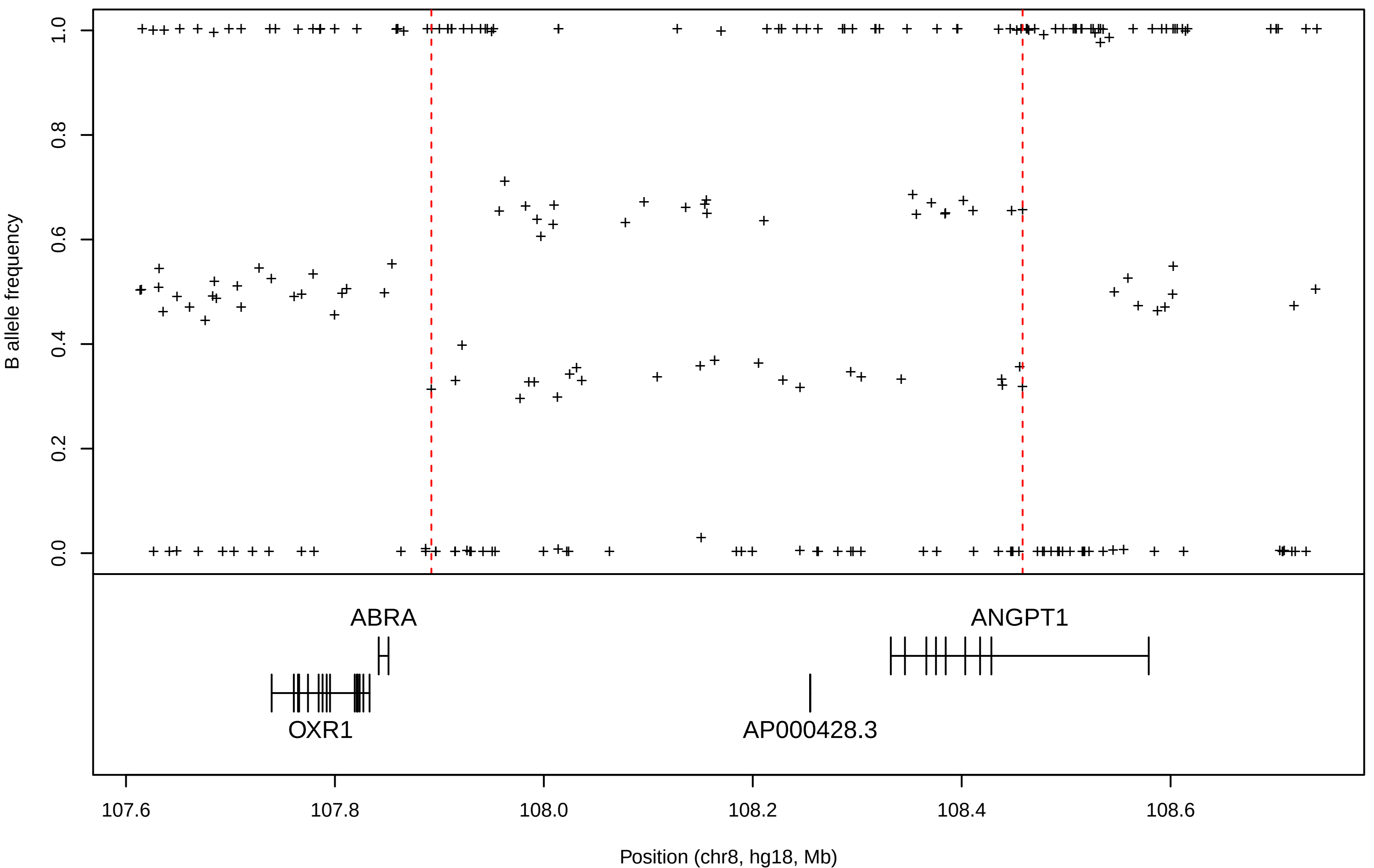

Figure S4−32, sample: 4378641751_R02C01, Internal ID:NA, COSTA RICA, case, chr10:20010963−20578907

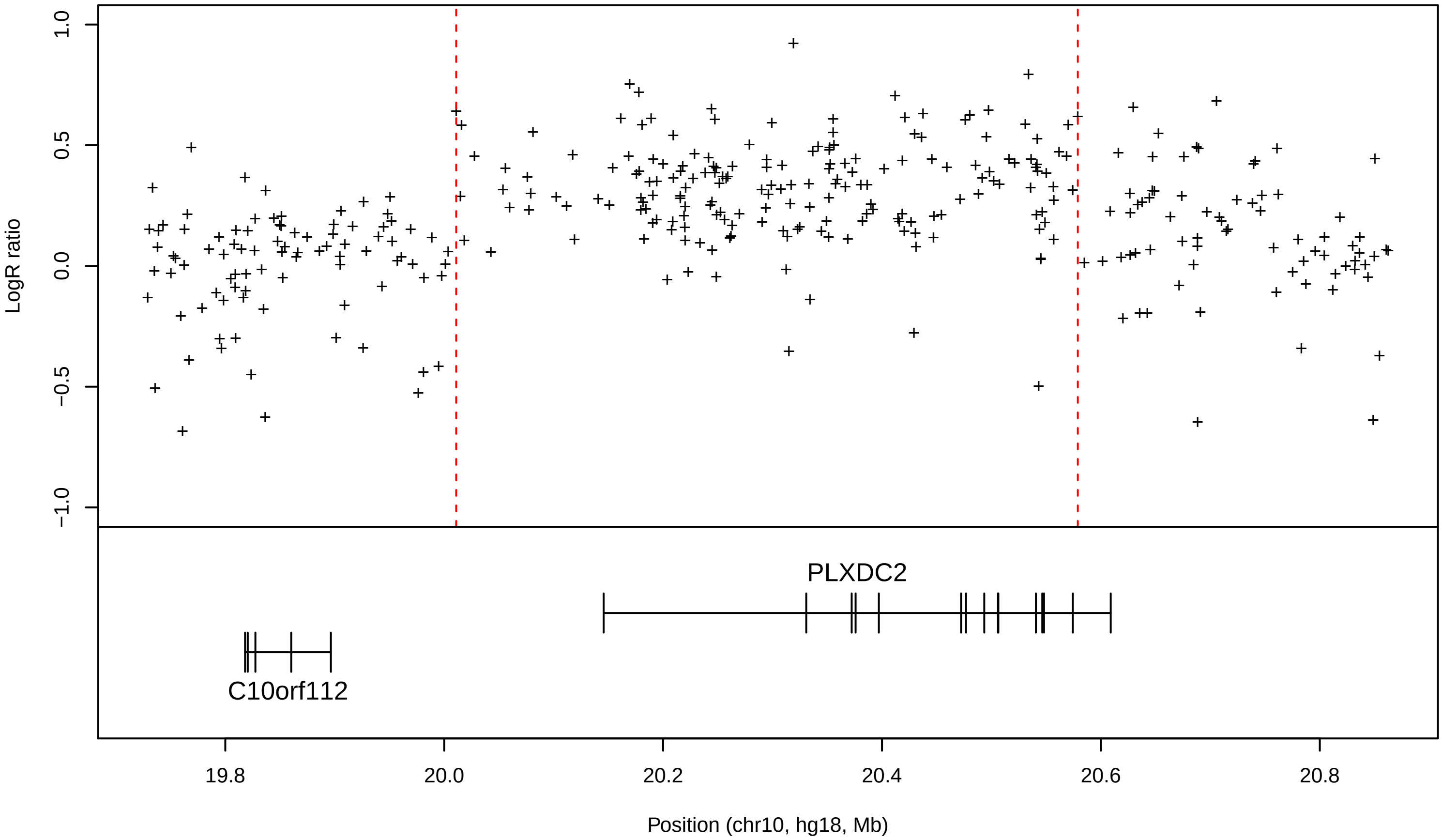

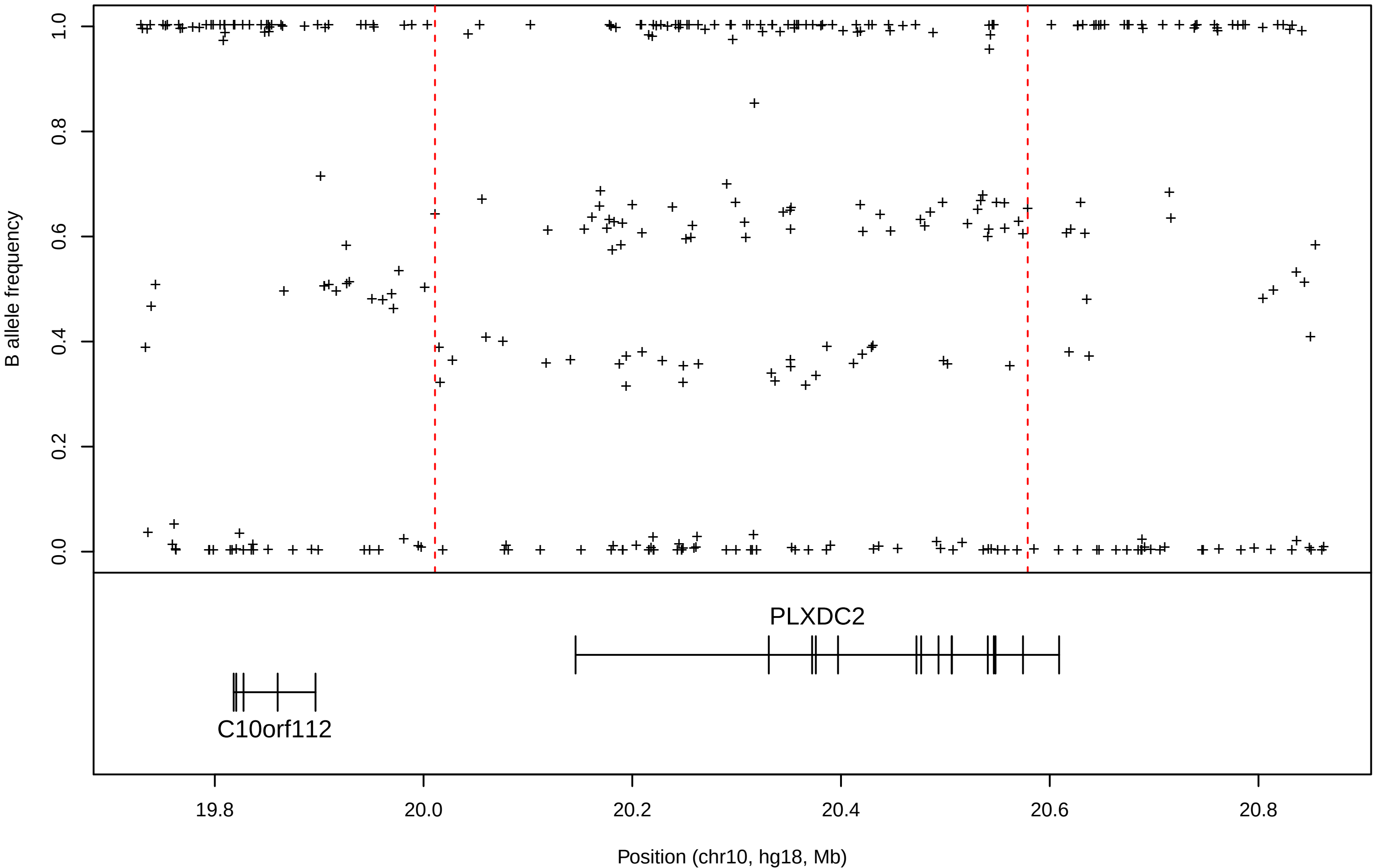

Figure S4−33, sample: 4506279093_R02C02, Internal ID:CP−112−2, COLOMBIA, control, chr10:46410734−47173619

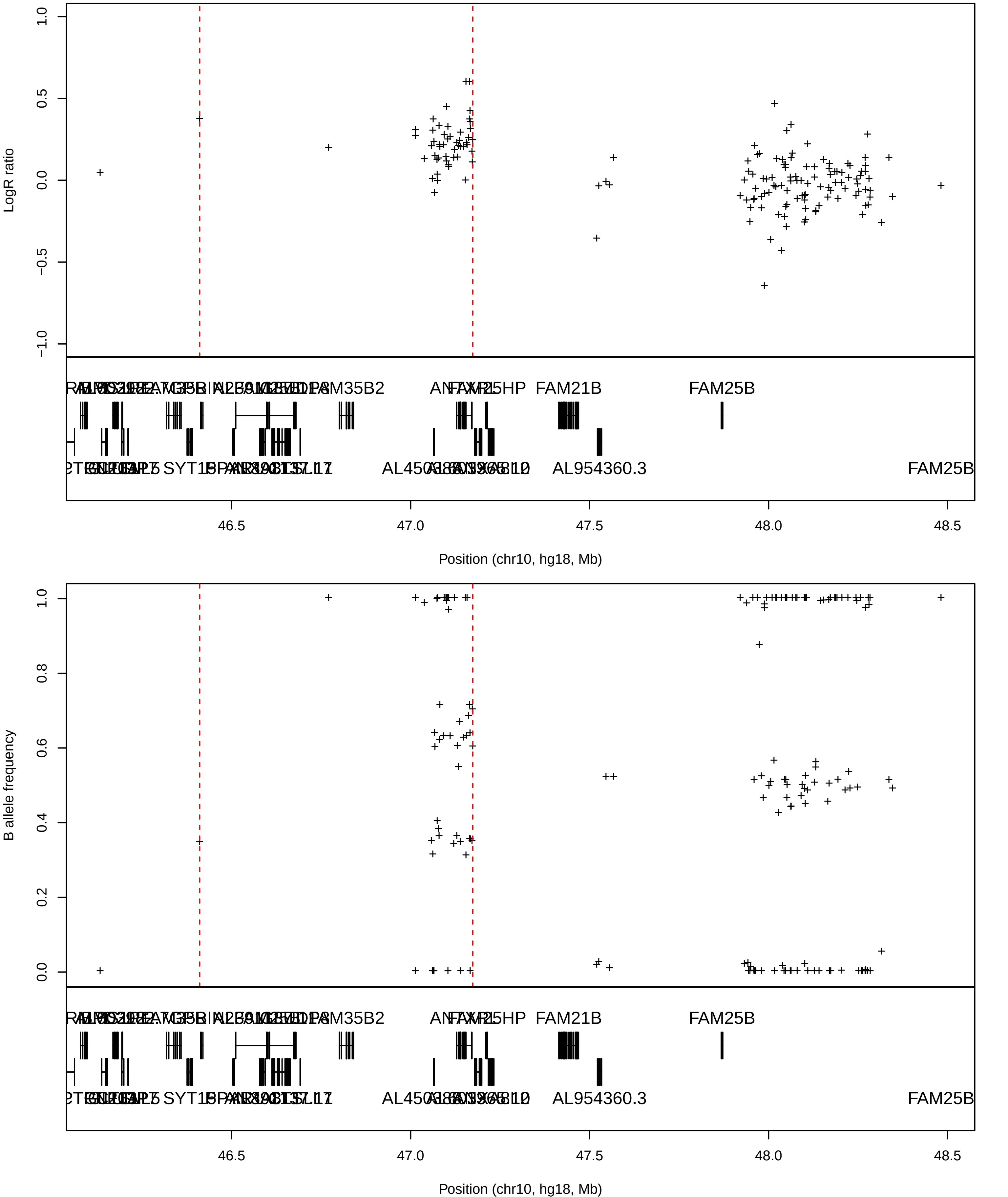

Figure S4−34, sample: 4393489876_R02C01, Internal ID:NA, COSTA RICA, case, chr10:110413539−111108275

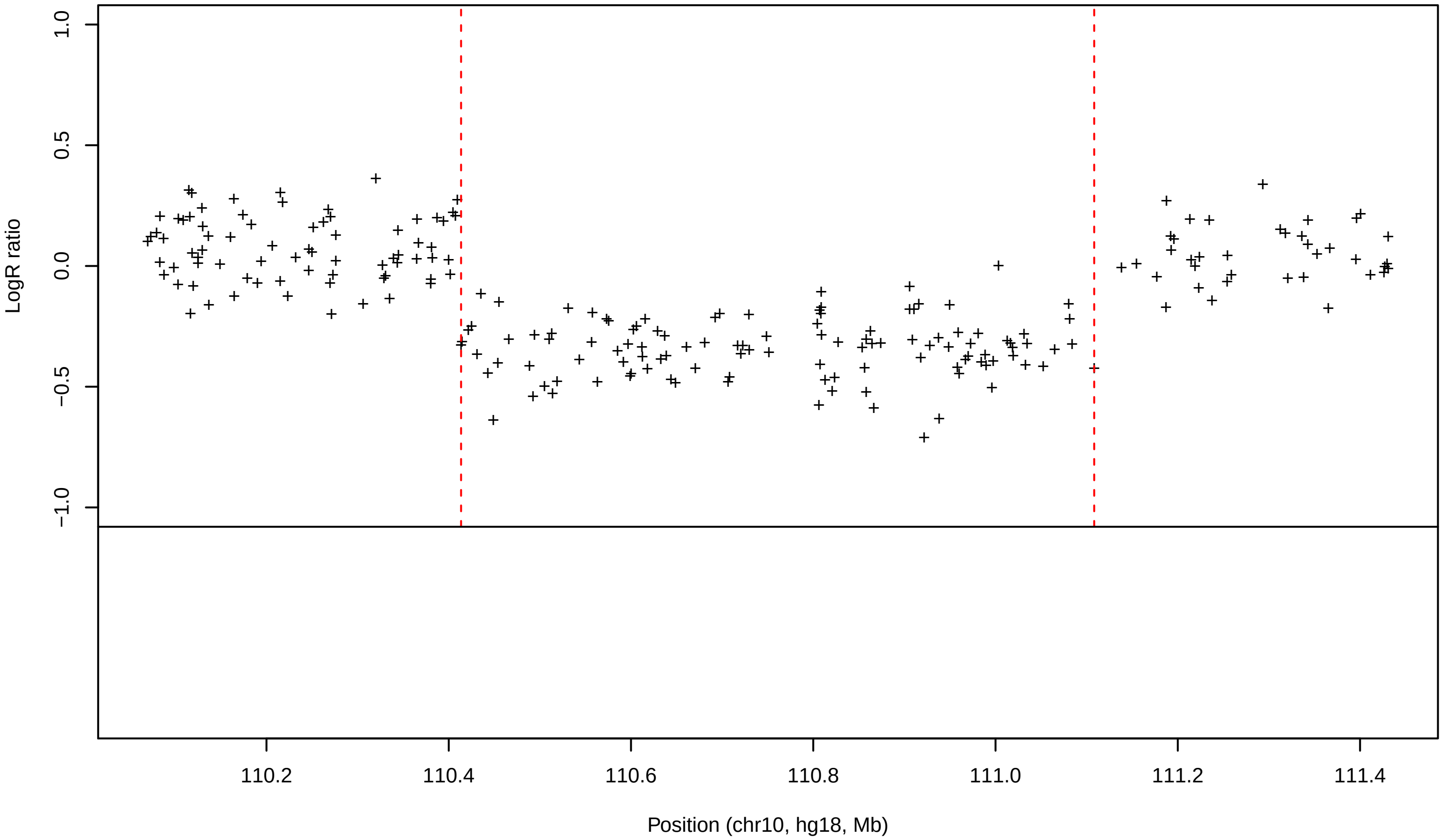

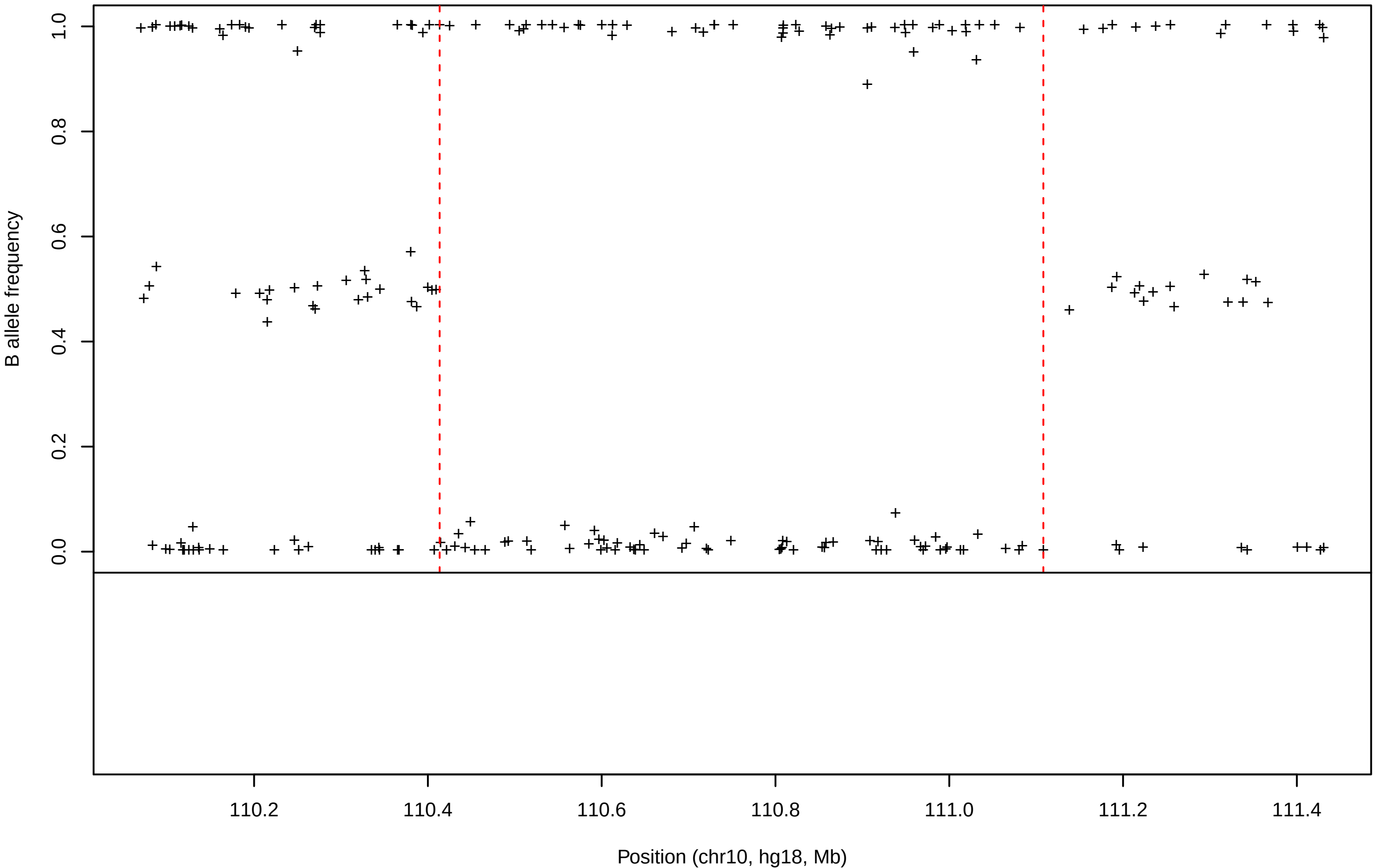

Figure S4−35, sample: 4378641129_R01C02, Internal ID:NA, COSTA RICA, case, chr11:54631614−55760165

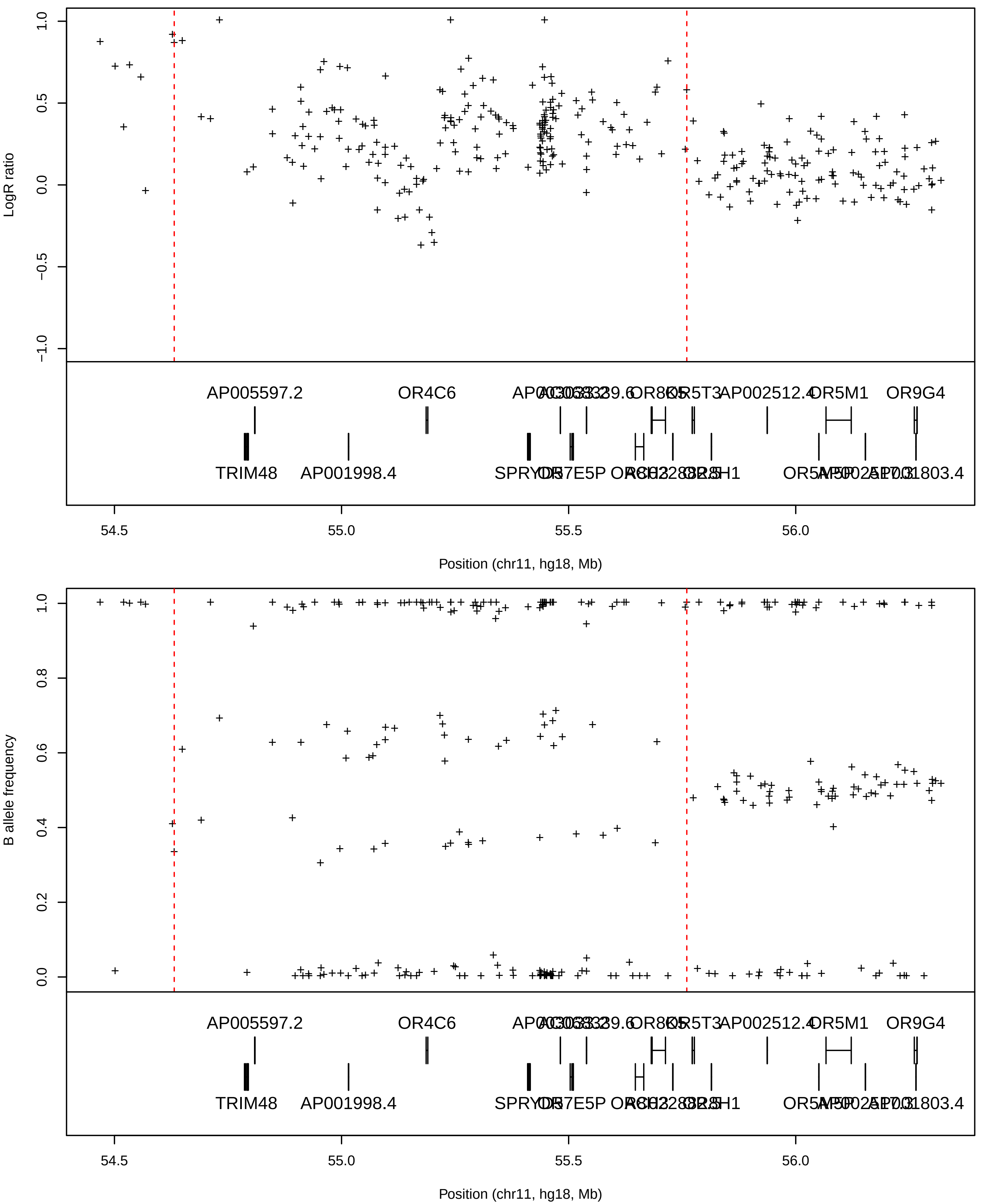

Figure S4−36, sample: 4381234048_R01C01, Internal ID:MAR 024, COLOMBIA, control, chr11:112369739−112956975

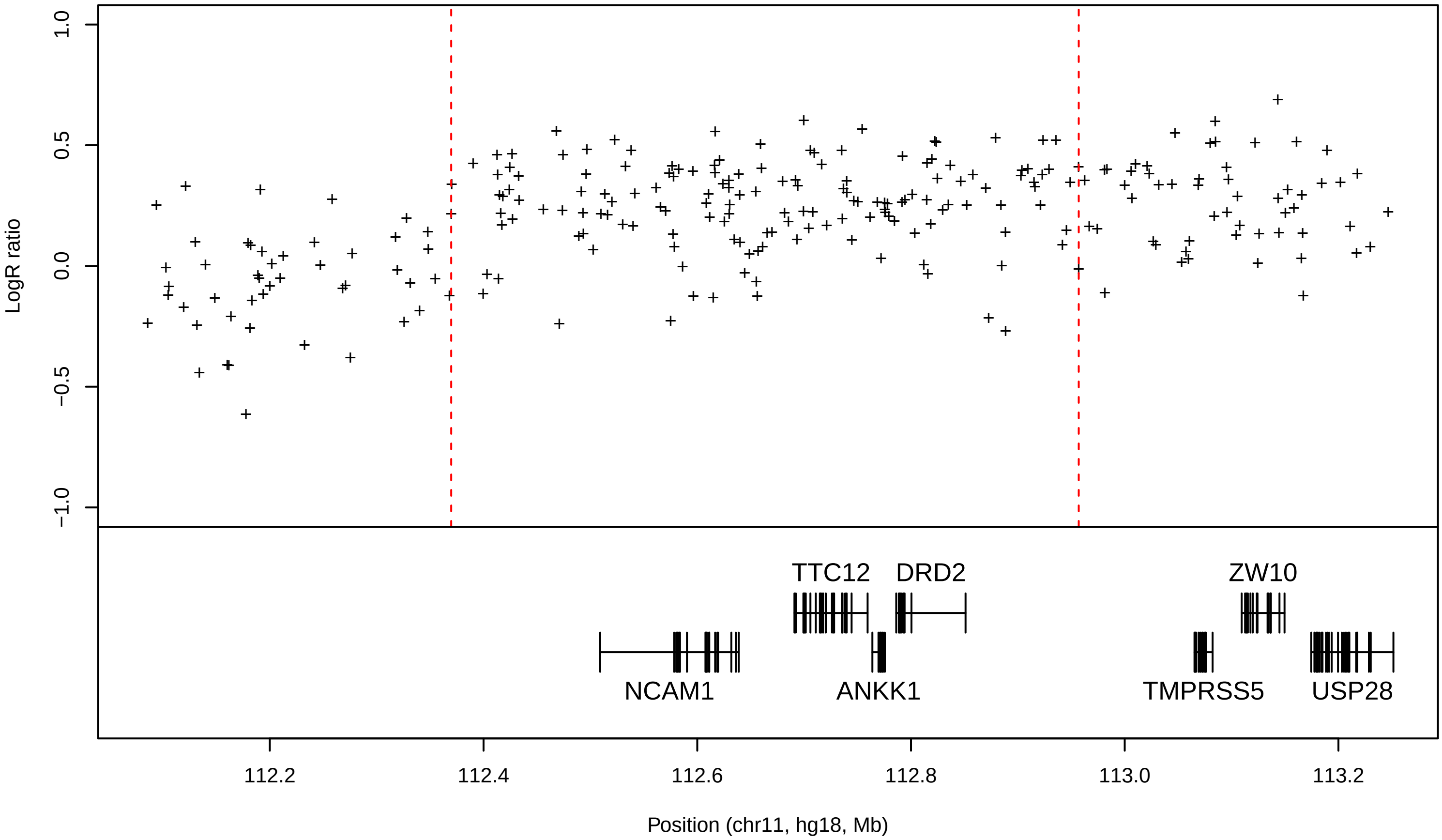

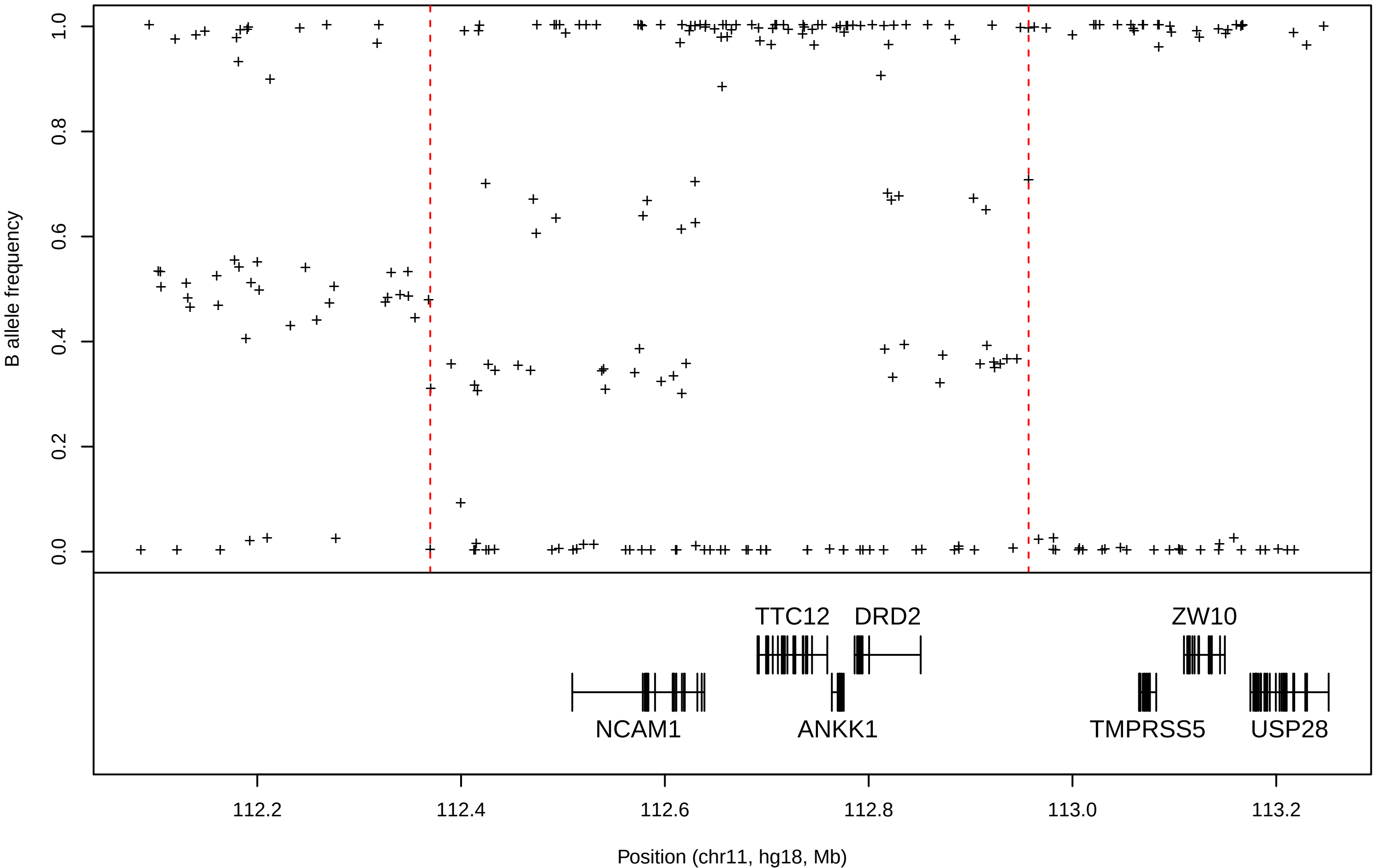

Figure S4−37, sample: 4378959456_R01C02, Internal ID:NA, COSTA RICA, case, chr12:38586180−39111754

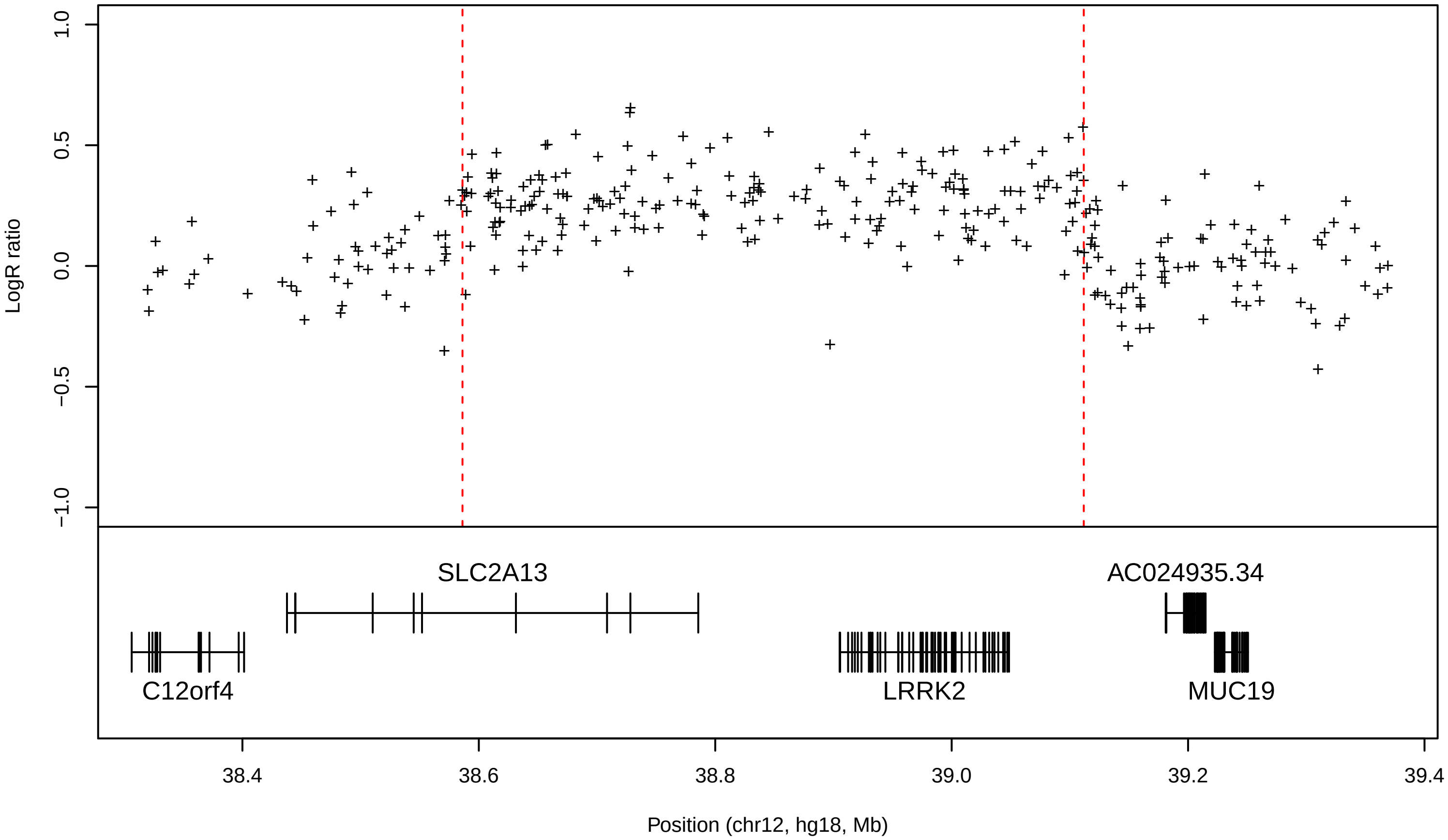

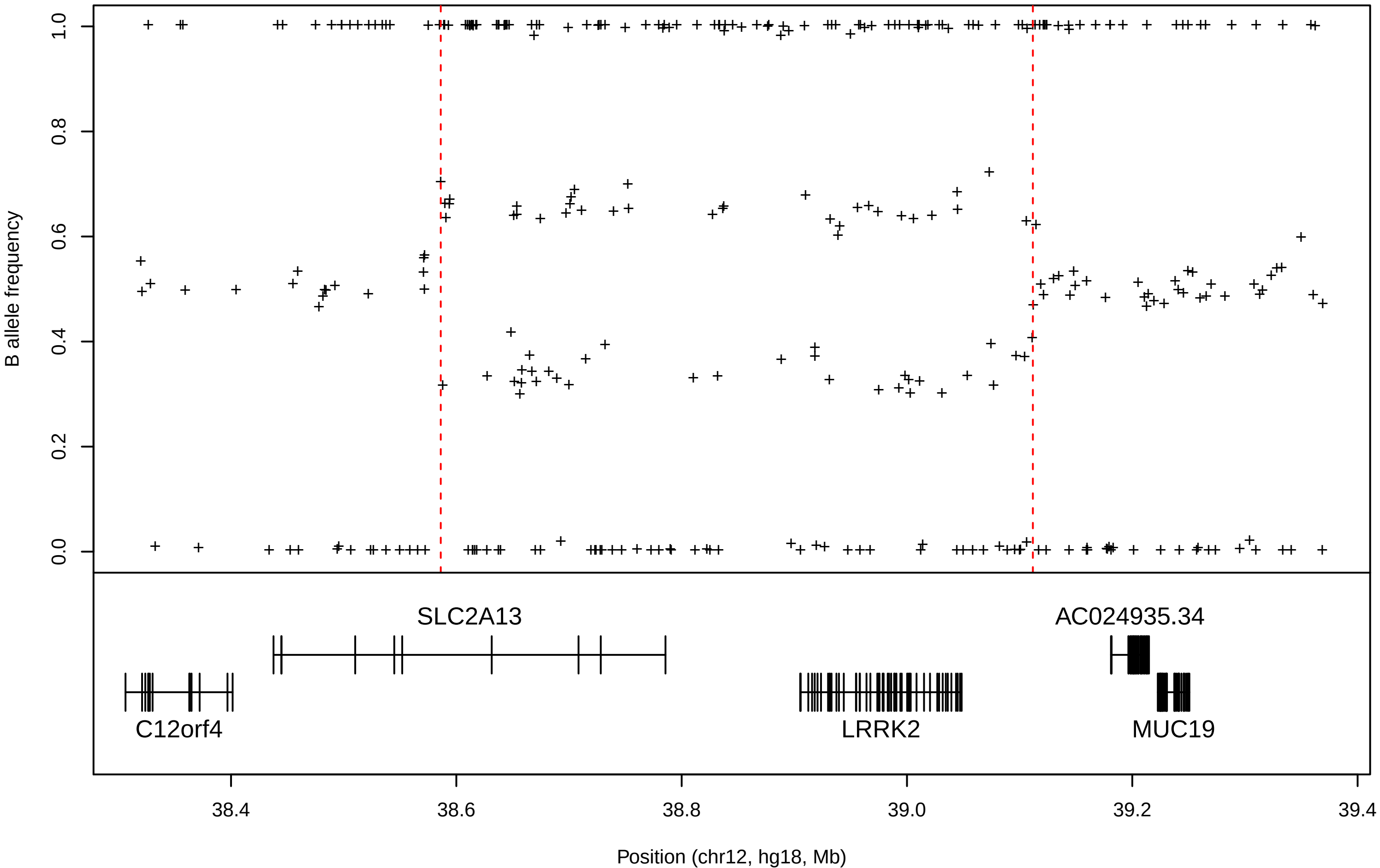

Figure S4−38, sample: 4506316062_R01C01, Internal ID:C224, COLOMBIA, control, chr17:14040467−15411904

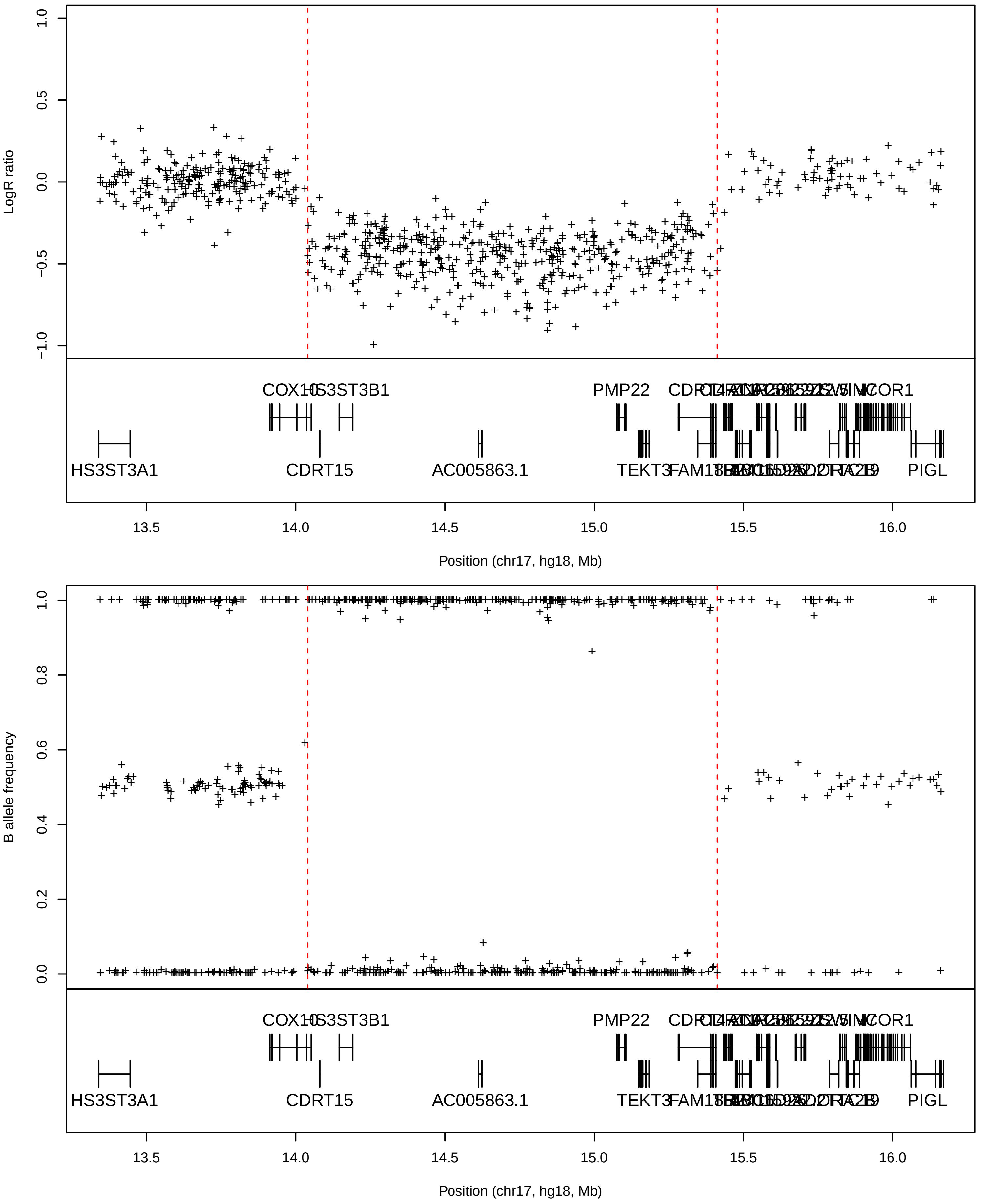

Figure S4−39, sample: 4378641488_R01C01, Internal ID:NA, COSTA RICA, case, chr17:32069090−33256513

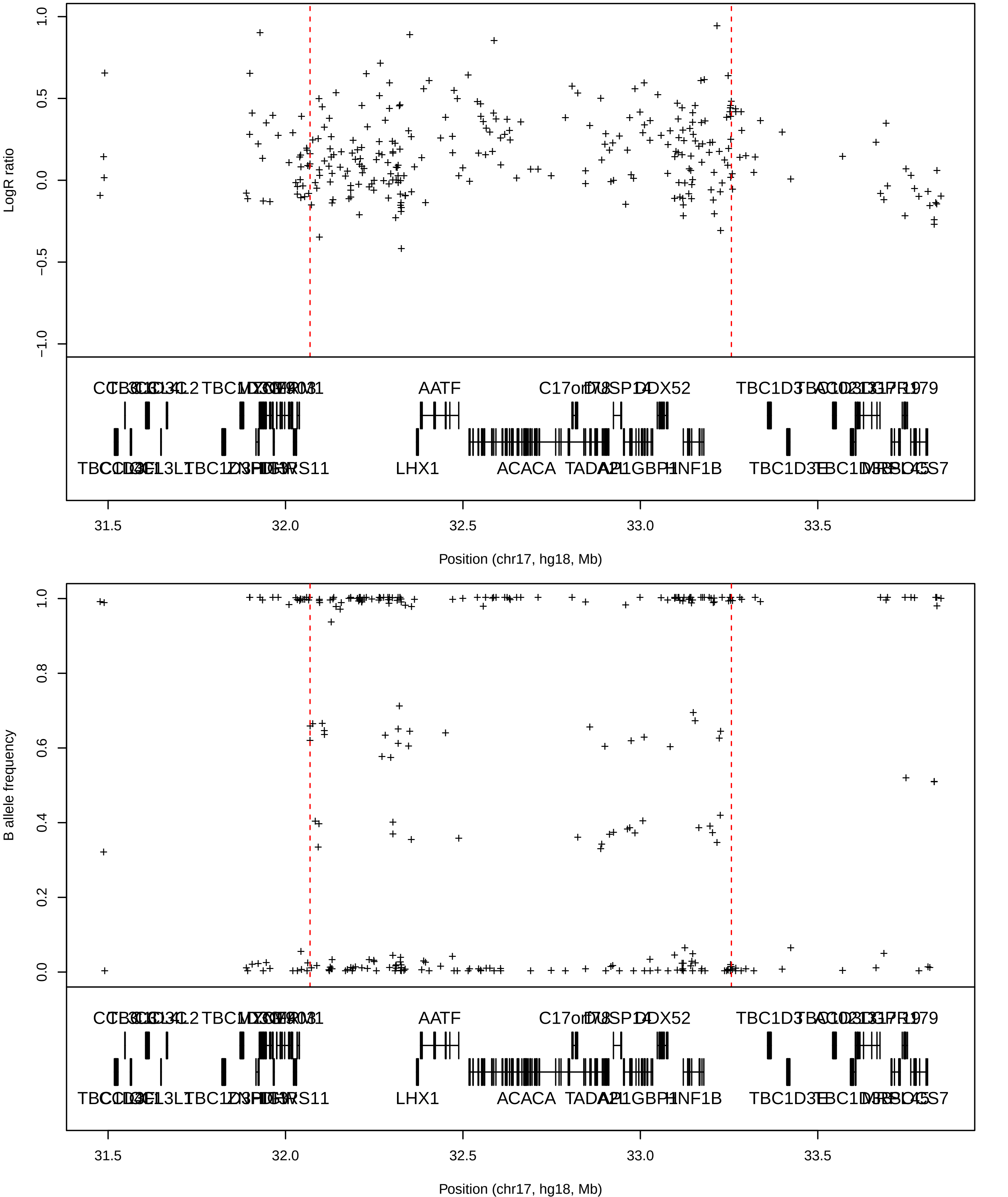

Figure S4−40, sample: 4343211465_R02C01, Internal ID:21.1, COLOMBIA, case, chr20:45918085−46531162

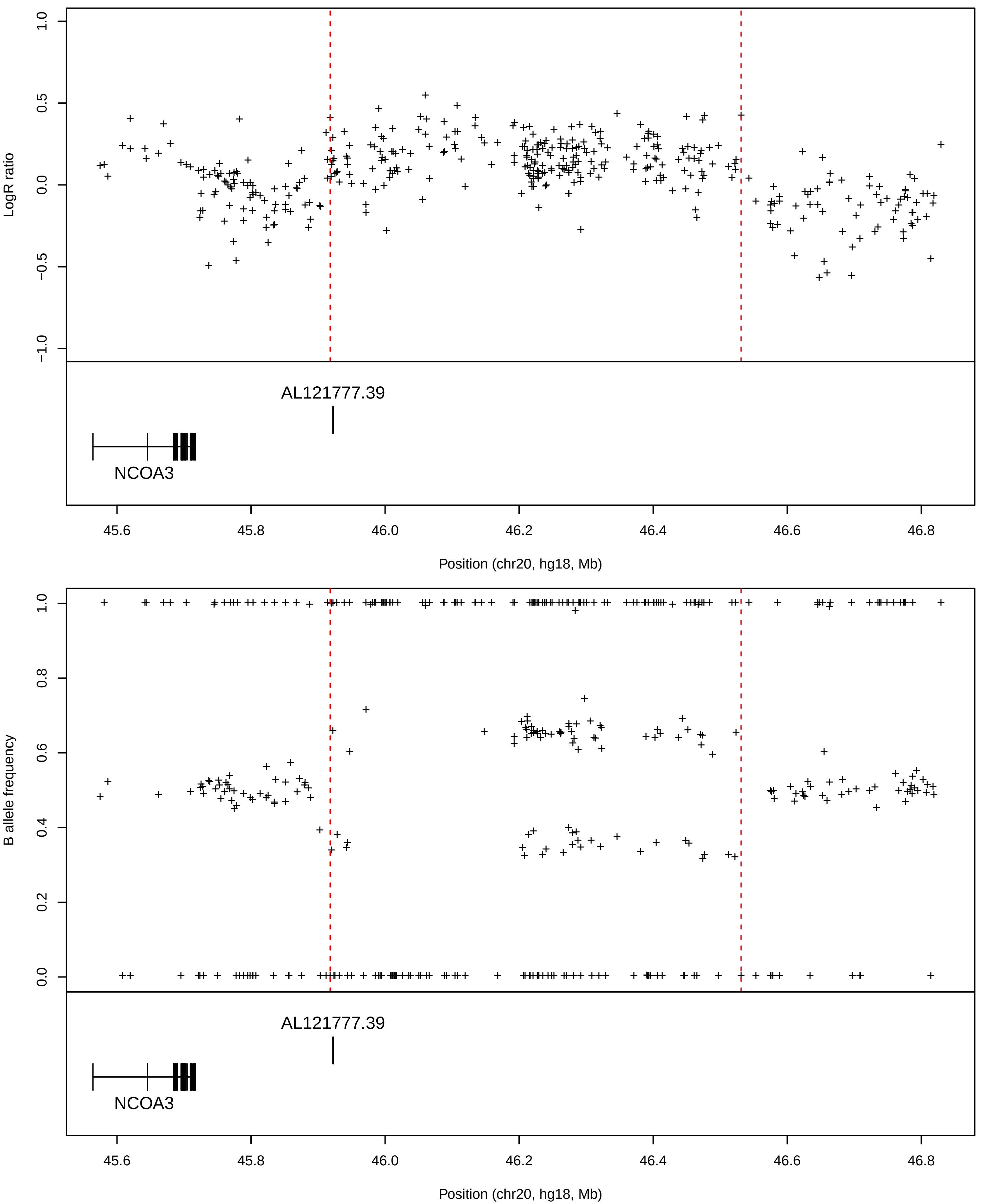

Figure S4−41, sample: 4506279006_R02C01, Internal ID:CP−117−2, COLOMBIA, control, chr21:13523286−14258290

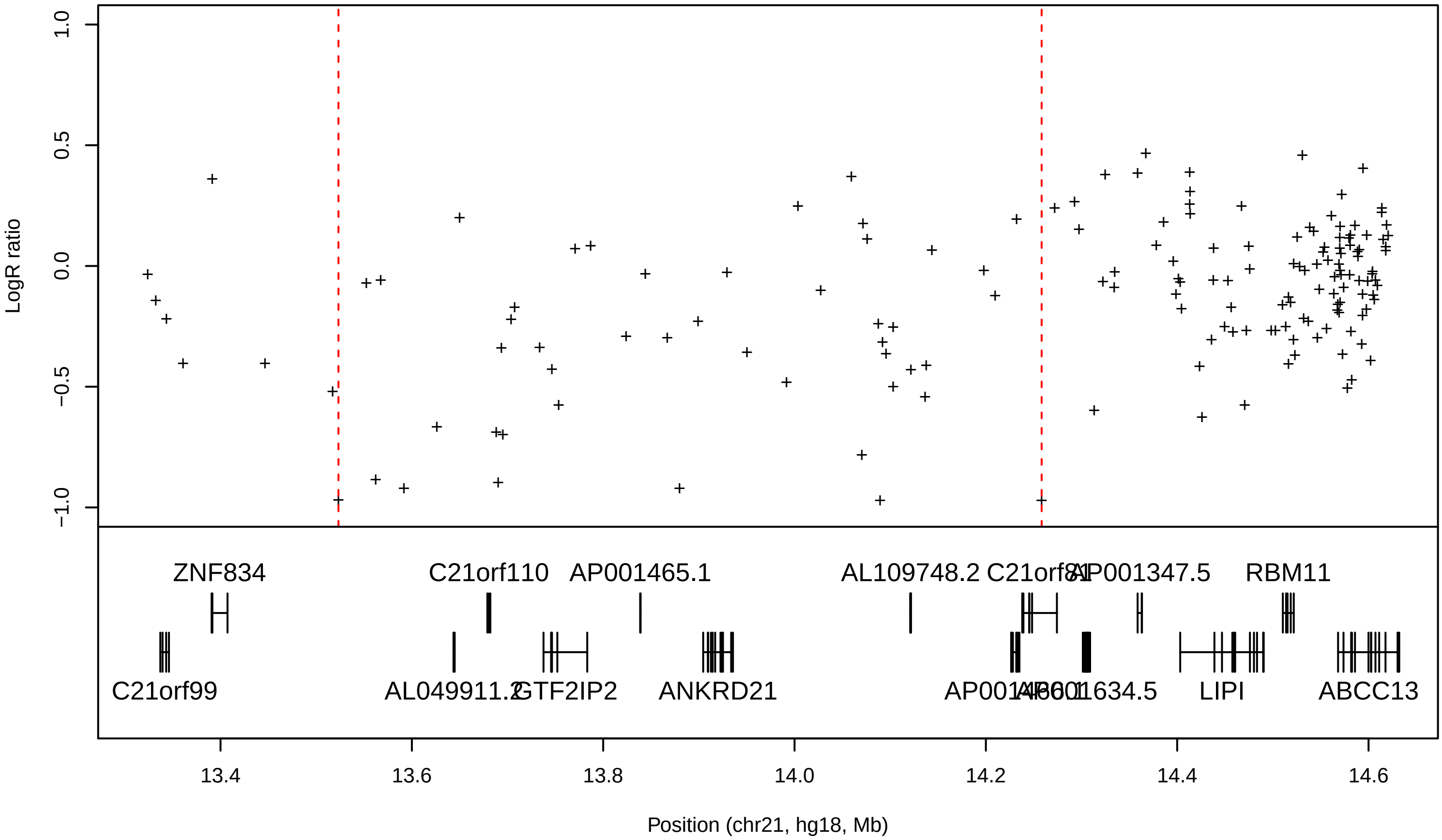

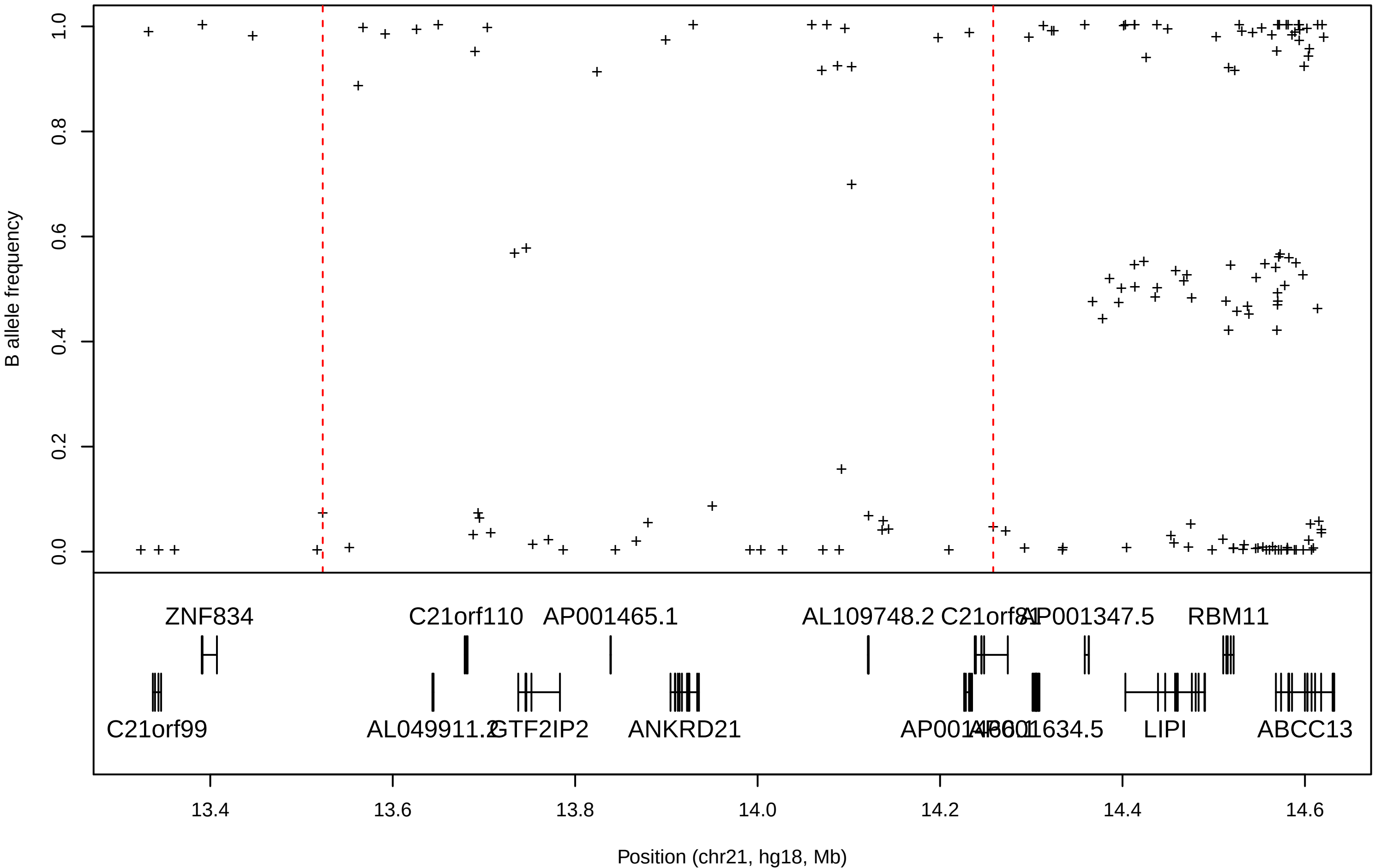

Figure S4−42, sample: 4515503133_R02C01, Internal ID:C317, COLOMBIA, control, chr21:13523286−14261969

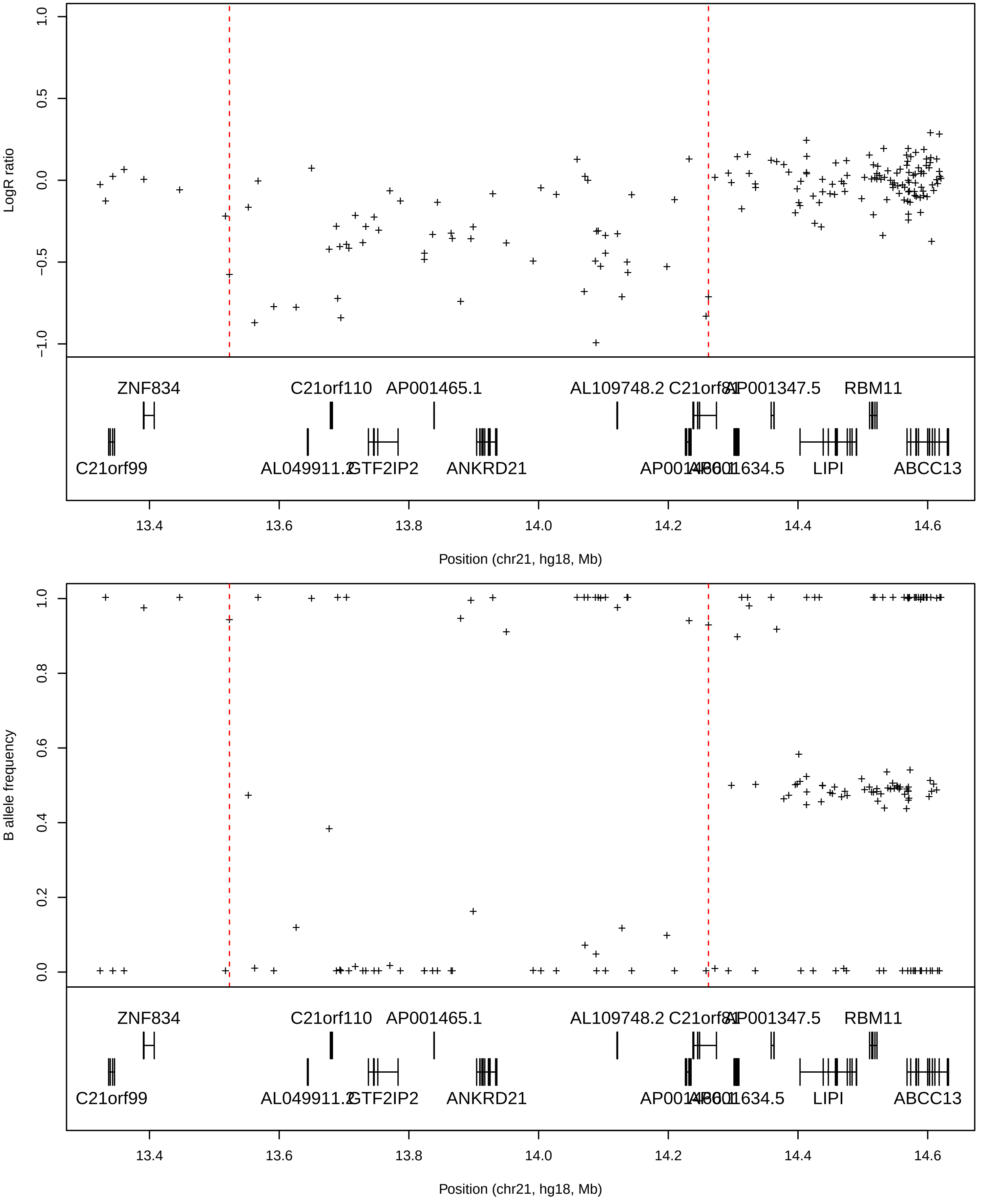

Figure S4−43, sample: 4378641817_R01C02, Internal ID:NA, COSTA RICA, case, chr22:17292678−19792353

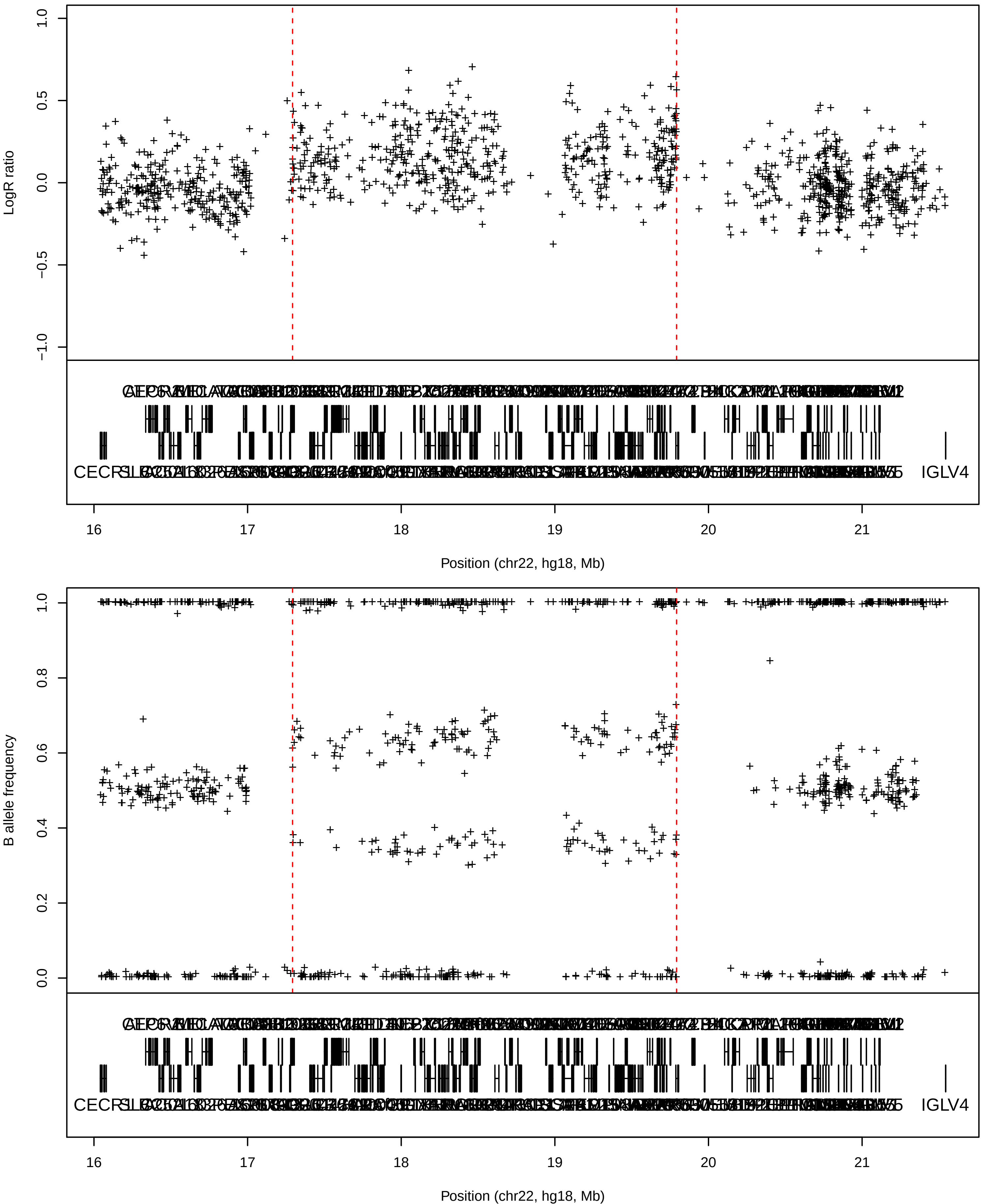

Figure S4−44, sample: 4506236100_R02C01, Internal ID:NA, COSTA RICA, case, chr22:46249374−46919985

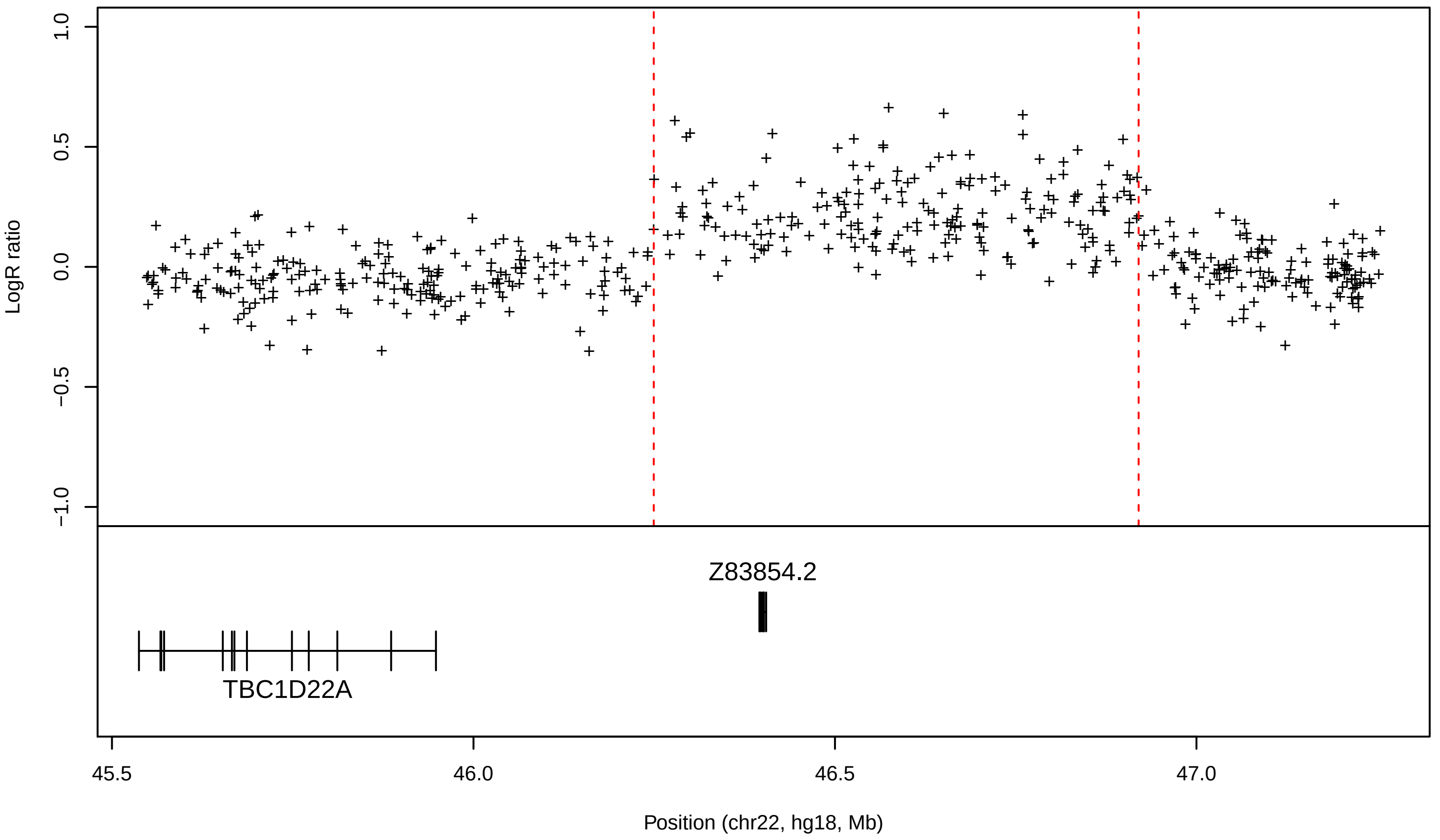

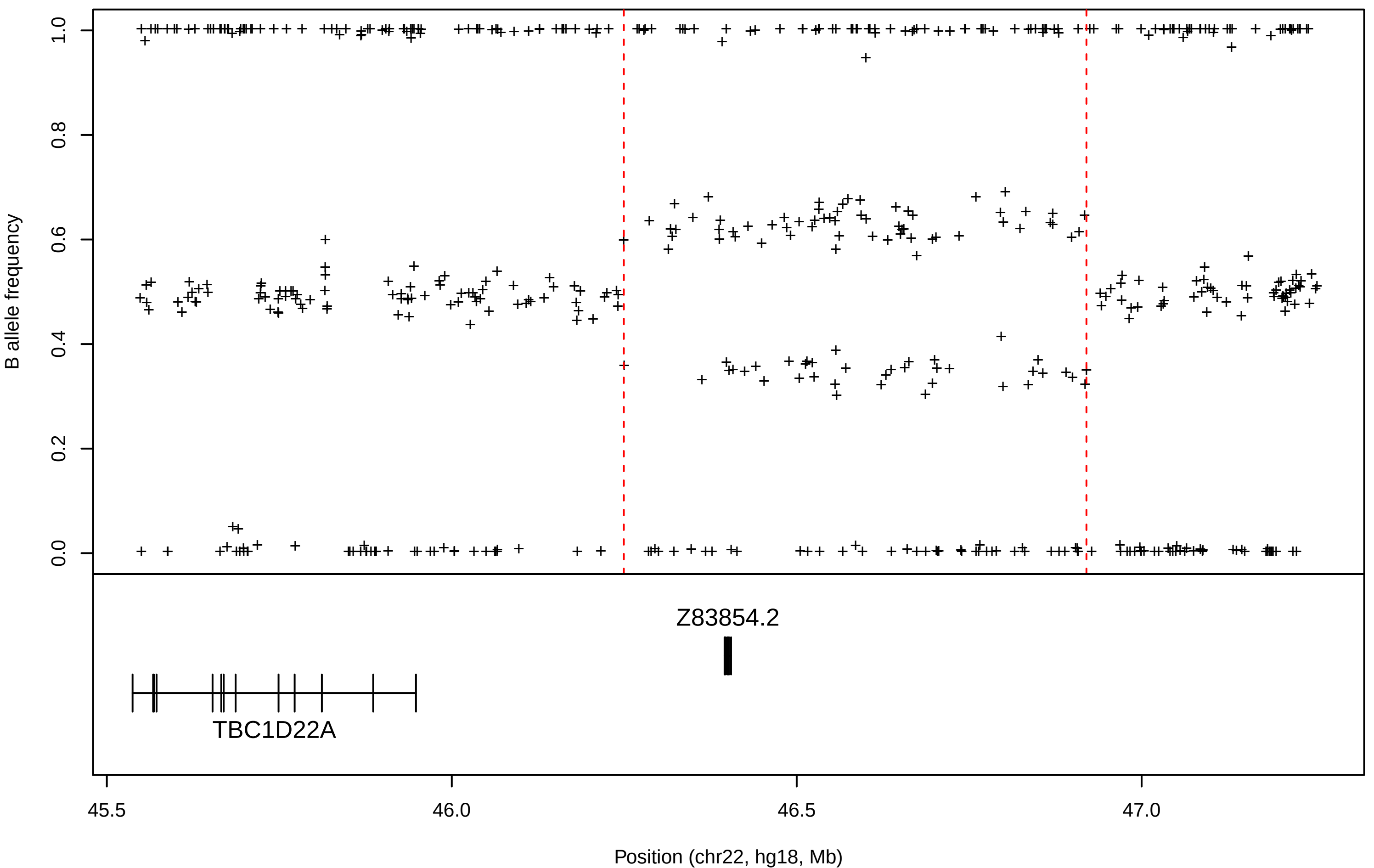

Supplement: Figure S4 — (1–44): Sample ID, population origin and case/control status are shown as figure heading. LogR ratio and B allele frequency are shown in the top and bottom panels, respectively. CNV boundaries are indicated by red dotted lines. Human RefSeq genes are shown below each panel (vertical lines indicating exons). Genomic position (in Mb) based on the hg18 human genome sequence. (PDF) [file pone.0059061.s004.pdf]
