# Supplementary material for: CNV Analysis in Tourette Syndrome Implicates Large Genomic Rearrangements in COL8A1 and NRXN1
Source: PLoS One. 2013 Mar 22;8(3):e59061. doi: 10.1371/journal.pone.0059061 (PMC3606459; doi:10.1371/journal.pone.0059061)

Figure S5: CNV calls using Multiplex Ligation-dependent Probe Amplification (MLPA)

Figure S5-1: Validation of the SNP-based CNV calls in *COL8A1* and *NRXN1* by MLPA

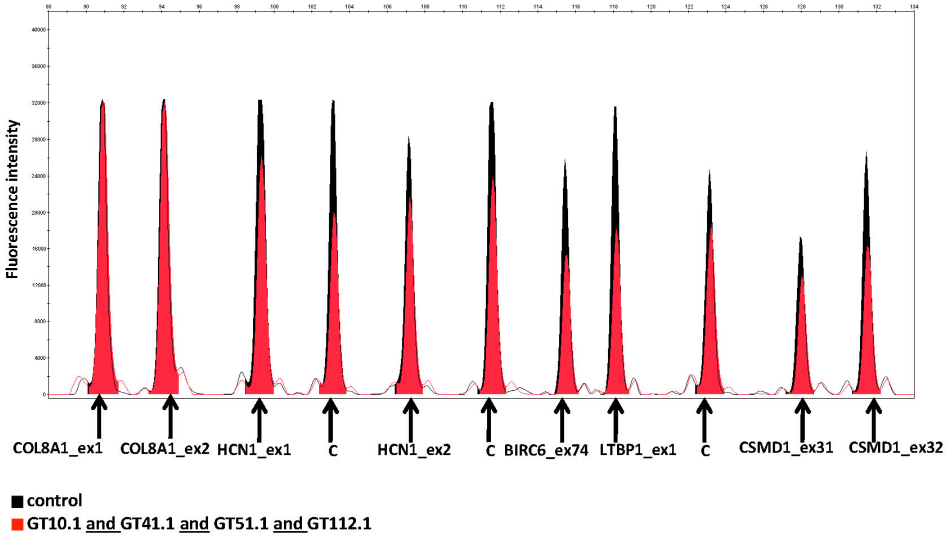

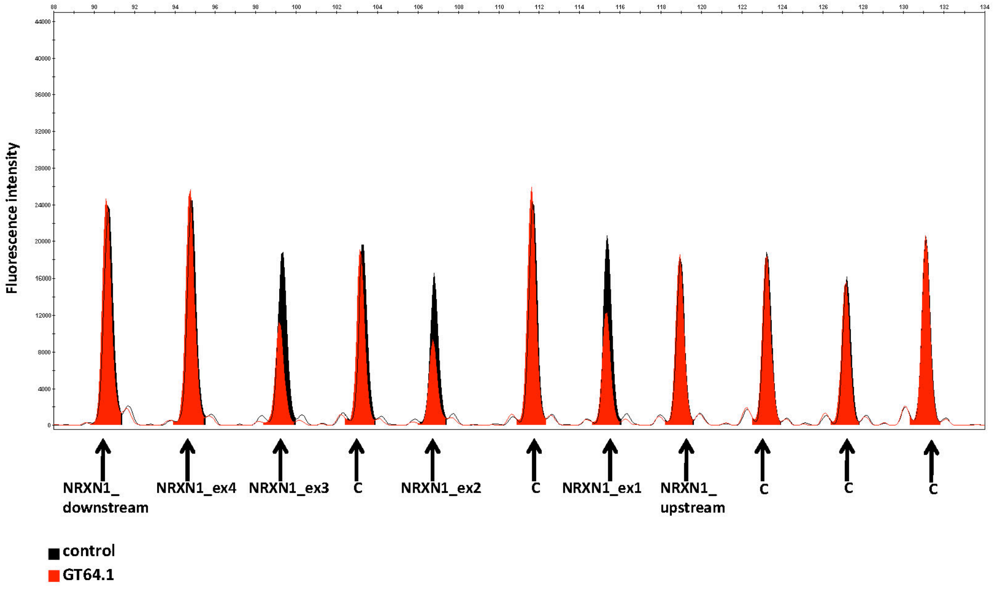

Figure S5-2: Detection of *de novo* deletions in *NRNX1* (exons 2 and 3) in TS cases.

A. Trio 5 (GT5.1=case, GT5.2=parent, GT5.3=parent)

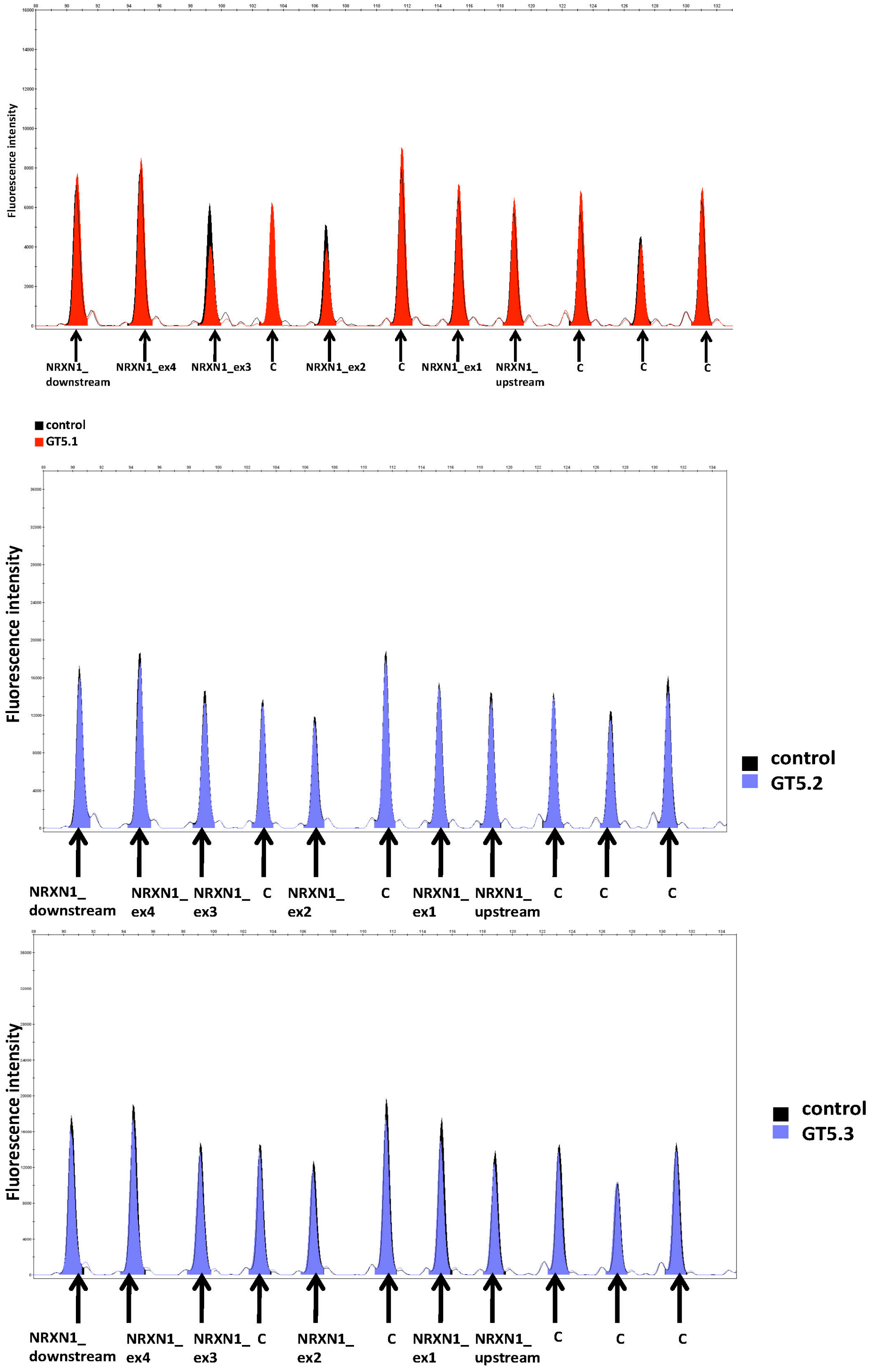

B. Trio 34 (GT34.1=case, GT34.2=parent, GT34.3=parent)

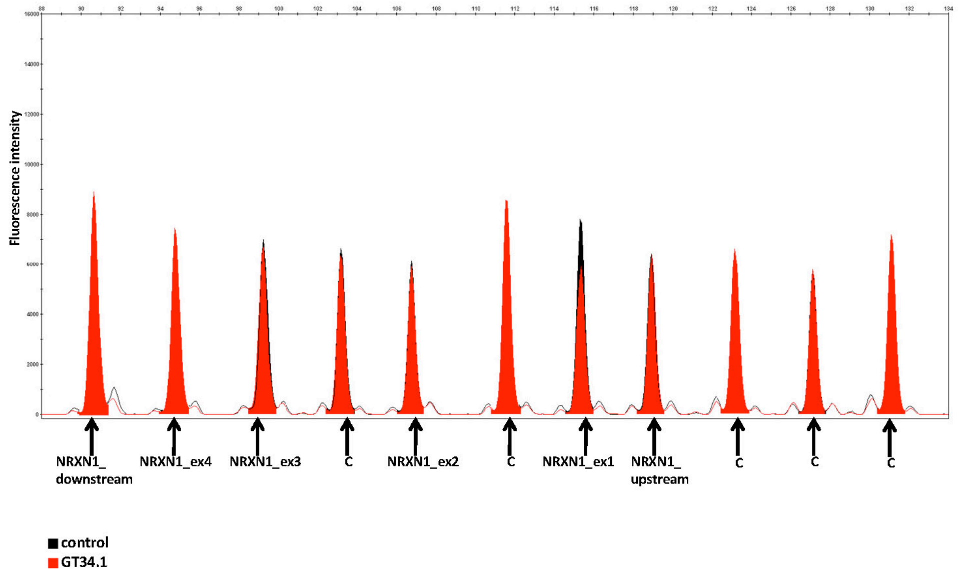

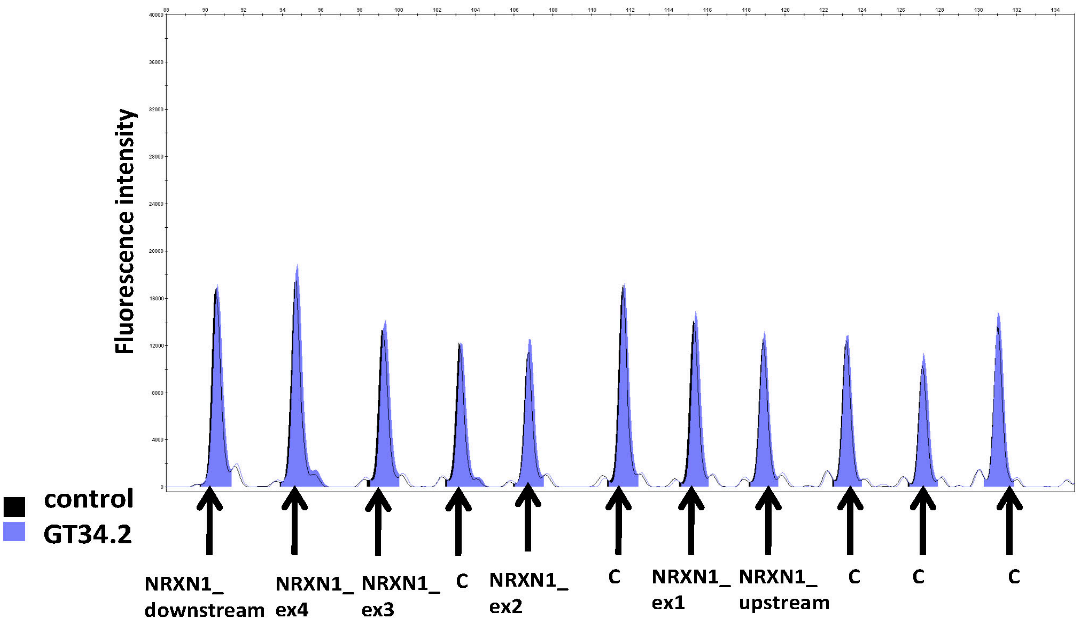

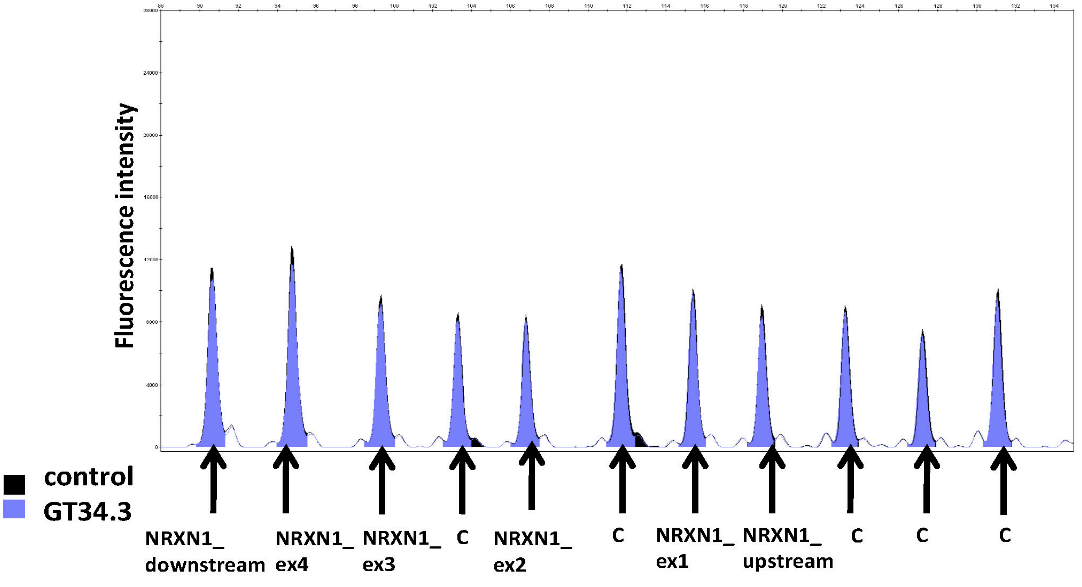

Figure S5-3: Two additional TS cases (GT5.1 and GT34.1) with deletions involving either exon 1, 2 or 3 of *NRXN1* detected by MLPA.

GT5.1 – heterozygous deletion of NRXN1 exons 2 and 3

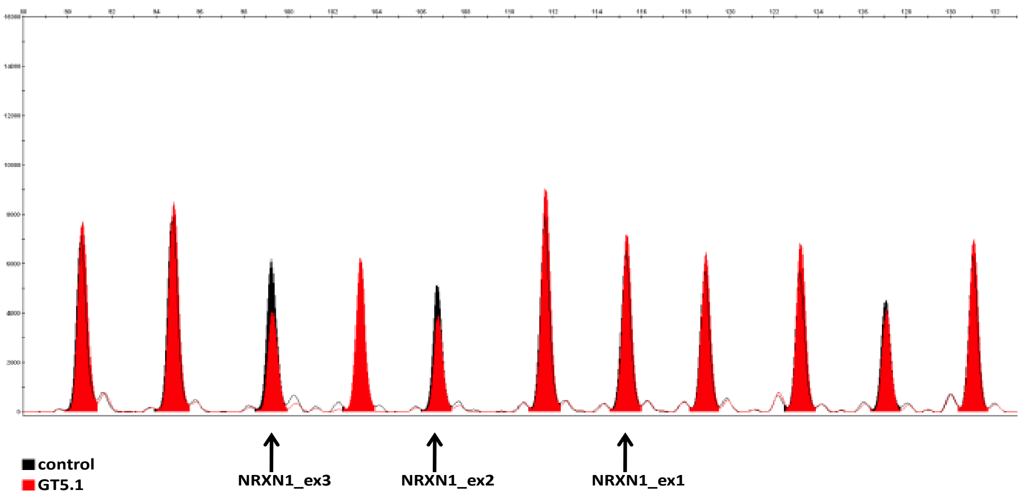

GT34.1 – heterozygous deletion of NRXN1 exon 1

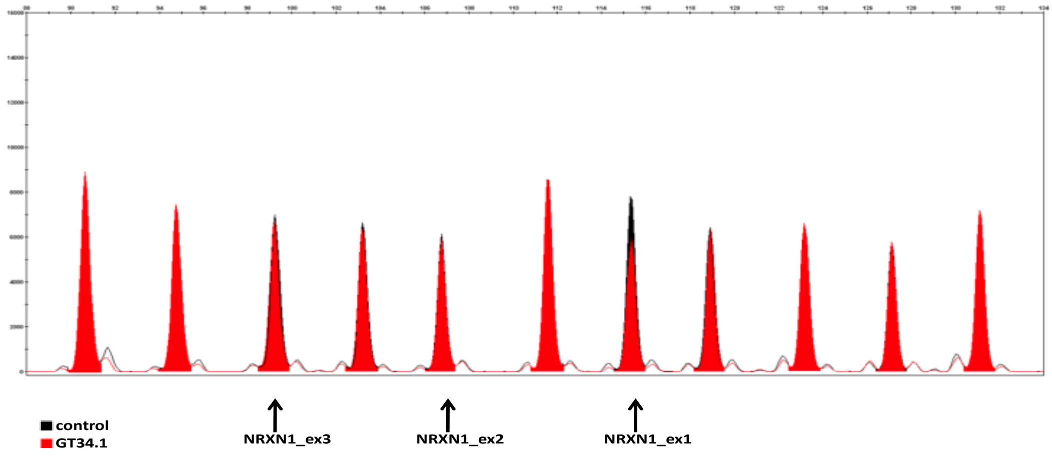

Figure S5-4: Three additional TS cases (GT7.1, GT29.1 and GT114.1) with deletion of exon 2 of *COL8A1* detected by MLPA.

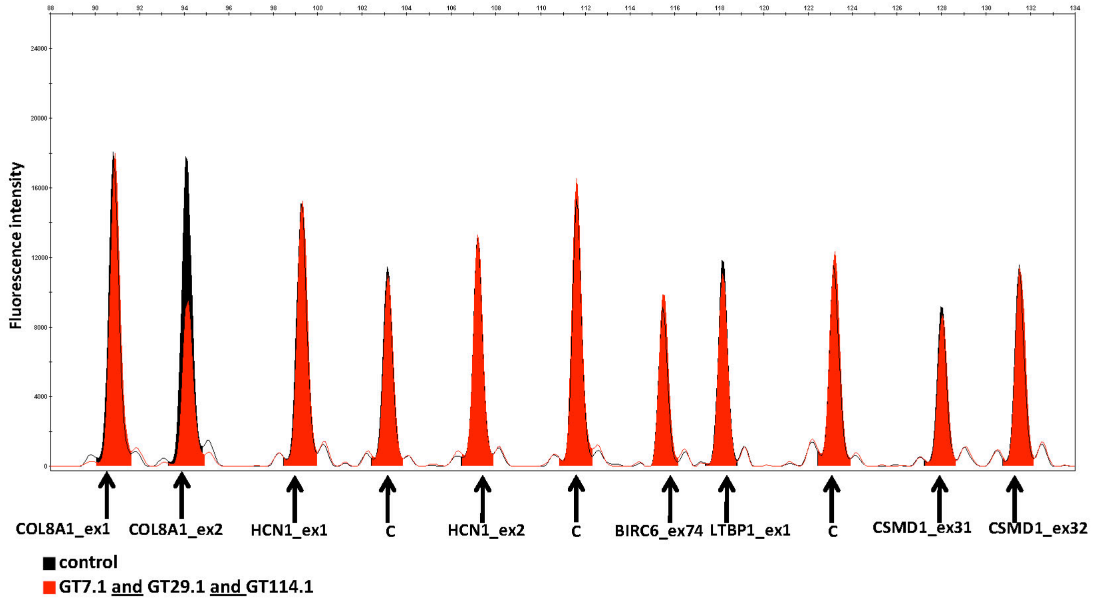

Supplement: Figure S5 — CNV calls using Multiplex Ligation-dependent Probe Amplification (MLPA). Figure S5-1: Validation of the SNP-based CNV calls in COL8A1 and NRXN1 by MLPA. Top panel: heterozygous duplication in COL8A1 (exons 1 and 2). Representative MLPA data and MLPA target probes for COL8A1 are shown. Bottom panel: Detection of a heterozygous deletion in NRNX1 (exons 1, 2, 3). MLPA target probes for NRXN1 are shown, the unlabelled target regions are probes located either on chromosome 2 but outside the deleted region or on other chromosomes (Table S2). Patient MLPA traces are in red, overlaid upon the normal control MLPA traces in black. Arrows point to the deleted/duplicated probes. Figure S5-2: Detection of de novo deletions in NRNX1 (exons 2 and 3) in TS cases. A, trio 5. B, trio 34. Patient MLPA traces are in red overlaid upon the normal control MLPA traces in black. The parents’ traces are in blue, overlaid upon normal controls in black. Arrows point to the MLPA probes in NRXN1. Figure S5-3: Two additional TS cases (GT5.1 and GT34.1) with deletions involving either exon 1, 2 or 3 of NRXN1 detected by MLPA. Representative MLPA data are shown. Patient traces are in red, overlaid upon the control traces in black. Arrows point to the MLPA probes in NRXN1. Figure S5-4: Three additional TS cases (GT7.1, GT29.1 and GT114.1) with deletion of exon 2 of COL8A1 detected by MLPA. Representative MLPA data are shown. Patient traces are in red, overlaid upon the control traces in black. Arrows point to the MLPA probes in COL8A1. (PDF) [file pone.0059061.s005.pdf]
