# Supplementary material for: CNV Analysis in Tourette Syndrome Implicates Large Genomic Rearrangements in COL8A1 and NRXN1
Source: PLoS One. 2013 Mar 22;8(3):e59061. doi: 10.1371/journal.pone.0059061 (PMC3606459; doi:10.1371/journal.pone.0059061)

Figure S6−1, sample: 4343211598_R02C02, Internal ID:5.1, COLOMBIA, case, chr2:51107671−51108957

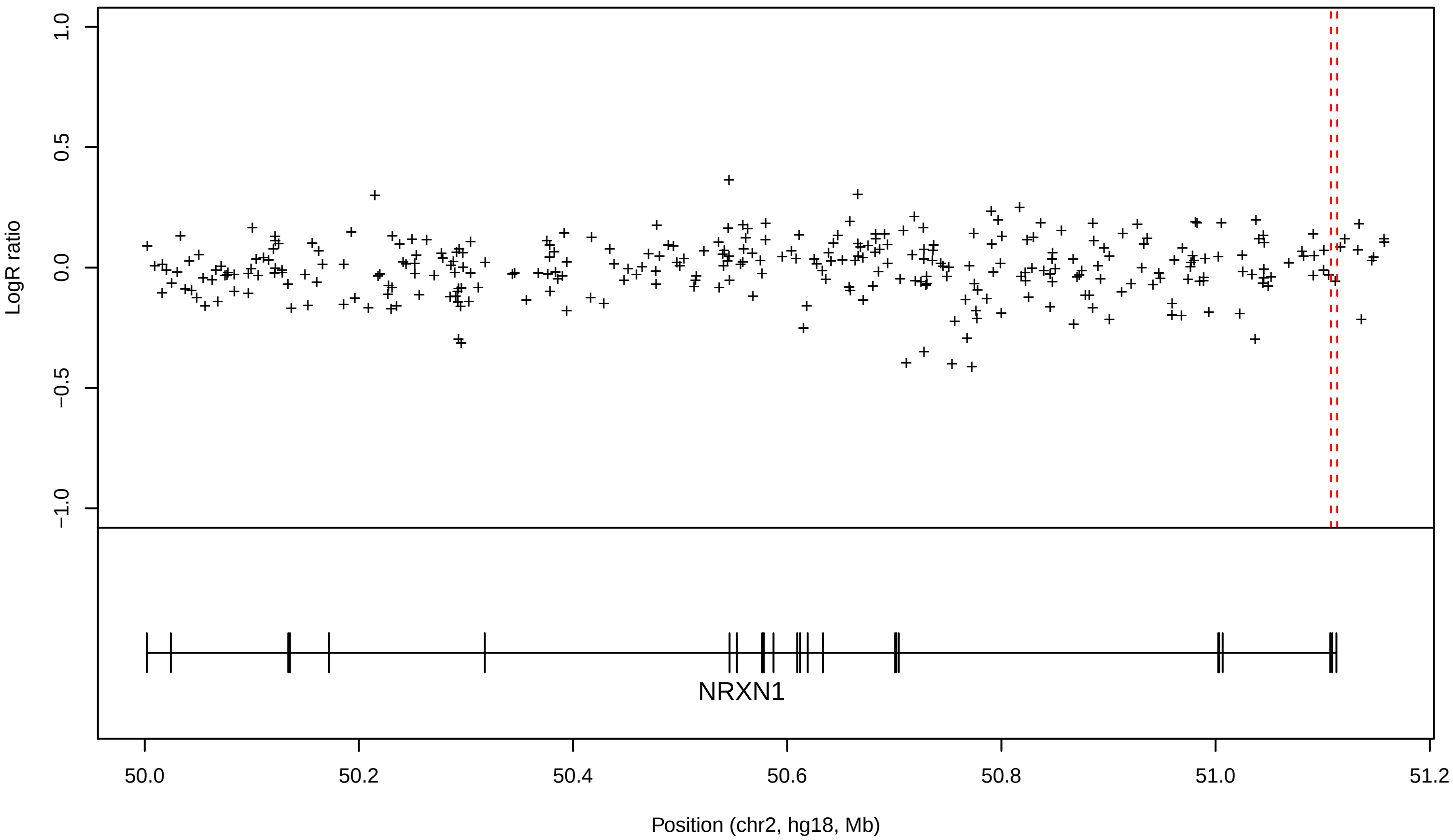

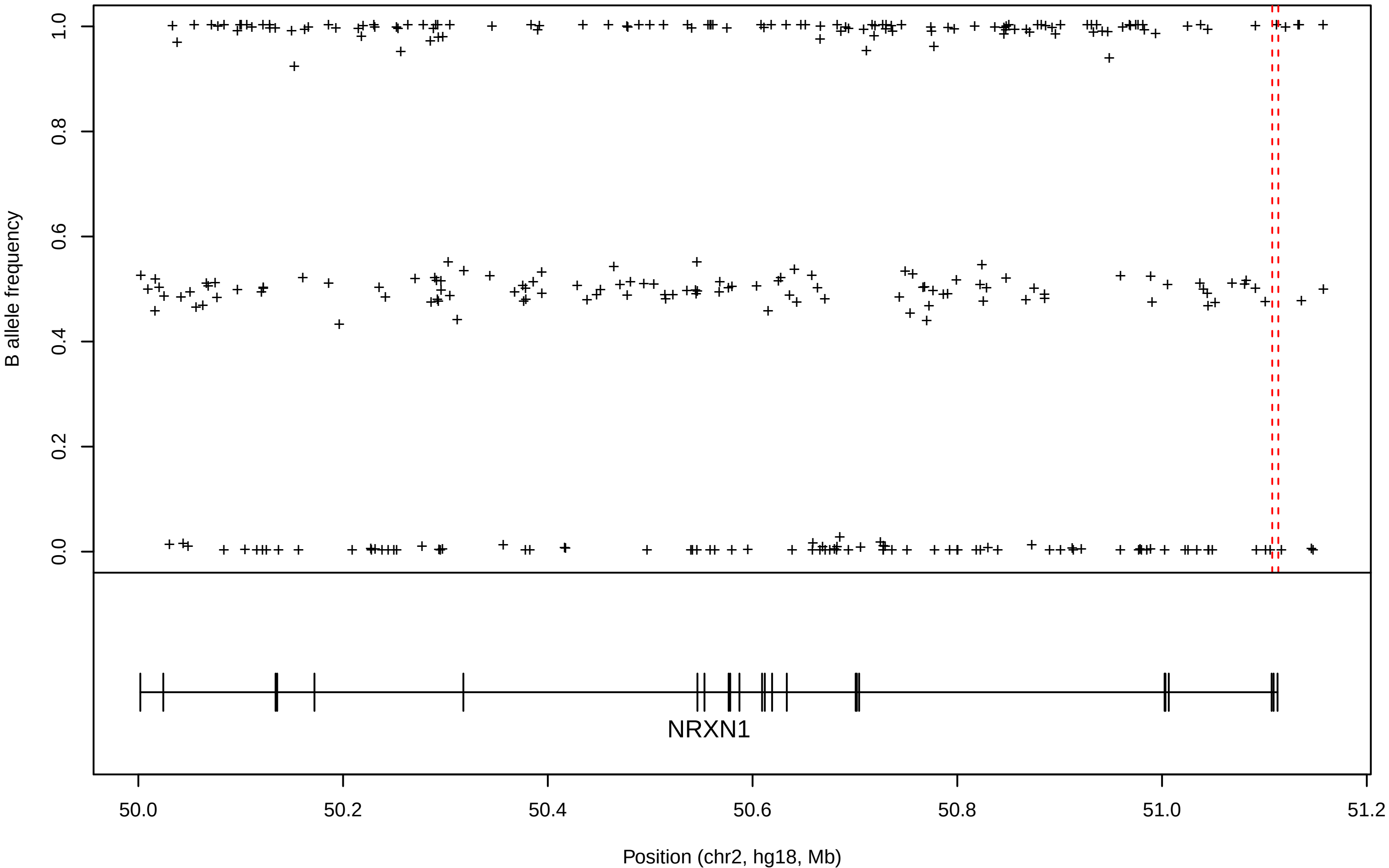

Figure S6−2, sample: 4506287023_R01C02, Internal ID:34.1, COLOMBIA, case, chr2:51113610−51113610

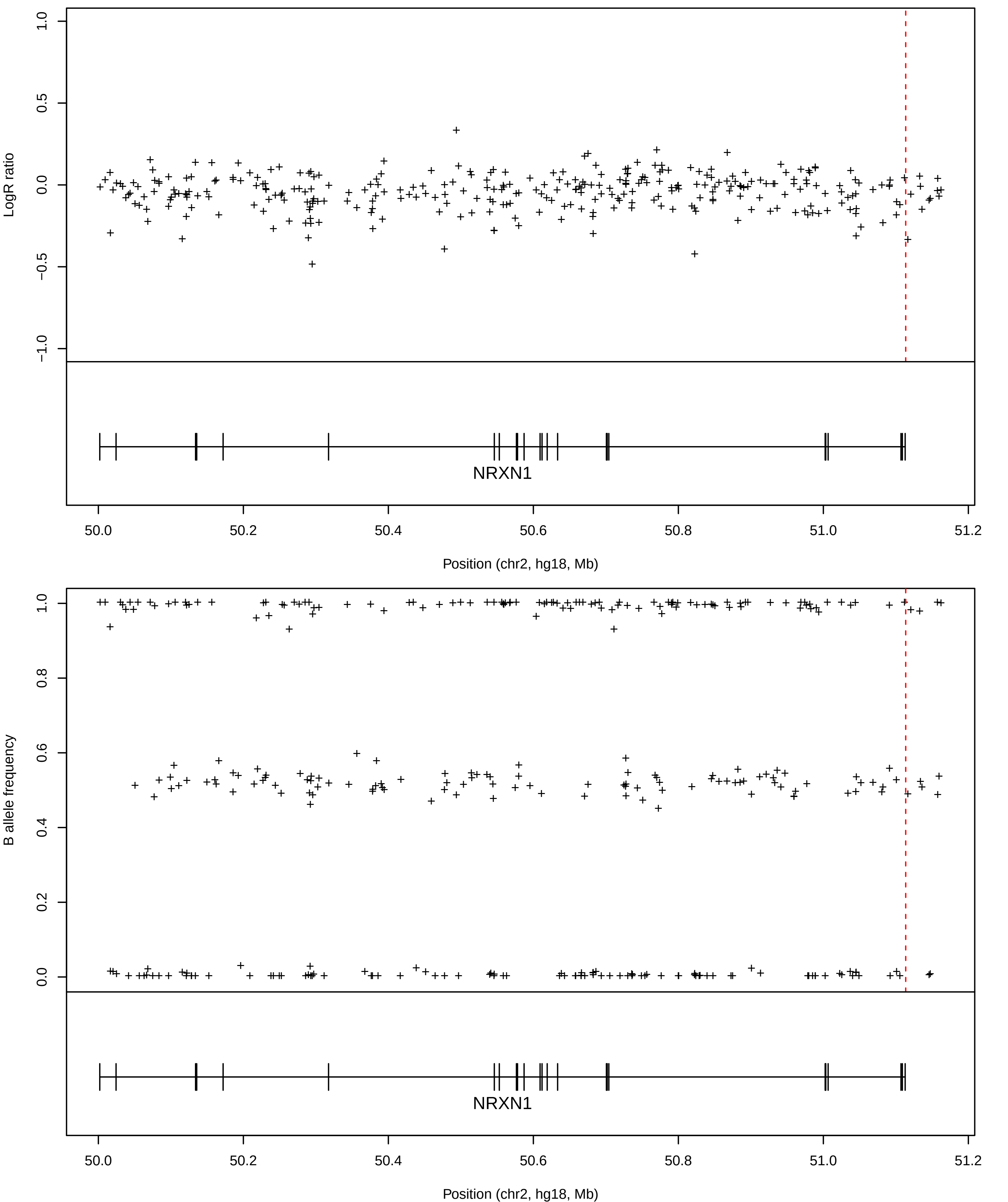

Figure S6−3, sample: 4506279157_R02C02, Internal ID:29.1, COLOMBIA, case, chr3:100877172−100877172

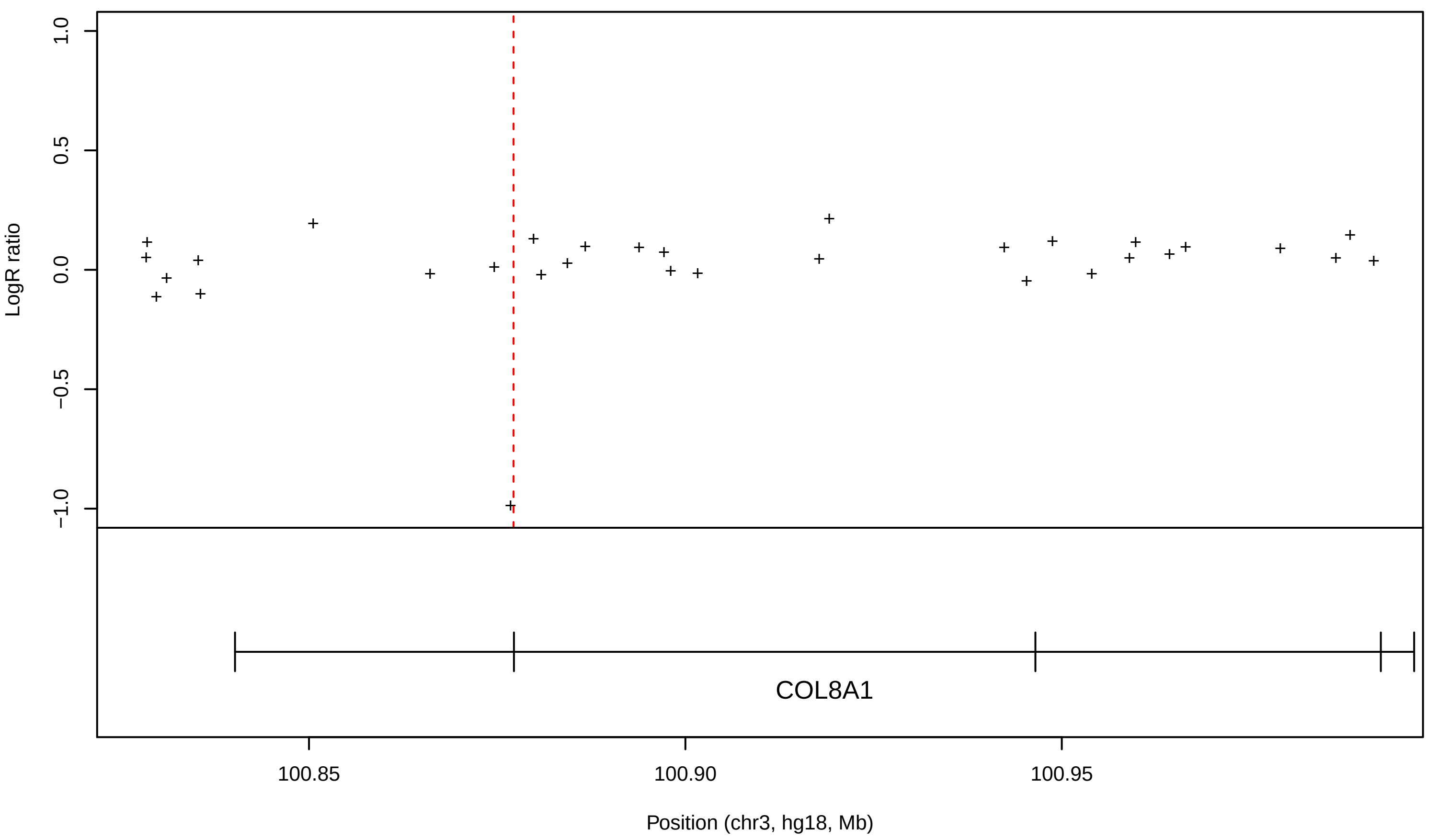

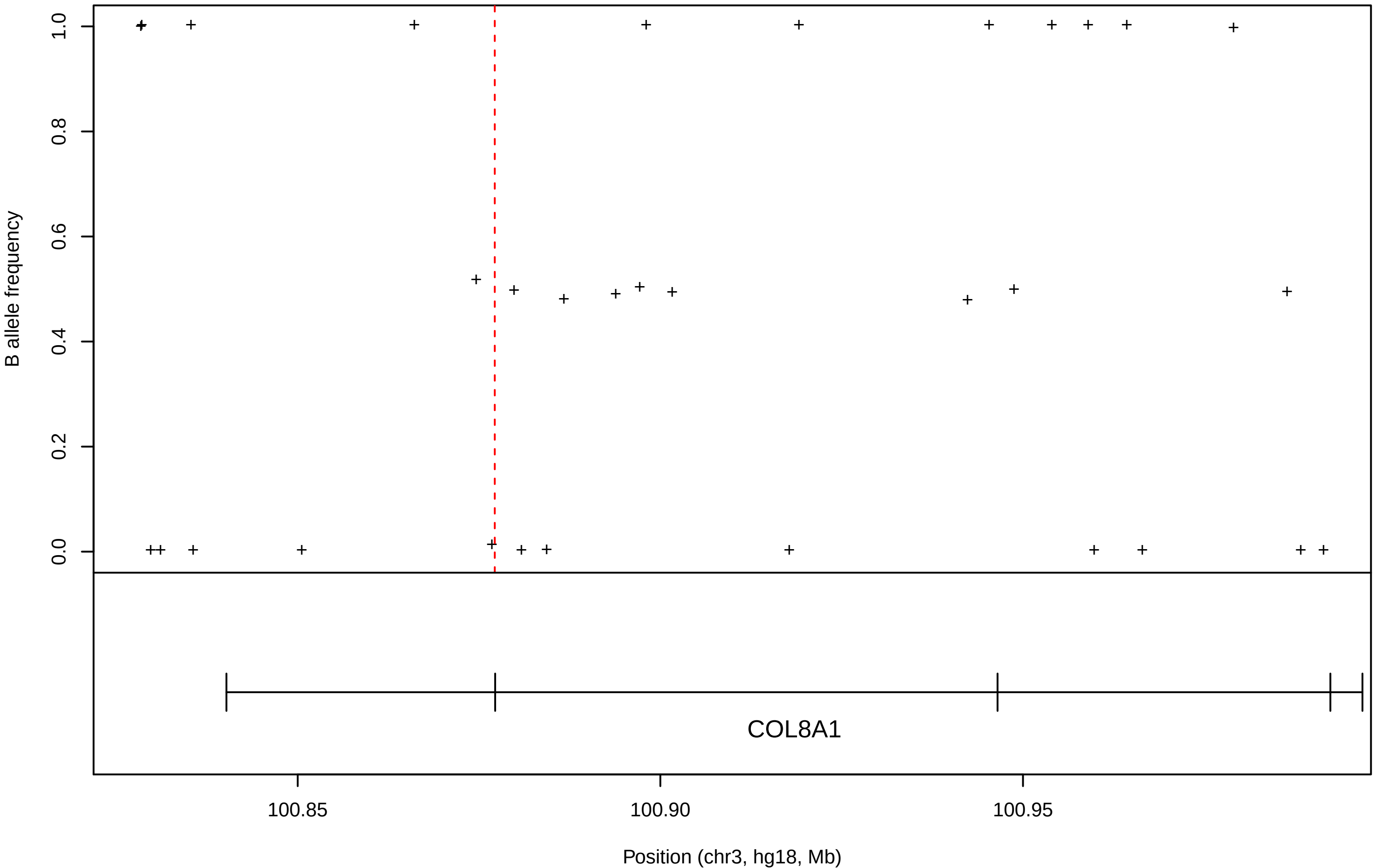

Figure S6−4, sample: 4506287081_R02C02, Internal ID:114.1, COLOMBIA, case, chr3:100877172−100877172

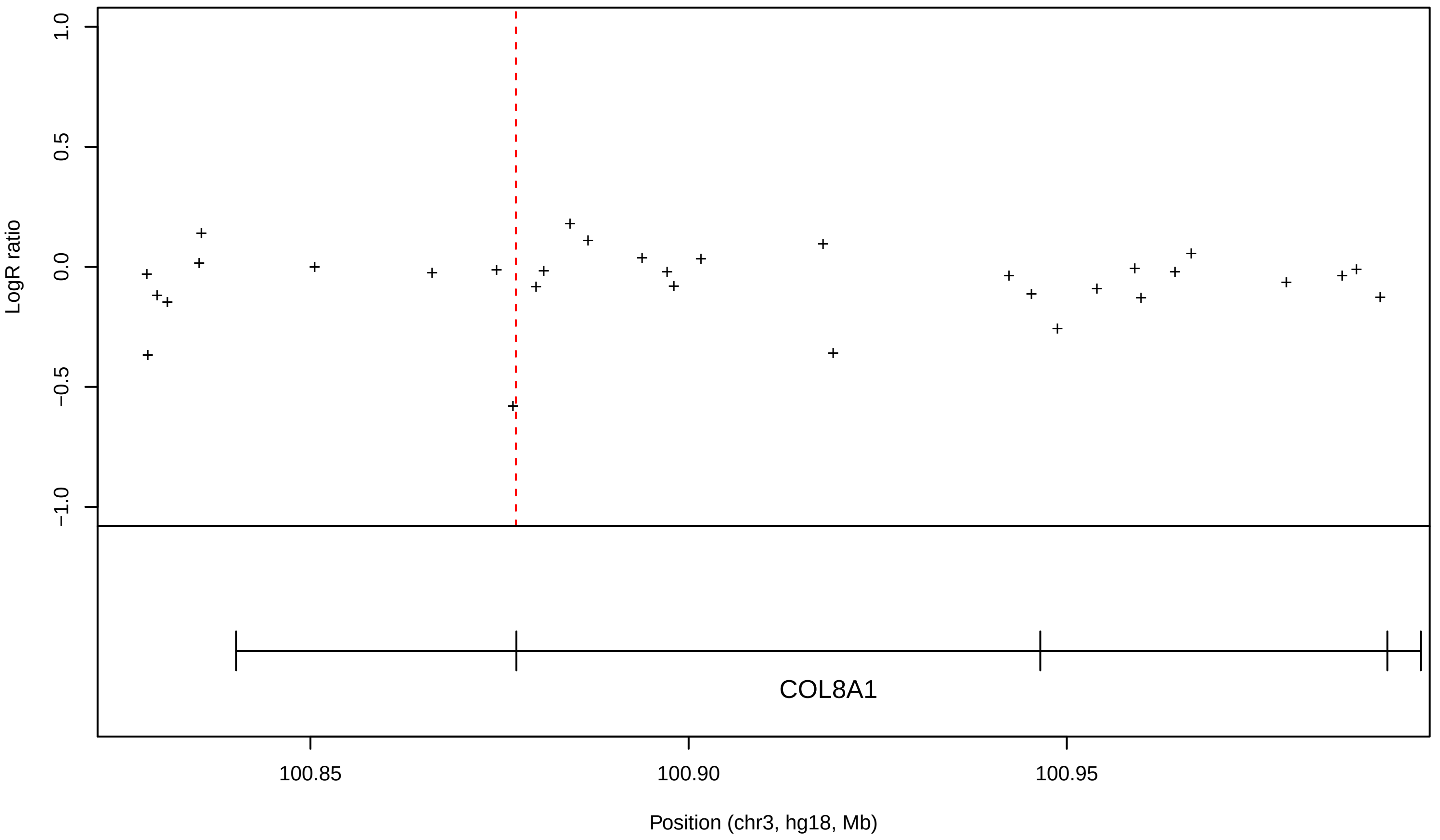

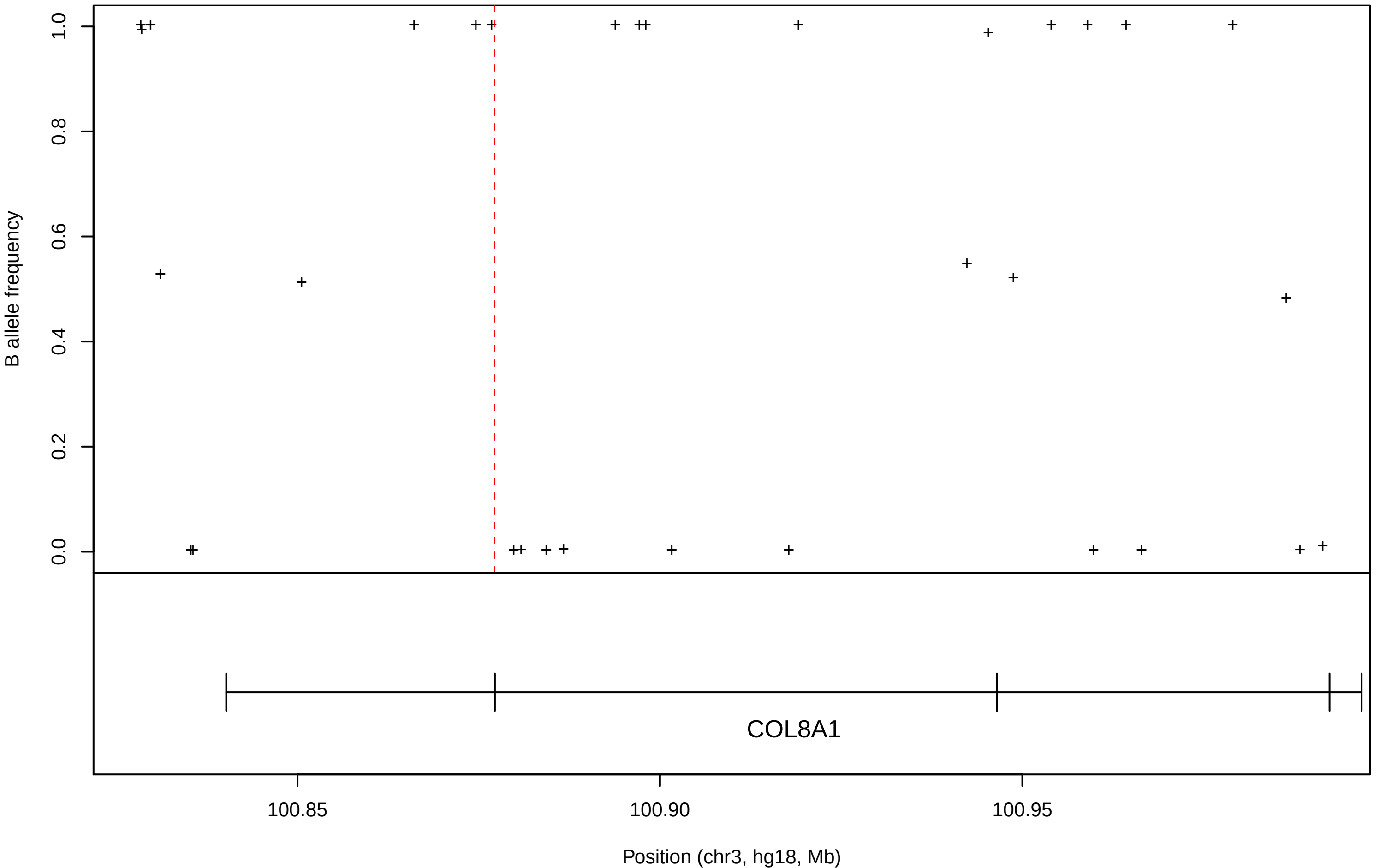

Figure S6−5, sample: 4393489021_R01C01, Internal ID:7.1, COLOMBIA, case, chr3:100877172−100877172

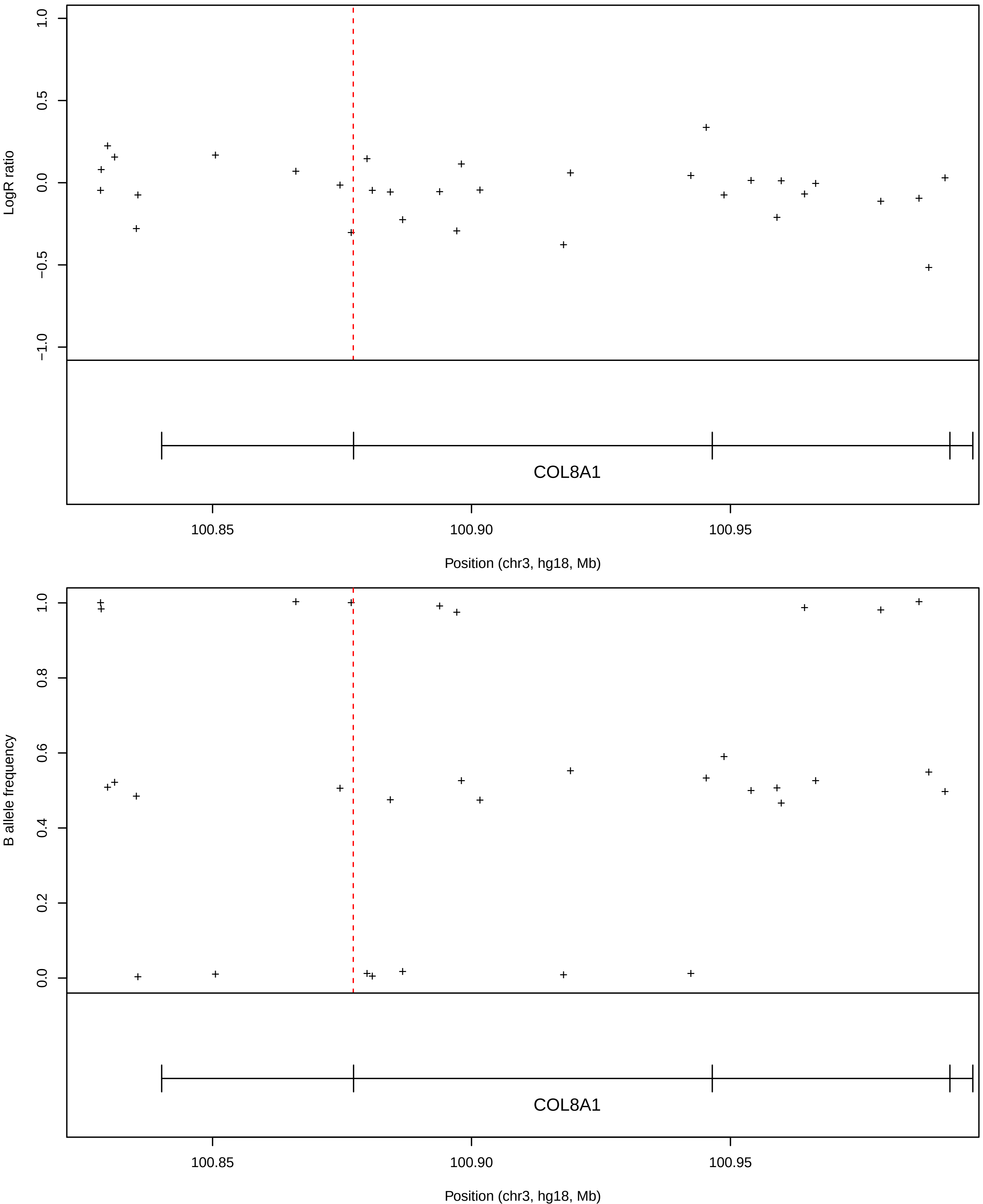

Supplement: Figure S6 — (1 to 5): Sample ID, population origin and case/control status are shown as figure heading. LogR ratio and B allele frequency are shown in the top and bottom panels, respectively. CNV boundaries are indicated by red dotted lines. The structure of NRXN1 (Figures S6-1 and S6-2) or COL8A1 (Figures S6-3 to S6-5) is shown below each panel with exons shown as vertical lines. Genomic position (in Mb) provided make use of the hg18 human genome sequence as reference. (PDF) [file pone.0059061.s006.pdf]
