# Supplementary material for: CNV Analysis in Tourette Syndrome Implicates Large Genomic Rearrangements in COL8A1 and NRXN1
Source: PLoS One. 2013 Mar 22;8(3):e59061. doi: 10.1371/journal.pone.0059061 (PMC3606459; doi:10.1371/journal.pone.0059061)
Supplement: Table S1 — Chromosomal regions harbouring large (>500 kb) CNVs overlapping annotated gene exons in TS cases but not in controls. aDup = duplication; b According to build 36 of the human genome. (DOC) [file pone.0059061.s007.doc]

**Table S1: Chromosomal regions harbouring large (>500 kb) CNVs overlapping
 annotated gene exons in TS cases but not in controls**

| **Location** | **CNV**  **Type^a^** | **Start position^b^** | **End position** | **Size** | **# of markers** | **Gene(s)^b^** | **Figure(s)** |
| --- | --- | --- | --- | --- | --- | --- | --- |
| 1p36.13 | Dup | 18,022,097 | 18,576,259 | 554,163 | 189 | *ACTL8,*  *IGSF21* | S4-1 |
| 2p22.3 | Dup | 32,487,194 | 33,186,442 | 699,249 | 145 | *BIRC6,*  *TTC27,LTBP1* | S4-4 |
|  | Dup | 32,487,194 | 33,174,461 | 687,268 | 134 | *BIRC6,*  *TTC27,LTBP1* | S4-5 |
| 2p25.3 | Dup | 333,588 | 1,004,837 | 671,250 | 142 | *SNTG2, TMEM18* | S4-3 |
| 3q12.1 | Dup | 100,269,291 | 100,876,782 | 607,492 | 105 | *COL8A1* | S4−9 |
|  | Dup | 100,269,291 | 100,886,715 | 617,425 | 113 | *COL8A1* | S4−10 |
|  | Dup | 100,269,291 | 100,886,715 | 617,425 | 108 | *COL8A1* | S4−11 |
|  | Dup | 100,249,016 | 100,886,715 | 637,700 | 105 | *COL8A1* | S4−12 |
| 3p26.3 | Dup | 852,235 | 1,403,635 | 551,401 | 216 | *CNTN6* | S4-8 |
| 4p15.32 | Dup | 16,443,738 | 17,281,982 | 838,245 | 168 | *QDPR,*  *CLRN2, LAP3,*  *LDB2, MED28* | S4-14 |
| 4q25 | Dup | 108,061,142 | 108,572,634 | 511,493 | 91 | *DKK2* | S4-18 |
| 5p12 | Dup | 45,400,632 | 46,196,544 | 795,913 | 69 | *HCN1* | S4-20 |
| 5q21.1 | Dup | 101,503,405 | 102,033,686 | 530,282 | 66 | *SLCO4C1, SLCO6A1* | S4-21 |
|  | Dup | 101,532,676 | 102,033,686 | 501,011 | 70 | *SLCO4C1, SLCO6A1* | S4-22 |
| 10p12.31 | Dup | 20,010,963 | 20,578,907 | 567,945 | 186 | *PLXDC2* | S4-32 |
| 12q12 | Dup | 38,586,180 | 39,111,794 | 525,575 | 153 | *LRRK2, SLC2A13* | S4-37 |
| 17q12 | Dup | 32,069,090 | 33,256,513 | 1,187,424 | 222 | *AATF, LHX1, ACACA, SYNRG, HNF1B* | S4-39 |
| 22q11.21 | Dup | 17,292,678 | 19,792,353 | 2,499,676 | 506 | *DGCR5, DGCR8, PRODH, P2RX6, COMT, BCRP2* | S4-43 |

^a^Dup = duplication; ^b^ According to build 36 of the human genome
