# Supplementary material for: CNV Analysis in Tourette Syndrome Implicates Large Genomic Rearrangements in COL8A1 and NRXN1
Source: PLoS One. 2013 Mar 22;8(3):e59061. doi: 10.1371/journal.pone.0059061 (PMC3606459; doi:10.1371/journal.pone.0059061)
Supplement: Table S2 — Target probes used in the MLPA assay. (DOC) [file pone.0059061.s008.doc]

**Table S2: Target probes used in the MLPA assay**

| **Target** | **Location (Build 36)** |
| --- | --- |
| FSHR (downstream NRXN1) | chr2:49,043,581-49,043,631 |
| NRXN1-exon 4 | chr2:50,703,926-50,703,980 |
| NRXN1-exon 3 | chr2:51,107,613-51,107,671 |
| NRXN1-exon 2 | chr2:51,108,957-51,109,023 |
| NRXN1-exon 1 | chr2:51,113,610-51,113,684 |
| upstream NRXN1 | chr2:51,715,137-51,715,215 |
| COL8A1-exon 1 | chr3:100,839,359-100,839,409 |
| COL8A1-exon 2 | chr3:100,877,172-100,877,226 |
