# Supplementary material for: CNV Analysis in Tourette Syndrome Implicates Large Genomic Rearrangements in COL8A1 and NRXN1
Source: PLoS One. 2013 Mar 22;8(3):e59061. doi: 10.1371/journal.pone.0059061 (PMC3606459; doi:10.1371/journal.pone.0059061)
Supplement: Methods S1 — CNV Quality Control and CNV validation by Multiplex ligation-dependent probe amplification (MLPA). (DOCX) [file pone.0059061.s009.docx]

**Supplementary Methods**

**CNV Quality Control**

Stringent quality control (QC) measures were implemented in order to ensure that only good quality samples are used for analysis. High values for the standard deviation of normalised intensity (LRR_SD) or high number of CNV calls (NumCNV) are indicators of poor sample quality and such samples were excluded from further analyses. A plot of the number of CNV calls versus the standard deviation of normalised intensity for the samples was thus used to set the threshold values as NumCNV = 30 and LRR_SD = 0.24 (Figures S2(1-2)). Wave artifacts that correlate with the GC content of the probes are known to interfere with CNV calling algorithms. To eliminate samples with extreme wave factors (WF), the default PennCNV threshold of WF=0.05 was implemented. A pair-wise IBD (identity by descent) estimation detected no two pair of individuals with a proportion IBD (PI_HAT) value higher than 0.09. All the samples had SNP genotype call rate values ranging from 99.92% to 99.96%. (Figure S2(3)).

**CNV validation by Multiplex ligation-dependent probe amplification (MLPA)**

MLPA probes were designed following MRC-Holland recommendations (http://www.mlpa.com/files/protocol_synthetic_probe_design.pdf) and are listed in Supplementary S2. A minimum of 50 ng of genomic DNA was used for the MLPA reaction, carried out with the SALSA-MLPA kit (MRC-Holland, The Netherlands) according to the manufacturer’s instructions. PCR products were subsequently subjected to capillary electrophoresis on an ABI 3130 genetic analyzer (Applied Biosystems, UK) and electrophoresis data extracted using GeneMapper software v4.0 (Applied Biosystems, UK). Population controls normalization using relative peak height (RPH) method recommended by MRC-Holland was utilized for data analysis (Schouten et al. Nucleic Acids Res 2002; 30: e57 and Slater et al. 2004 Hum Mutat 2004; 24: 164–171). A threshold for gene dosage of ≤ 0.75 (loss) and ≥1.30 (gain) was applied for CNV validation.
